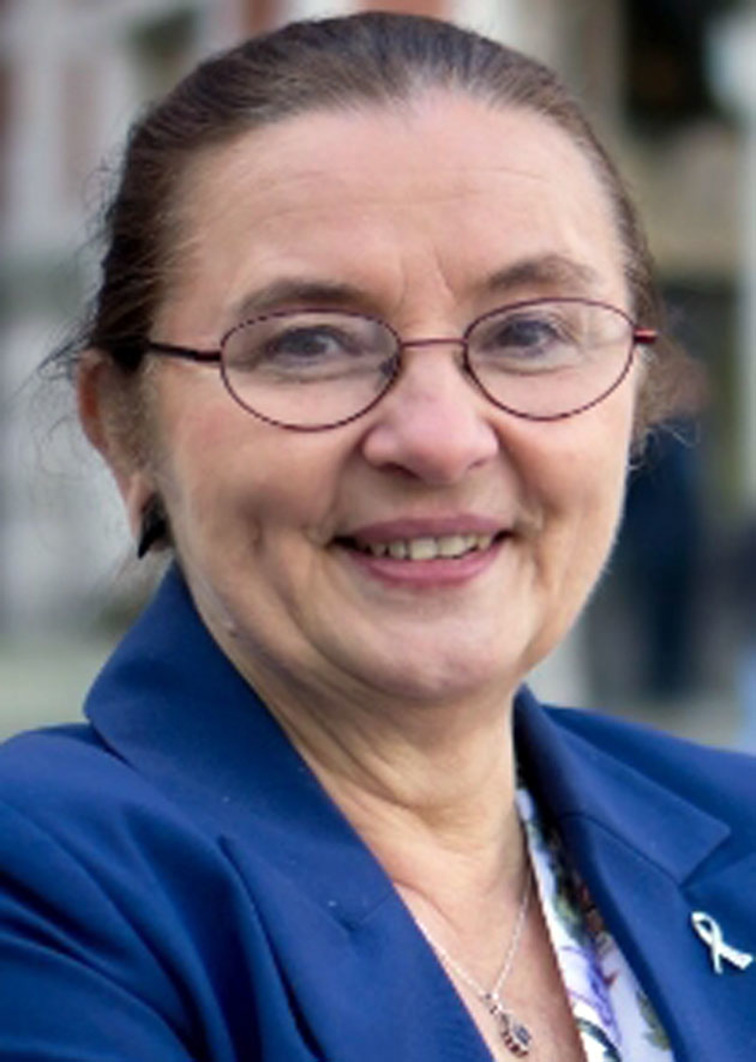# Amino Acid Based Antimicrobial Agents – Synthesis and Properties

**DOI:** 10.1002/cmdc.202100503

**Published:** 2021-10-01

**Authors:** Michał G. Nowak, Andrzej S. Skwarecki, Maria J. Milewska

**Affiliations:** ^1^ Department of Organic Chemistry and BioTechMed Center Gdańsk University of Technology 11/12 Gabriela Narutowicza Street 80-233 Gdańsk Poland; ^2^ Department of Pharmaceutical Technology and Biochemistry and BioTechMed Center Gdańsk University of Technology 11/12 Gabriela Narutowicza Street 80-233 Gdańsk Poland

**Keywords:** amino acids, asymmetric synthesis, antifungal agents, antibacterial agents, antiprotozoal agents

## Abstract

Structures of several dozen of known antibacterial, antifungal or antiprotozoal agents are based on the amino acid scaffold. In most of them, the amino acid skeleton is of a crucial importance for their antimicrobial activity, since very often they are structural analogs of amino acid intermediates of different microbial biosynthetic pathways. Particularly, some aminophosphonate or aminoboronate analogs of protein amino acids are effective enzyme inhibitors, as structural mimics of tetrahedral transition state intermediates. Synthesis of amino acid antimicrobials is a particular challenge, especially in terms of the need for enantioselective methods, including the asymmetric synthesis. All these issues are addressed in this review, summing up the current state‐of‐the‐art and presenting perspectives fur further progress.

## Graphical abstract

**Structures of several dozen** known antibacterial, antifungal or antiprotozoal agents are based on an amino acid scaffold. In most of them, the amino acid skeleton is crucial for antimicrobial activity, as very often they are structural analogs of amino acid intermediates of various microbial biosynthetic pathways. Synthesis of amino acid antimicrobials is a particular challenge, especially in terms of the need for enantioselective methods, including asymmetric synthesis.

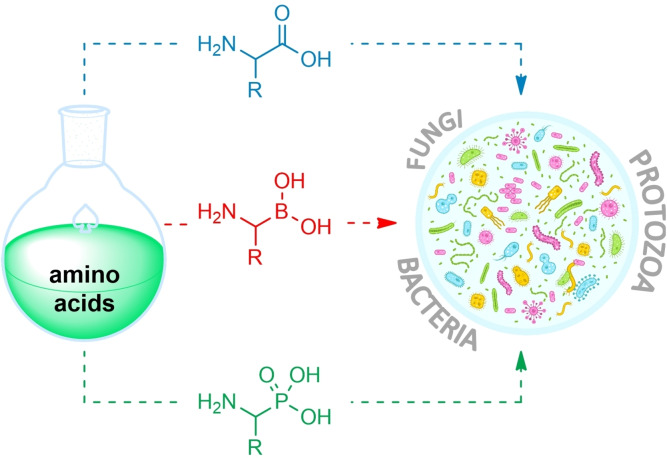

## 1 Introduction

Since the discovery of penicillin, antimicrobial chemotherapy has been one of the prime topics in medicine. To date, several hundreds of antimicrobial agents have been discovered, isolated from natural sources or designed and synthesized. Nevertheless, much less than 1 % of them have become drugs. With the overuse of antimicrobials, emerging microbial drug resistance and appearance ‘super bugs,’ there is a general agreement that the ‘golden era’ of antibiotics is long gone.^[1]^

Bacterial infections are considered to be the most difficult to treat and to design efficient antibiotic agents, due to problems like poor penetration into pathogenic cells and fast development of bacterial resistance.[^1^, ^2^] The highest priority in antibacterial drug research and development was assigned by WHO to agents active against *Acinetobacter*, *Pseudomonas* and carbapenem‐ resistant species of *Enterobacterales*.^[3]^

Fungal infections, especially deadly disseminated mycoses caused by human pathogenic yeasts and filamentous fungi in immunocompromised patients, constitute one of the crucial challenges for modern chemotherapy.^[4]^ In the recent years, the situation is getting even worse, due to the emerging fungal drug resistance and an appearance of novel fungal pathogens, including a superbug *Candida auris*.^[5]^ A current repertoire of antifungal chemotherapeutics is very limited and some of them are constantly losing their antifungal efficacy, so that there is an urgent need for novel potential antifungal drug candidates.

*Plasmodium* spp. are protozoans causing malaria, the most frequent infectious disease in global scale. On the other hand, *Trypanosoma* spp. are responsible for African sleeping sickness. Although a number of antiprotozoal drugs are available, protozoal resistance to them is emerging, so that any novel drugs are desirable.

Searching for new antimicrobial agents in the current period, called sometimes a “post‐antibiotic era,” has been intensified in recent years. Especially valuable could be compounds with novel molecular targets. Obviously, although diversity of chemical structures of known antimicrobial drugs is unquestionable, many of them are antimetabolites, i. e. structural analogs of intermediates of microbial metabolism, especially biosynthesis. In this respect, non‐protein amino acids of natural^[6]^ or synthetic origin and their derivatives constitute an important group of drugs or drug candidates. Particularly, such appropriately designed compounds can be strong inhibitors of enzymes involved in metabolism of protein amino acids, but there is also a surprisingly large number of enzymes participating in other microbial biochemical pathways, which are confirmed or awaiting validation as molecular targets for amino acid antimicrobials.

Protein amino acids but also a number of non‐protein amino acids are synthesized with a handful of well known‐methods like Gabriel^[7]^ or Strecker synthesis.^[8]^ However, these standard methods cannot be applied, especially if the target molecule is a non‐carboxylic amino acid. Meanwhile, many phosphonic or boronic acid‐based amino acids can act as transition state analog inhibitors of several target enzymes.[^9^, ^10^] Chirality of most biologically active forms of amino acids additionally makes their synthesis hard and forces scientists to utilize asymmetric synthesis strategies or calls for often inefficient resolution of racemic mixtures.

In this review, we present examples of amino acids and amino acid‐derived compounds of natural or synthetic origin, known as inhibitors of some enzymes present in human pathogenic bacteria, fungi or protozoa and synthetic strategies of their preparation.

## 2 Antibacterial amino acid‐based agents

### 2.1 Molecular targets for amino acid‐based antibacterials

**Scheme 1:**
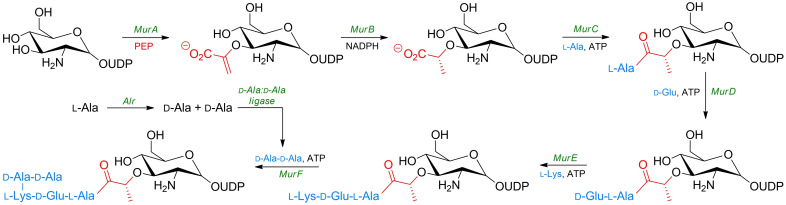
Simplified synthesis of murein.

Most of the molecular targets for amino acid‐based antibacterials are enzymes involved in biosynthesis of peptidoglycan (murein), a principal component of the bacterial cell wall. They catalyze particular steps in formation of oligopeptide substituents of *N*‐acetylmuramic acid residues, the so‐called MurA−F pathway (Scheme 1). Kinetics, specificity and catalytic mechanism of these enzymes were thoroughly reviewed.[^11^, ^12^, ^13^, ^14^] Particularly, a few excellent reviews that cover the topic of low‐molecular‐weight inhibitors of Mur enzymes have been published recently.[^15^, ^16^, ^17^]

Presence of essential non‐protein amino acid residues in the peptide part of peptidoglycan, especially d‐alanine, d‐glutamic acid and d‐glutamine, absent in mammalian cells, makes the enzymes involved in their formation and further processing, attractive targets for antibacterial agents.

#### 2.1.1 Alanine racemase

Alanine racemase (Alr) is a prokaryotic enzyme belonging to the family of isomerases. This enzyme catalyses conversion of l‐alanine into its d‐enantiomer, thus providing an essential substrate for the formation of the oligopeptide part of peptidoglycan. Alr is a pyridoxal phosphate dependent enzyme and this cofactor is responsible for the formation of a Schiff base with the amino group of alanine, thus giving rise to the facilitated abstraction of its α proton and subsequent stabilization of the carbanion intermediate thus formed. Absence of this enzyme in eukaryotes makes it an attractive target for antibacterial drug design for both Gram‐positive and Gram‐negative bacteria. These features also makes bacterial alanine racemase one of the best studied amino acid racemases.[^18^, ^19^] Several interesting reviews on alanine racemase and its inhibitors are available.[^19^, ^20^, ^21^, ^22^]

#### 2.1.2 d‐Ala : d‐Ala ligase

d‐Ala : d‐Ala ligase (ADP forming) is an enzyme that catalyzes the formation of a d‐Ala : d‐Ala dipeptide that occupies the C‐terminal position in the cytosolic cell wall (UDP)‐*N*‐acetylmuramyl pentapeptide precursor unit of peptidoglycan. d‐Ala : d‐Ala ligase uses two d‐Ala molecules as substrates, so that d‐Ala analogs are potential inhibitors of this enzyme.^[23]^

#### 2.1.3 β‐lactamase

β‐Lactamases are bacterial enzymes that are ancient in their origin but are nowadays abundant in many bacterial species, due to the overuse of generations of β‐lactam antibiotics in the last century.^[24]^ Bacteria possessing β‐lactamase are resistant to penicillin G, penicillin V, ampicillin, amoxicillin and some other β‐lactam antibiotics of first generation. Since β‐lactam antibiotics have been the backbone of antibacterial chemotherapy, a growing frequency of appearance of the β‐lactamase‐containing bacteria is a substantial challenge. The effect of β‐lactams on those antibiotics ranges from slight to complete inhibition of antibiotic activity of an otherwise active inhibitor and depends on the quantity of enzyme in target pathogen.^[25]^

One of the strategies of combating infections caused by β‐lactamase possessing bacteria is their treatment with a combination of a β‐lactam antibiotic and inhibitor of β‐lactamase.

### 2.2 Inhibitors of MurA‐F enzymes incorporating α‐amino carboxylic acids

#### 2.2.1 Serine derivatives

**Figure 1:**
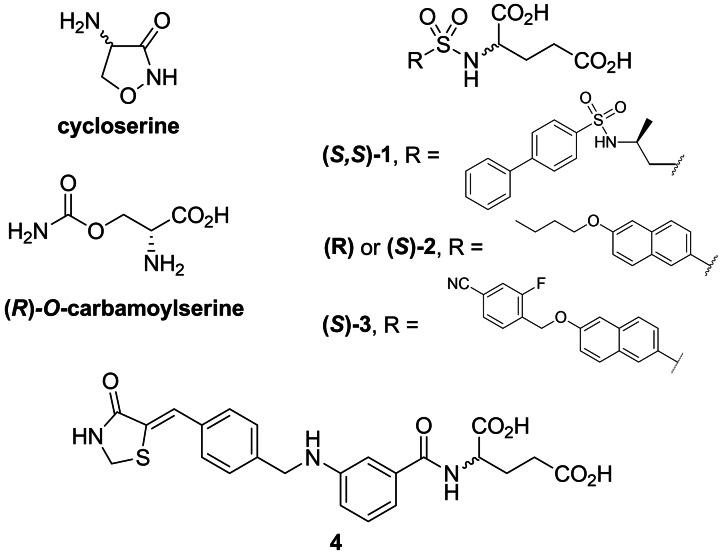
Inhibitors of bacterial enzymes incorporating proteinogenic amino acids: d‐cycloserine, (*R*)‐*O*‐carbamoylserine; derivatives of glutamic acid **1–4**

One of the first discovered amino acid‐based structures with antibacterial properties was serine derivatives. The inhibitory effect of d‐cycloserine (originally known under names orientomycin^[26]^ or oxamycin^[27]^ (Figure 1) on *Staphylococcus aureus* was reported by Strominger *et al*. in 1960.^[28]^ In that and further studies,^[29]^ they proposed that this activity was due to ability of d‐cycloserine to act as an inhibitor of Alr and d‐Ala : d‐Ala ligase. Inhibitory effect on the former enzyme was confirmed by Lambert *et al*. in 1972.^[30]^ Interestingly, they found out that l‐cycloserine is also an effective competitive inhibitor of this enzyme. Originally, d‐cycloserine was obtained by isolation from several species of *Streptomyces*.^[31]^ After cycloserine was proposed as a broad spectrum antibiotic in the 1950s, several reports of its synthesis had been presented.[^32^, ^33^, ^34^] Utility of d‐cycloserine as an antibacterial drug is limited because it is also a co‐agonist of NMDA receptor in the brain.^[35]^ Clinical use of d‐cycloserine is restricted to the treatment of tuberculosis as a second‐line drug for MDR strains of *Mycobacterium tuberculosis*.^[36]^ There is an ambiguity over the precise lethal target (alanine racemase or d‐Ala : d‐Ala ligase) in *M. tuberculosis*.^[37]^

Another early discovered antibacterial serine derivative was *O*‐carbamoyl‐d‐serine (Figure 1). It was originally isolated from a strain of *Streptomyces* by Hageman *et al*. in 1955.^[38]^ The antibiotic activity of this compound on *B. subtilis* was reversed by d‐alanine, what indicated that it acts as a d‐alanine antagonist.^[39]^ The mechanism of action of this antibiotic was explained by Lynch *et al*.^[40]^

Stammer *et al*. synthesized dl‐cycloserine using the cyclization reaction of dl‐β‐aminoxyalanine ethyl ester, followed by resolution of diastereoisomeric tartarates by crystallization,^[32]^ whereas Plattner *et al*. synthesized both d‐ and l‐cycloserine by cyclization of a corresponding α‐amino‐β‐chlorohydroxamic acids.^[33]^

**Scheme 2:**
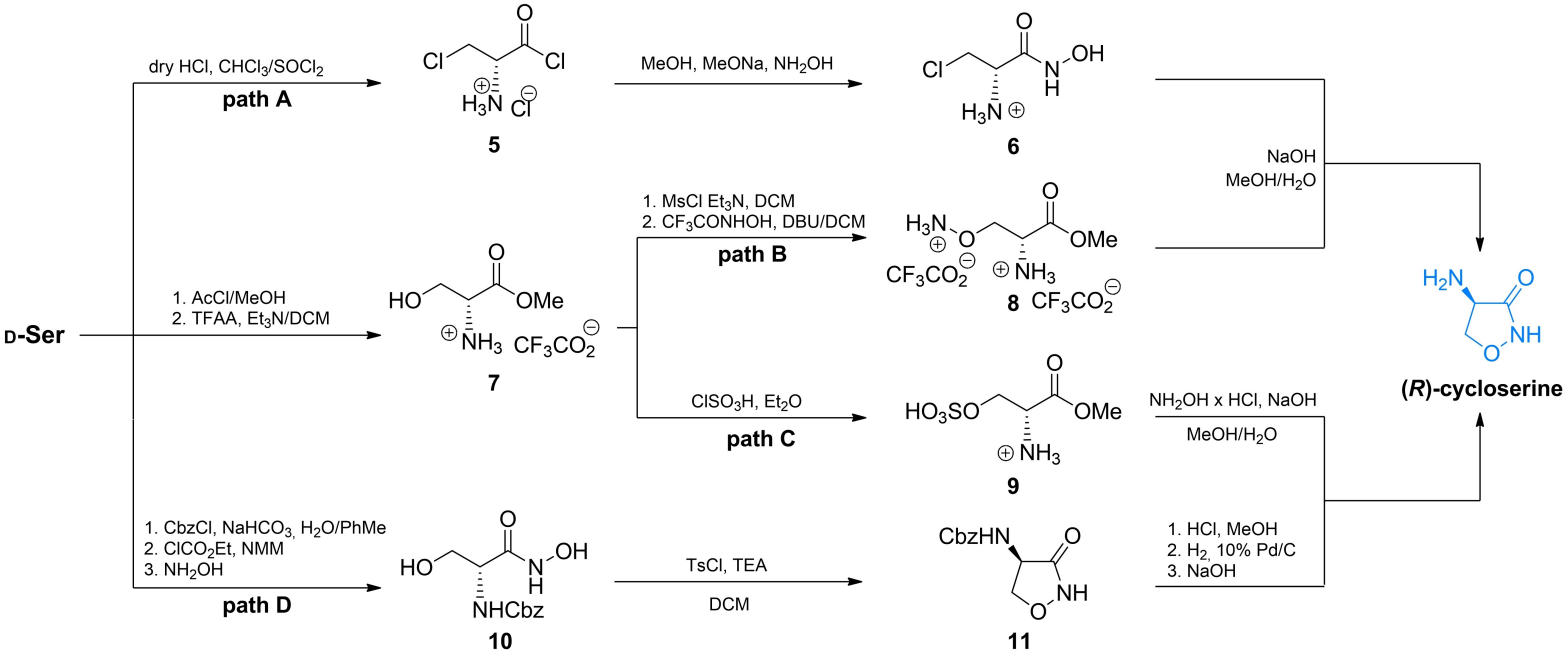
Synthesis of d‐cycloserine from d‐serine.

Several more modern approaches for the synthesis of enantiomerically pure cycloserines were reported in the 2010s.[^41^, ^42^, ^43^, ^44^, ^45^, ^46^, ^47^] Li *et al*. proposed an original and efficient synthesis of d‐cycloserine (Scheme 2, path A). In that approach, they transformed d‐serine into the acid chloride of β‐chloroalanine **5** and then into to hydroxamic acid **6** which readily undergoes cyclization reaction to d‐cycloserine.^[41]^

Kim *et al*. proposed another simple method of d‐cycloserine synthesis in which the target molecule was obtained *via* transformation of d‐serine methyl ester **7** to ω‐aminoxy derivative **8**, which readily forms an isoxazolidine ring (Scheme 2, path B).[^43^, ^44^] The same team designed another method of d‐cycloserine synthesis.^[43]^ In that study ester **7** was transformed into sulfate **9**. Subsequently, this intermediate was treated with hydroxylamine, leading to the target compound (Scheme 2, path C). Awasthi *et al*. reported synthesis of this compound with remarkable purity, under mild reaction conditions.^[47]^ In that approach they used *N*‐benzyloxycarbonyl‐protected d‐serine and transformed it into *N*‐protected derivative **11** *via* cyclization reaction of ω‐hydroxyhydroxamic acid **10** under basic conditions (Scheme 2, path D).

**Scheme 3:**
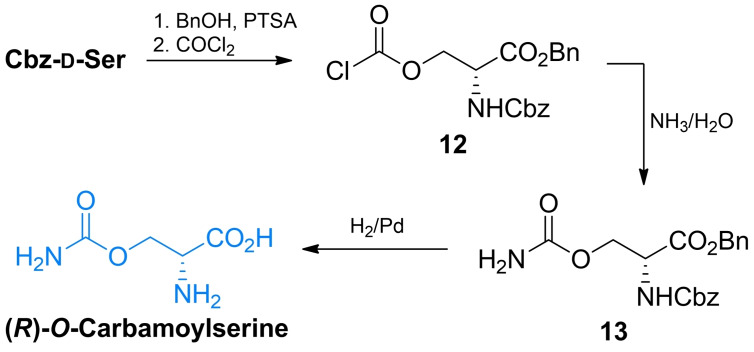
Synthesis of *O*‐carbamoyl‐d‐serine.

The synthesis of (*R*)‐carbamoylserine was reported by Skinner *et al*.^[48]^ The authors used *N*‐benzyloxycarbonyl‐d‐serine and transformed it into the benzyl ester which was subsequently transformed into chloroformate **14** through phosgene treatment. This chloroformate was quenched with aqueous ammonia to yield the protected carbamate **15**. One step deprotection of both amino and carboxyl functional groups *via* palladium‐catalyzed hydrogenation led to the final compound (Scheme 3).

#### 2.2.2 Glutamic acid derivatives

Humljan *et al*. were working on improved inhibitors of MurD using sulfonamide as a functional group that could be used as a tetrahedral transition‐state mimetic. This idea gave birth to the group of amino acid‐based inhibitors which consist of sulfonamides of glutamic acids. While most of the synthesized compounds did not exhibit satisfactory inhibitory activity on MurD enzyme from *E. coli*, one of them ‐ **1** (Figure 1), exhibited good inhibitory activity on MurE from *E. coli*.^[49]^ In further studies, the same team synthesized derivatives of naphthalene‐*N*‐sulfonyl‐d‐Glu that led to the structure of both enantiomers of compound **2**.^[50]^ Both enantiomers inhibited MurD from *E. coli* although the activity of derivative (*S*)‐**2** was significantly lower. Further optimization of these naphthalene structures resulted in new inhibitors. In that group compound **3** turned out to be the most potent inhibitor.^[51]^ Another type of substituted glutamic acid are benzylidene‐2,4‐thiazolidin‐diones. One of the first potent inhibitors designed and synthesized by Tomašić *et al*. was compound **4**.^[52]^ Interestingly, in the case of this compound, no significant reduction of activity against *E. coli* MurD was observed for l‐Glu derivative when compared to d‐derivative.

**Scheme 4:**
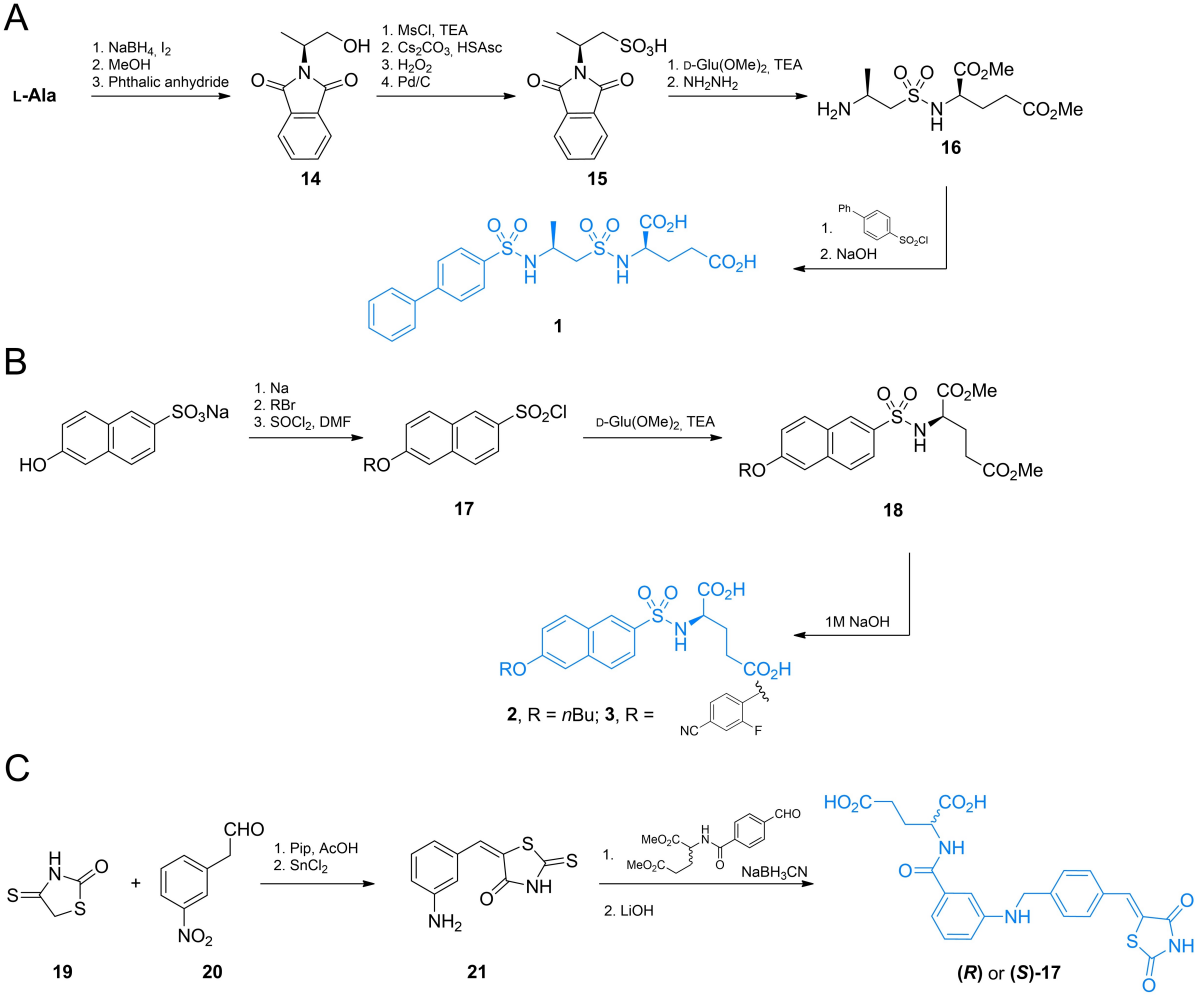
Synthesis of glutamic acid‐based β‐lactamase inhibitors.

Compound **1** was synthesized by Humljan *et al*. using l‐alanine as a starting material (Scheme 4A).^[49]^ After reduction of the carboxylic group and protection of the amino function with phthalimide system, a hydroxyl group of **14** was converted into sulfhydryl and *S*‐oxidised into sulfonic acid **15**. Subsequently, compound **15** was converted into sulfuryl chloride and conjugated with d‐glutamic acid dimethyl ester and phthalimide function with hydrazine. The formed amine **16** was conjugated with [1,1’‐biphenyl]‐4‐sulfonyl chloride and finally treated with aqueous sodium hydroxide, to yield compound **1**.

Humljan *et al*. also designed a route of synthesis of both stereoisomers of **2** and **3** (Scheme 4B).^[51]^ In this case sodium 6‐hydroxynaphthalene‐2‐sulfonate was converted to a corresponding aryl‐alkyl ether *via* simple ether formation from phenolate and alkyl bromide. The formed ether was then converted into arylsulfonylchloride **17** which was conjugated with d‐glutamic acid dimethyl ester. Treatment of **18** with NaOH led to the compound **2**. Compound **3** was prepared using the same method.

Both enantiomers of **4** were synthesized by Tomašić *et al*. (Scheme 4C).^[52]^ The authors used the aldol‐type condensation reaction of 2‐thioxo‐1,3‐thiazolidin‐4‐one **19** and (*m*‐nitrophenyl)acetaldehyde **20**. The formed nitrobenzene derivative was subsequently reduced to aniline‐derivative **21** and coupled with dimethyl ester of *N*‐(4‐formylbenzoyl)‐d‐glutamic acid *via* reductive amination reaction, followed by methyl ester hydrolysis yielded compound **4**.

### 2.3 Inhibitors of MurA‐F enzymes based on β,γ‐unsaturated amino acids

**Figure 2:**
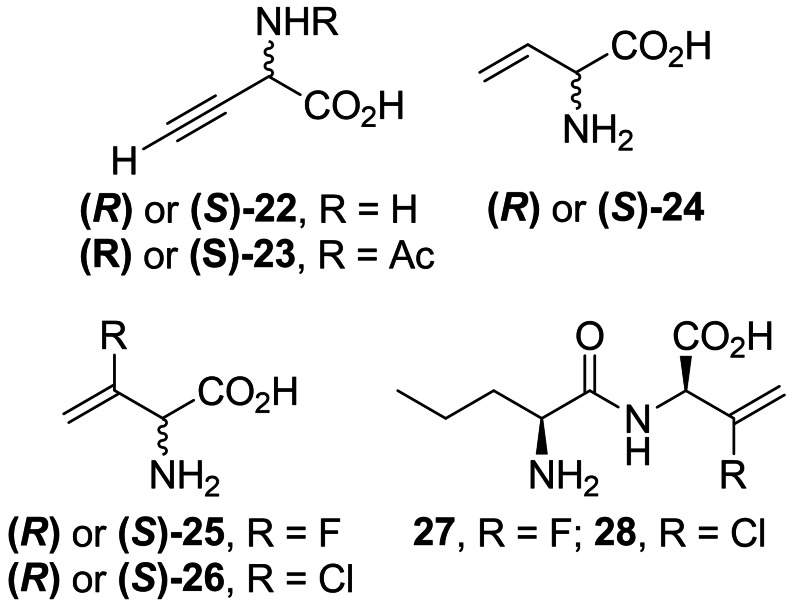
Inhibitors **22–24** of bacterial enzymes incorporating β,γ‐unsaturated amino acids.

While β,γ‐unsaturated amino acids are rare in Nature, some of them turned out to be effective alanine racemase inhibitors. l‐α‐Ethynyl glycine (**22**, Figure 2) is the simplest of β,γ‐unsaturated amino acid exhibiting activity against Gram‐positive bacteria^[53]^ due to the inhibition of Alr. It was proposed that **22** covalently binds to alanine racemase after the incorporation of PLP‐phosphate.^[54]^ **22** was isolated from the fungus *Streptomyces catenulae* and found to be very labile in alkaline solution. On the other hand, its *N*‐acetyl derivative (**23**, Figure 2) is stable and retains antimicrobial activity.^[55]^

**Scheme 5:**
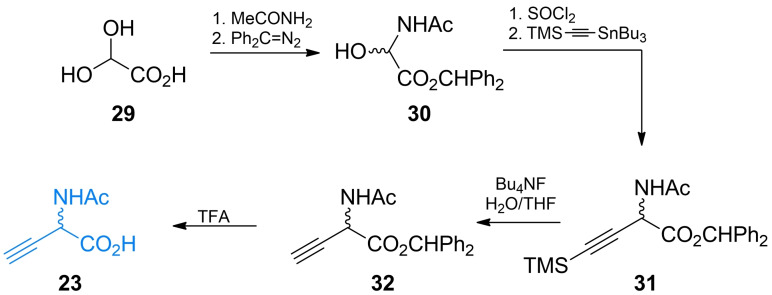
Synthesis of ethynylglycine.

Shortly after β,γ‐alkenyl and β,γ‐alkynyl amino acids received more attention after it became apparent that introducing an unsaturated carbon‐carbon bond in the strategic position of known enzyme substrates provides additional levels of constraint that may lead to the increased inhibitory activity on the enzyme. Synthetic chemistry of β,γ‐alkynyl α‐amino acids was thoroughly reviewed by Meffre and Le Goffic^[53]^ and method of preparation of racemic α‐ethynyl glycine was developed by Williams *et al*. (Scheme 5).[^56^, ^57^] The authors used 2,2‐dihydroacetic acid **29** as a starting material. The amino acid system was created *via* substitution with acetamide and formation of diphenylmethyl ester with diphenyldiazomethane. The formed α‐hydroxyamino acid derivative **30** was treated with thionyl chloride and subsequently reacted with trimethylsilylethynyl tributylstannane yielding a functional group‐protected compound **31**. The terminal ethynyl group of **32** was created by the treatment of **31** with tetrabutylammonium fluoride. Subsequent TFA treatment of **32** led to the racemic **23** which could be further transformed into labile **22** *via* the chemoenzymatic process utilizing rat kidney acylase.

Thornberry *et al*. reported 3‐halovinylglycines as a new class of potent irreversible inactivators of Alr from *E. coli*.^[58]^ In further studies both enantiomers of 3‐fluorovinylglycine and 3‐chlorovinylglycine (**25** and **26**, Figure 2) proved to be mechanism‐based inhibitors of Alr, although fluoro derivatives turned out to be much less reactive than chlorovinylglycines.^[59]^ At the same time, detailed synthesis of those compounds as racemic mixtures were reported.^[60]^ The same team also synthesized and evaluated antibacterial activities of norvalyl dipeptides of those amino acids and among those compounds l‐norvalyl‐l‐3‐fluorovinylglycine **27** and its chloro analog **28** displayed the best inhibitory activity against Gram‐negative bacteria.^[61]^

**Scheme 6:**
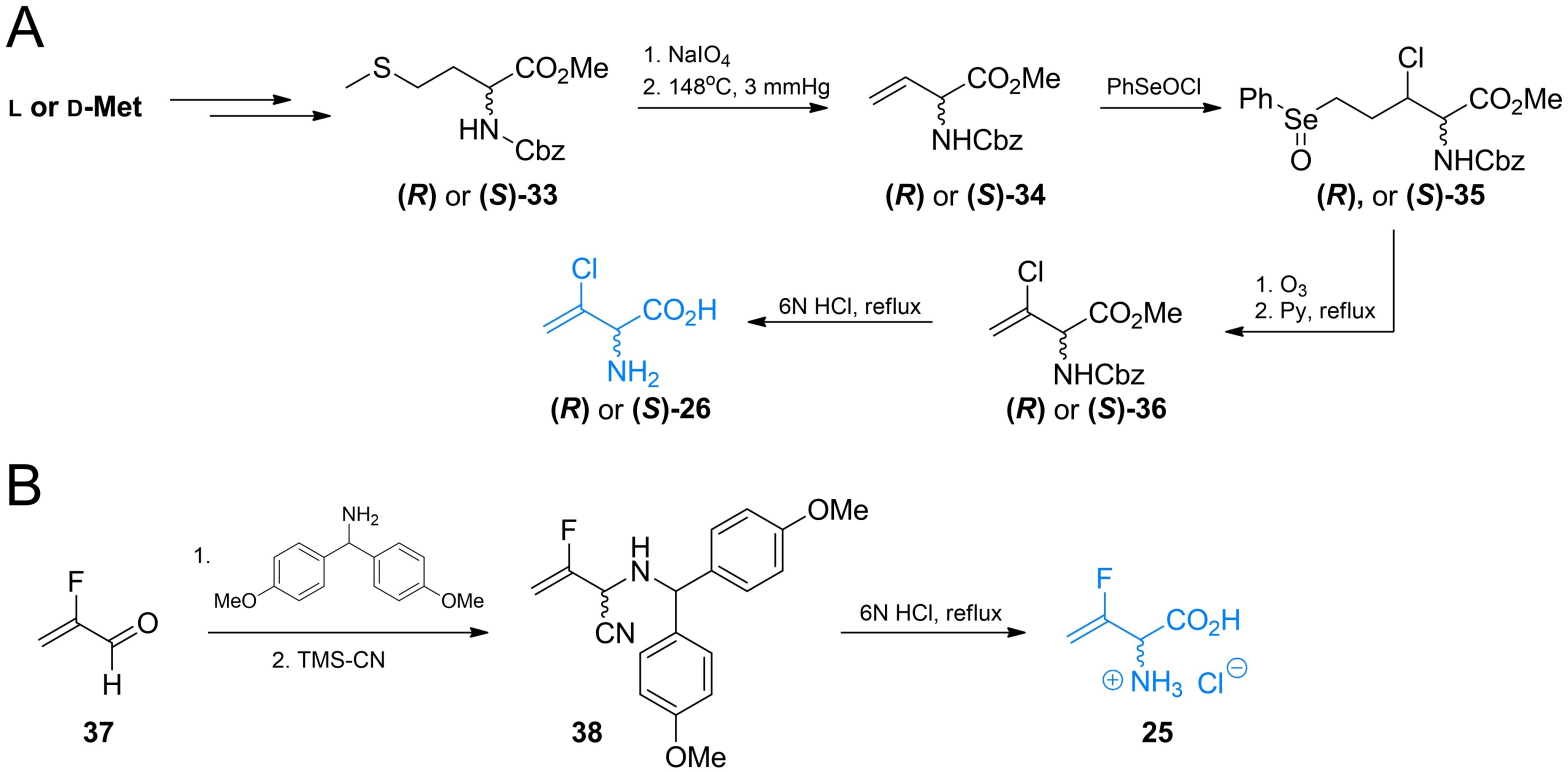
Synthesis of α‐halovinyl amino acids.

In the synthesis of **26**, functional group‐protected d‐ or l‐methionine (Scheme 6A) was used as a starting material. Synthesis of those compounds from enantiomers of methionine was previously reported by Afzali‐Ardakani *et al*.^[62]^ **33** was oxidized to sulfoxide and heated to undergo sulfoxide elimination, yielding an unsaturated compound **34**. Next, **34** was treated with benzeneselenic chloride to add the 3‐chloro substituent. The formed compound **35** was then heated in presence of pyridine, which led to selenoxide elimination reaction and formation of 3‐chlorovinyl system (**36**). Treatment of **36** with 6 M HCl yields a corresponding enantiomer of **26**.

Racemic 3‐fluorovinylglycine **25** was prepared from 2‐fluoroacrolein **37** *via* the modified Strecker synthesis (Scheme 6B). The imine obtained from bis(4‐methoxyphenyl)methanamine and TMS‐CN was used to introduce the nitryl function giving compound **38**. Hydrolysis of this compound in 6 M HCl formed racemic inhibitor **25** in its racemic form. Norvalyl peptides of halovinylglicines **27** and **28** were obtained from **25** and **26** respectively using standard NSU‐promoted amide bond formation with amino function of Nva.^[61]^

### 2.4 Inhibitors of Alr based on β‐halogenated amino acids

**Figure 3:**
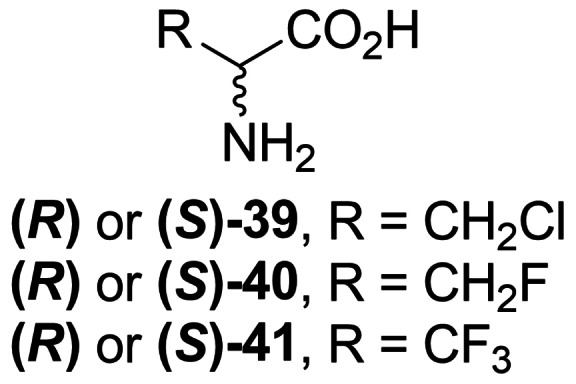
β‐Haloalanines **39–41** exhibiting antibacterial activity.

Manning *et al*. first reported the inhibitory activity of l‐ and d‐isomers of β‐chloroalanine (**39**, Figure 3) activity on several bacterial alanine racemases.^[63]^ Other β‐halogenated alanine derivatives – β‐l‐fluoroalanine and β‐d‐fluoroalanine (**40**, Figure 3) were reported as effective inhibitors of *S. typhymurium* Alr. It was suggested that those compounds are suicide substrates for Alr, as aforementioned chloro‐ analogs.^[64]^

Polyfluorinated alanine derivative β,β,β‐trifluoroalane (**41**, Figure 3) is a known inhibitor of PLP‐dependent enzymes, including Alr.^[65]^ Mechanism of inactivation of alanine racemase from *Bacillus stearothermophilus* and *S. typhymurium* was revealed in 1989.^[66]^

**Scheme 7:**
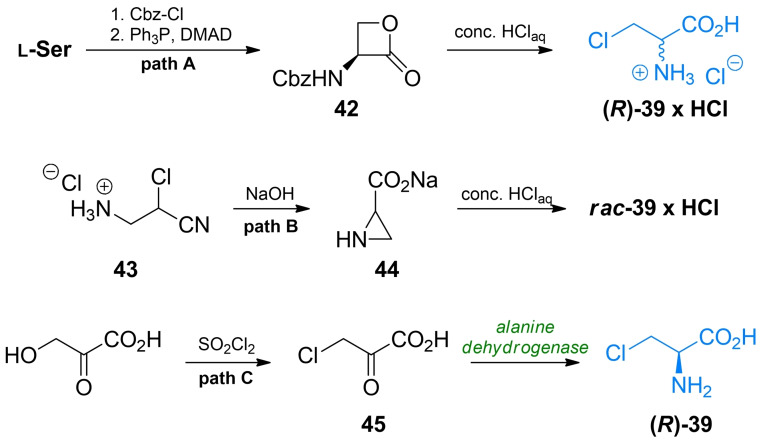
Synthesis of β‐chloroalanines.

Synthesis of β‐chloroalanine **39** was known for a long time, mainly due to its use as an intermediate in organic synthesis. In those applications enantiomers of β‐chloroalanine are usually synthesized from a corresponding enantiomers of serine in a simple nucleophilic substitution of ‐OH by ‐Cl, commonly with thionyl chloride as reagent).[^67^, ^68^, ^69^] Arnold *et al*. presented the synthesis of optically pure β‐chloroalanines using ring‐opening reaction of α‐amino‐β‐propiolactone **42** with excellent yields (Scheme 7, path A).[^70^, ^71^] The cost‐effective method of racemic β‐chloroalanine preparation was proposed for industrial applications.[^72^, ^73^] In that method 3‐amino‐2‐chloropropane nitrile **43** is converted into aziridine‐2‐carboxylate **44**, which can be subsequently transformed into racemic β‐chloroalanine with aqueous hydrochloric acid (Scheme 7, path B). Chemoenzymatic synthesis of β‐l‐chloroalanine from 3‐chloropyruvic **47** acid using amino acid dehydrogenase was reported (Scheme 7, path C).^[74]^

**Scheme 8:**
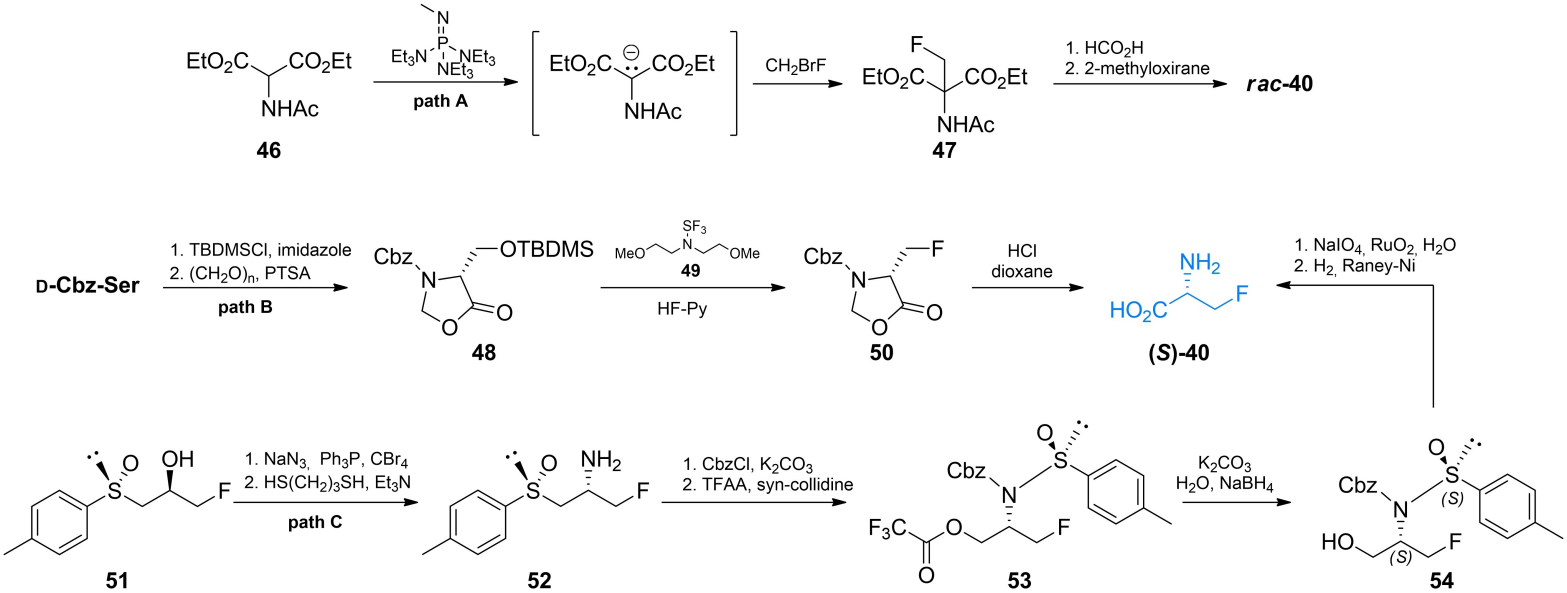
Synthesis of β‐fluoroalanines.

Fluoroalanine **40** was originally obtained by Kollonitsch and Barash *via* photofluorination reactions of l‐ and d‐alanine.^[75]^ Gerus *et al*. synthesized racemic β‐fluoroalanine **40** *via* addition of fluoromethylene group into aminomalonic acid derivative **46** (Scheme 8, path A).^[76]^ The formed aminomalonate **47** was subsequently treated with formic acid and propylene oxide to give racemic **40**. Synthesis of optically pure enantiomers of β‐fluoroalanine was reported by Hoveyda and Pinault (Scheme 8, path B).^[77]^ *N*‐Protected serine was treated with *tert*‐butyldimethylsilyl chloride and paraformaldehyde to form oxazolidinone derivative **48**. A fluorine atom was introduced in appropriate position using reagent **49**. The formed fluorinated compound **50** could be then directly hydrolyzed in HCl/dioxane or deprotected in a three‐step process.

β‐d‐Fluoroalanine was synthesized by Bravo *et al. via*'chiral sulfoxide’ chemistry (Scheme 8, path C).^[78]^ The authors used optically pure compound **51** and transformed its hydroxyl group into amino groups by the means of S_N_2 reaction with controlled inversion of configuration (**52**). Protection of the amino function with (benzyloxy)carbonyl group followed by the treatment with trifluoroacetic acid anhydride led to the transfer of sulfoxide moiety to form sulfinamide **53**. Reduction of TFA ester to alcohol **54** followed by oxidation and Cbz group cleavage yielded (*S*)‐**40** with high optical purity. In both cases, excellent enantiomeric excess was achieved. Synthesis of *N*‐Fmoc‐l‐fluoroalanine accomplished by Carpentier *et al*. is worth mentioning, as it enables use of β‐fluoroalanines as building blocks in solid phase peptide synthesis and possibly other synthetic strategies.^[79]^

β,β,β‐Trifluoroalanine (or simply trifluoroalanine), like its monofluoro as well as monochloro analogs happened to be an inhibitor of bacterial Alr^[80]^ but working by different inactivation mechanism than monohalogated alanines.^[66]^

**Scheme 9:**
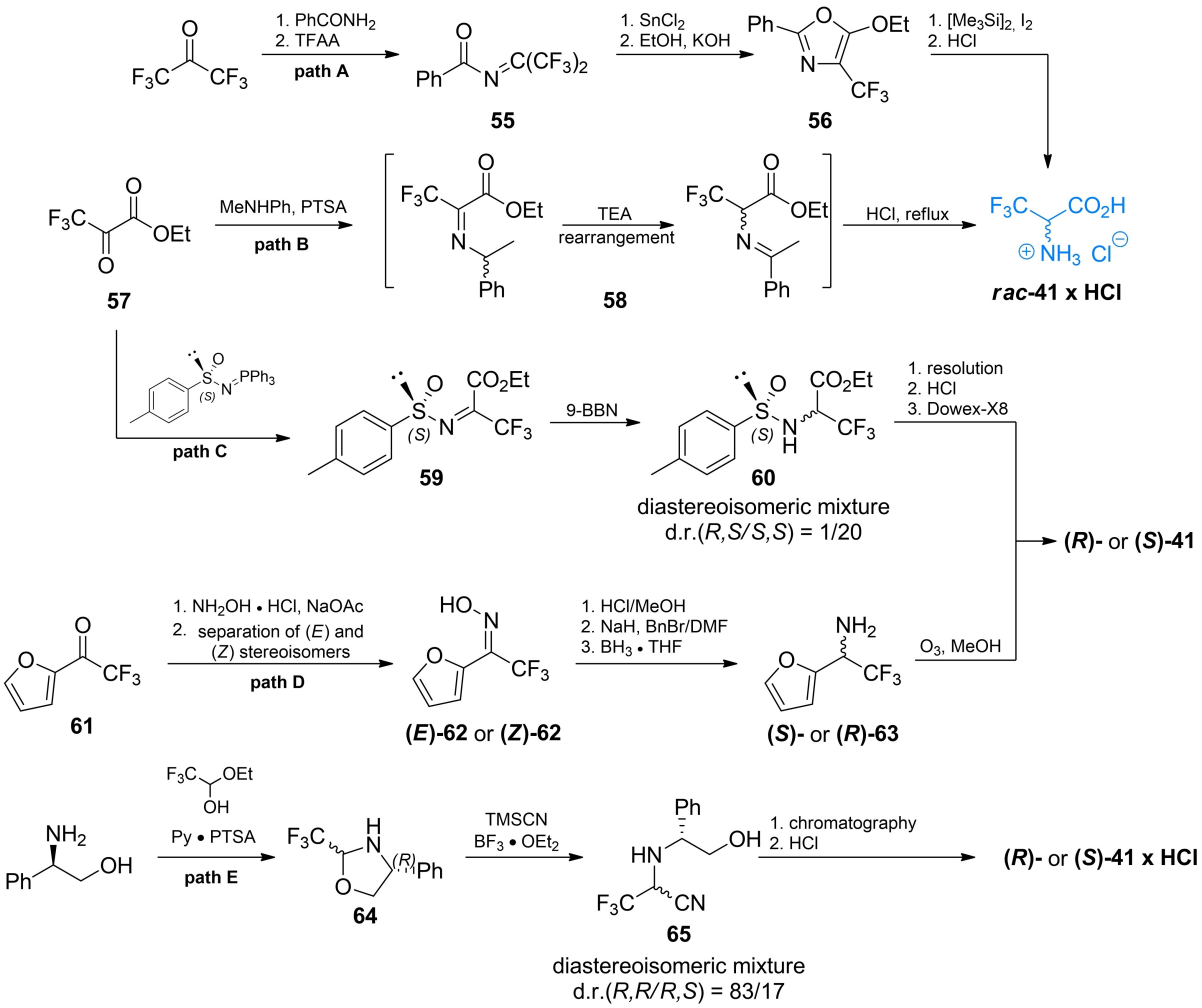
Synthesis of trifluoroalanine.

Burger *et al*. proposed one of the first early synthesis of racemic trifluoroalanine from hexafluoroacetone (Scheme 9, path A).[^81^, ^82^] The authors used hexafluoroacetone‐derived (perfluoroporpan‐2‐ylidene)benzamide **55** which undergoes tin(II)‐promoted cyclization to oxazole‐ring. The formed compound **56** may be easily transformed into racemic trifluoroalanine with *in situ* obtained TMS−I.

Soloshonok *et al*. used ethyl trifluoropyruvate **57** as a starting material to synthesize racemic trifluoroalanine (Scheme 9, path B). Imine formed with methylphenylamine undergoes [1,3]‐proton shift reaction that leads to rearrangement of this compound to imine **58** which can be hydrolyzed in HCl to give racemic trifluoroalanine.^[83]^ Synthesis of non‐racemic trifluoroalanine was proposed by Crucianelli *et al*. (Scheme 9, path C).^[84]^ In that approach the authors used chiral sulfinamide **59** to form diastereoisomeric intermediate compounds **60** that can be easily separated and subsequently hydrolyzed to yield non‐racemic trifluoroalanines.

Diastereoisomeric oximes of (1‐furyl)‐trifluoromethyl ketone **62** were used by Demir *et al*. as the strategy to obtain both enantiomers of trifluoroalanine (Scheme 9, path D).^[85]^ Ketone **61** was transformed into diastereoisomeric mixture of oximes which was easily separated *via* column chromatography. Oximes **62** were then treated with hydrochloric acid and reduced with BF_3_×THF complex to yield enantiomerically pure amines **63**. The final ozonolysis of furan rings leads to a corresponding enantiomers of **41**.

Lebouvier *et al*. proposed stereoselective synthesis of trifluoroalanine *via* ring‐opening reaction of Lewis‐acid activated 1,3‐oxazolidine derivative^[86]^ (Scheme 9, path E). Oxazolidine derivative **64** in diastereoisomeric mixture was obtained *via* condensation of (*R*)‐2‐amino‐2‐phenyl‐1‐ethanol and 1‐ethoxy‐2,2,2‐trifluoro‐1‐ethanol. Ring opening reaction with TMSCN was subsequently performed to obtain compound **65** with high diastereoisomeric excess towards the *R,R* stereoisomer. Resolution of diasteroisomers of **65**, followed by acid hydrolysis of a corresponding isomers, led to the final formation of enantiomers of **41**.

### 2.5 Inhibitors of MurA‐F enzymes based on aminophosphonic acids

#### 2.5.1 Non‐halogenated alanine analogs

**Figure 4:**
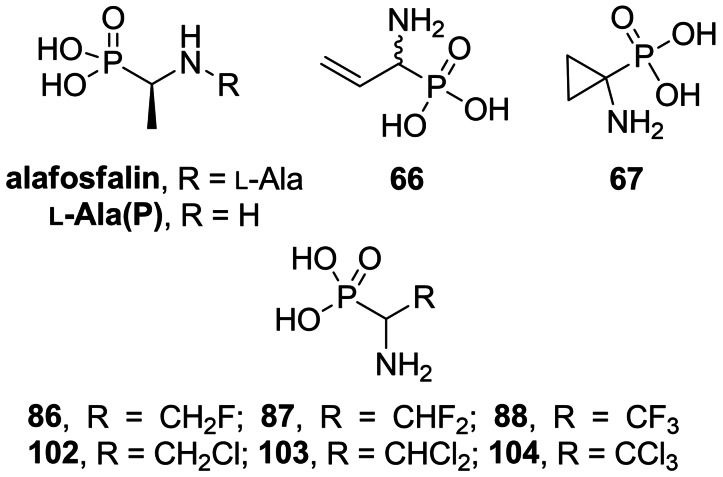
Inhibitors of bacterial enzymes based on 1‐aminophosphonic acid: phosphonoalanine, vinyl derivative **66**, cyclopropane derivative **67**, β‐haloalanine derivatives **86–88** and **102–104**.

In 1979, Atheron *et al*. discovered antibacterial peptide mimetic l‐alanyl‐l‐1‐aminoethylphosphonic acid which was later named alafosfalin (Figure 4).^[87]^ Alafosfalin was found to be hydrolyzed to 1‐aminoethyl phosphonic acid (**l‐Ala(P)**, Figure 4) and this product was identified as an irreversible Alr inhibitor.^[88]^ There are several reports on both racemic and asymmetric synthesis of alafosfalin.[^89^, ^90^, ^91^, ^92^] Vo‐Quang *et al*. synthesized (1‐amino‐2‐propenyl)phosphonic acid (**66**, Figure 4).^[93]^ The authors reported inhibitory properties of this amino acid on alanine racemases and d‐Ala‐d‐Ala ligases from *P. aeruginosa* and *S. faecalis*. 1‐Aminocyclopropanephosphonic acid (**67**, Figure 4) was reported to be an inhibitor of 1‐aminocyclopropanecarboxylate deaminase from *Pseudomonas sp*. and alanine racemase from *Bacillus stearothermophilus*.^[94]^

**Scheme 10:**
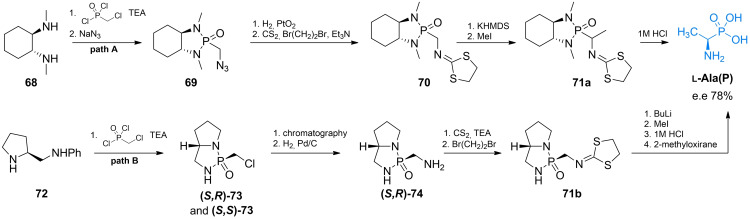
Synthesis of **l‐Ala(P)**.

The asymmetric synthesis of α‐amino‐α‐alkylphosphonic acids with high enantiomeric purity was proposed by Hanessian *et al*. (Scheme 10, path A).^[95]^ The authors proposed usage of a diastereoisometrically pure cyclohexylamine derivative to form the chiral cyclic amide of (chloromethyl)phosphonic acid **69** and subsequently introduced the protected amino group through iminomethyldithiolane formation. The formed compound **70** has an acidic α‐proton which can be substituted with an alkyl group in a highly stereoselective manner. Methyl iodide was chosen to alkylate pseudo‐enolate, thus forming compound **71 a**, which can be treated with 1 M HCl solution to yield **l‐Ala(P)** with high enantiomeric excess. Another example of asymmetric synthesis of **l‐Ala(P)** was presented by Yuan *et al*.^[96]^ (*S*)‐2‐Anilinomethylpyrrolidine **72** was used as a chiral auxiliary (Scheme 10, path B). After separation of diastereoisomers **73**, optically pure compound **71 b** was transformed into **l‐Ala(P)** in a similar way. Asymmetric synthesis of the second enantiomer (**d‐Ala(P)**)^[97]^ and successful resolution of racemic form of Ala(P) were also reported.^[98]^

**Scheme 11:**
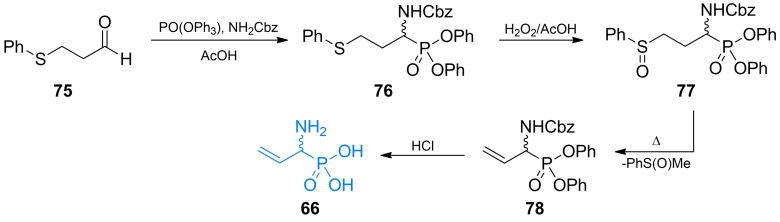
Synthesis of α‐aminophosphonic acid‐based antibacterial **66**.

Vo‐Quang *et al*. synthesized (1‐amino‐2‐propenyl)phosphonic acid (**66**, Figure 4)^[93]^ in its racemic form using 3‐(phenylthio)propyl aldehyde as a starting material (Scheme 11). α‐Amino alkylphosphonic system was synthesized by one‐step reaction of **75** with both amino‐ and phosphonic group donor. The formed compound **76** was subsequently oxidized to sulfoxide **77. 77** undergoes phenylmethylsulfoxide elimination reaction while being heated. The formed phosphonic acid **78** can be easily deprotected under acidic conditions yielding racemic **66**.

**Scheme 12:**
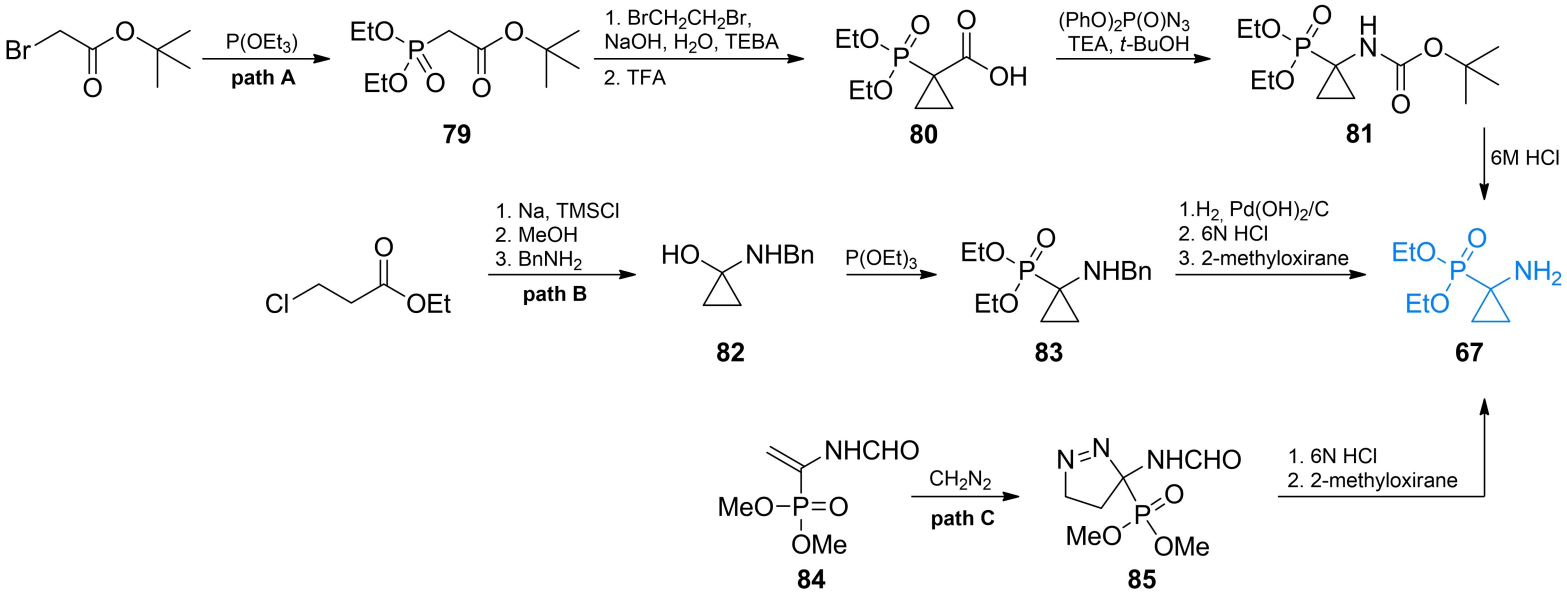
Synthesis of α‐aminophosphonic acid‐based antibacterial **67**.

Erion *et al*. obtained **67** *via* alkylation reaction of triethyl phosphite with *tert*‐butyl bromoacetate (Scheme 12, path A).^[94]^ The formed α‐phosphonoester **79** undergoes malonate‐like alkylation which enables a synthesis of cyclopropane moiety using 1,2‐dibromethane. The formed compound **80** was then converted to an amide **81** by Curtius rearrangement using diphenylphosphoryl azide. Protected amino acid **81** was subsequently hydrolyzed with hydrochloric acid yielding racemic form of **67**.

Fadel *et al*. synthesized **67** from ethyl 3‐chloropropionate which underwent cyclization reaction in a presence of metallic sodium to yield cyclopropanone hemiacetal (Scheme 12, path B).^[99]^ The hemiacetal was then coupled with benzylamine to form compound **82**. Subsequently, it was treated with triethyl phosphite to give **83**, which yielded **67** after consecutive deprotection reactions. Goulioukina *et al*. synthesized 1‐aminocyclopropanephosphonic acid **67** using 1,3‐dipolar cycloaddition to unsaturated phosphonate **84**, obtaining in effect a pyrazoline derivative **85** (Scheme 12, path C).^[100]^ Ester **85** undergoes molecular nitrogen elimination in acidic aqueous condition thus yielding 1‐aminocyclopropanephosphonic acid.

#### 2.5.2 Fluoroalanine analogs

**Scheme 13:**
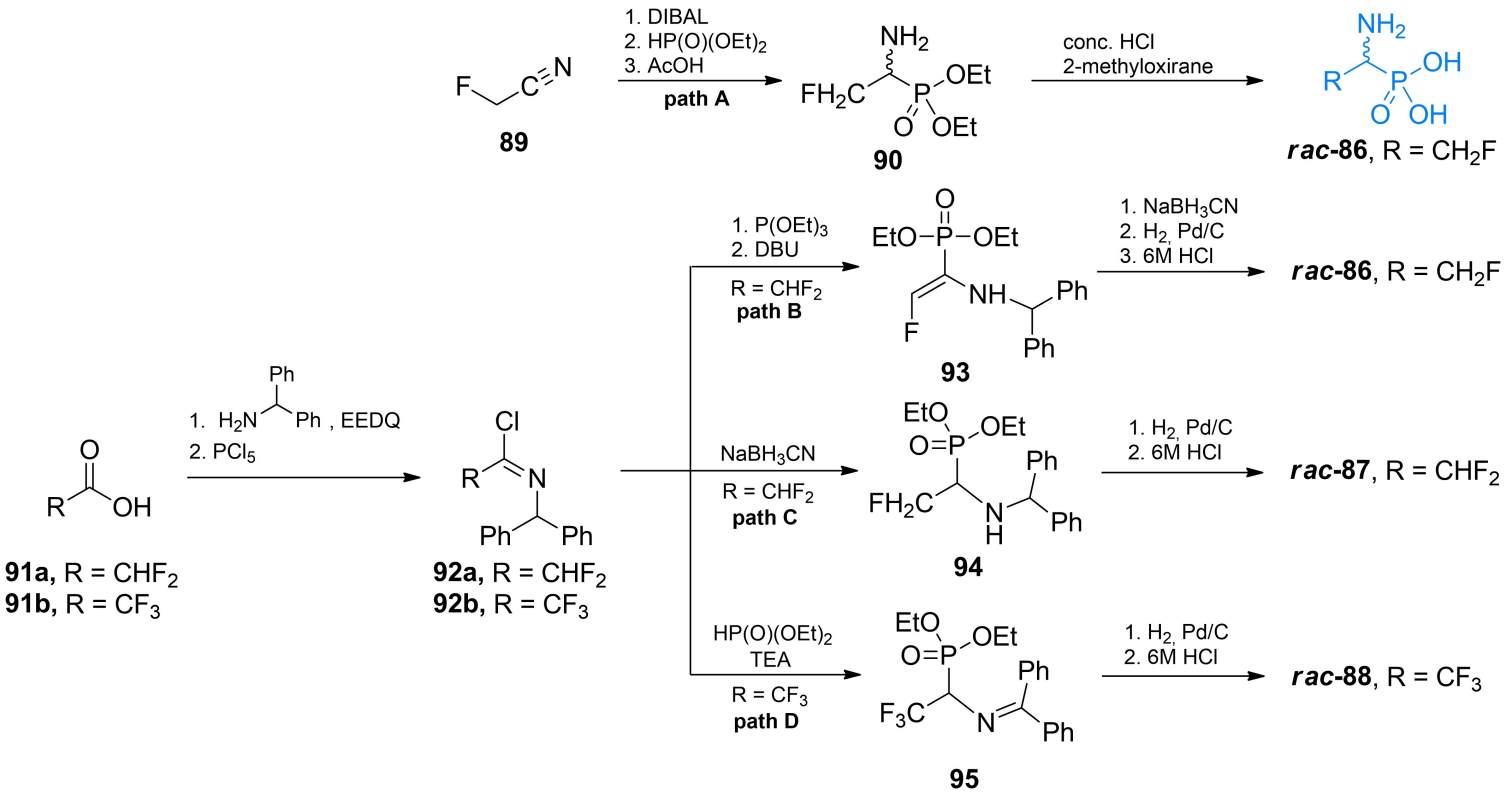
Synthesis of racemic fluorinated α‐aminophosphonic acid‐containing antibacterials.

Kudzin *et al* synthesized racemic β‐fluoro‐1‐aminoethanephosphonic acid (**86**, Figure 4) using fluoroacetonitrile **89** as a starting material (Scheme 13, path A).^[101]^ The nitryl group of **89** was reduced with DIBAL and transformed into imine derivative. Addition of diethyl phosphonite to this imine followed by hydrolysis of the formed **90** led to the formation of β‐trifluoro‐β‐aminoethanephosphonic acid **86**.

Flynn *et al*. proposed another way of preparation of racemic β‐fluoro‐1‐aminoethanephosphonic acid **86** as well as its difluoro **87** and trifluoro analogs **88** (Scheme 13, paths B–D).^[102]^ These compounds can be prepared starting either with difluoroacetic or trifluoroacetic acid and forming amide with diphenylmethylamine utilizing EEDQ strategy and subsequently converting it to imidoyl chloride **92**.

To obtain monofluoro derivative the authors used imidoyl chloride **92 a** (path B) and reacted it with triethyl phosphite and then DBU which leads to eventual elimination of HF molecule and formation of enamine **93**. Reduction and subsequent deprotection of **93** yielded fluoro‐1‐aminoethanephosphonic acid **86**. Difluoro derivative (path C), was synthesized using imidoyl chloride **92 a** and treated with triethyl phosphite and DBU, followed by reduction of the formed imine to benzyl‐type amine **94**. This intermediate was subsequently hydrogenated and hydrolyzed in 6 M HCl, to yield **87**. To synthesize 2,2,2‐trifluoro‐1‐aminoethanephosphonic acid (path D) appropriate imidoyl chloride **92 b** is reacted with diethyl phosphonite in presence of organic base. The formed imine **95** is then cleaved by hydrogenation and acidified to obtain **88**.

**Scheme 14:**
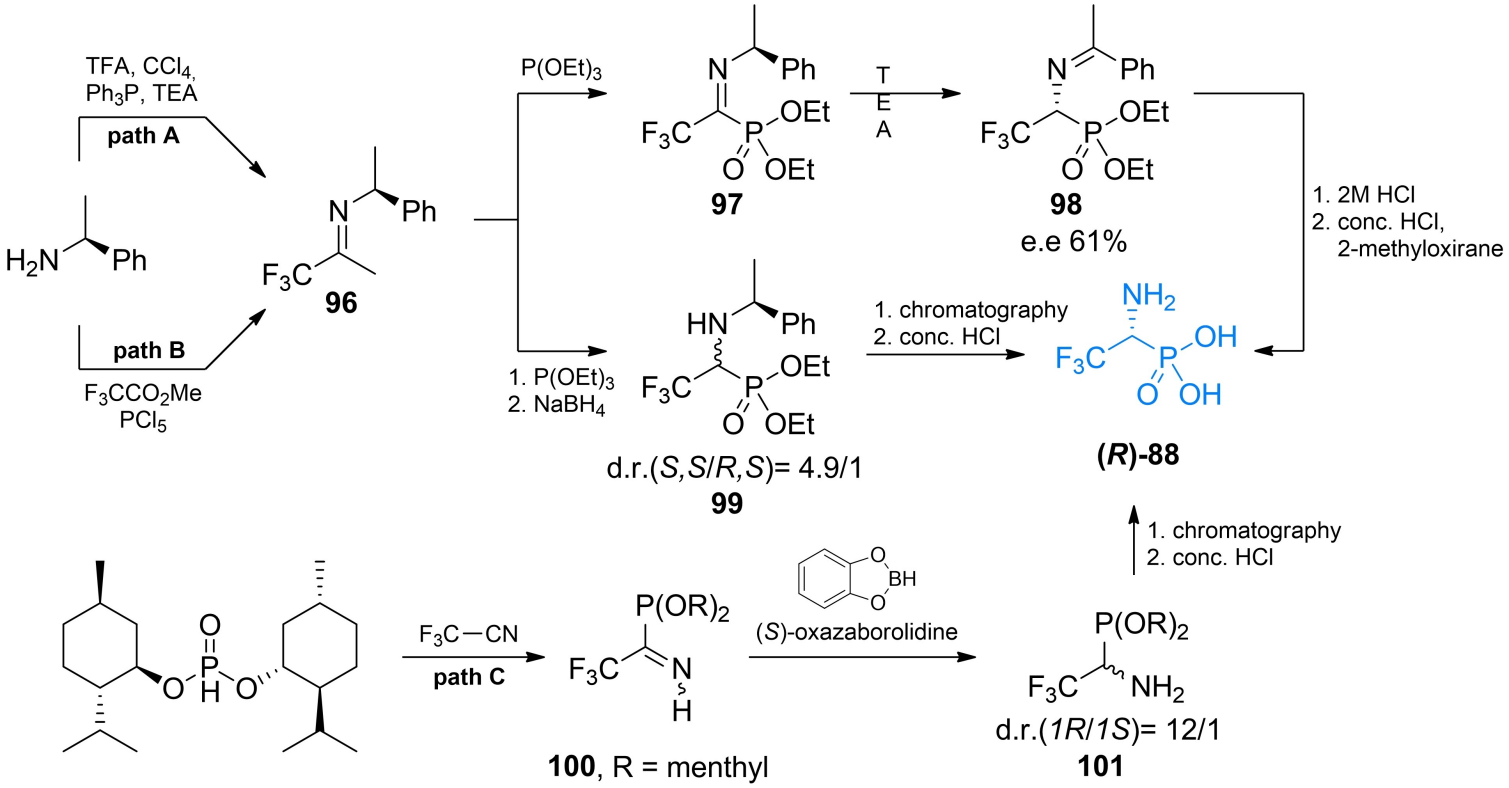
Asymmetric synthesis of racemic fluorinated α‐aminophosphonic acid‐containg antibacterials.

In 2000 Xiao *et al*. proposed facile asymmetric synthesis of **88** (Scheme 14, path A).^[103]^ In that approach (*S*)‐phenylethylamine was used to form chiral imidoyl chloride **96** with trifluoroacetic acid. Next imidoyl chloride **96** was reacted with triethyl phosphite to introduce a phosphonic group. The formed imine **97** after treatment with an organic base underwent [1,3]‐proton shift, reaction which yielded imine **98** which was subsequently treated with aqueous hydrochloric acid yields (*R*)‐**88** with reasonable enantiomeric excess.

Stanko and Rassukana proposed two other methods of obtaining non‐racemic 2,2,2‐trifluoro‐1‐aminoethanephosphonic acid **88** (Scheme 14, paths B and C).[^104^, ^105^] In one of these methods, the authors used an approach similar to that of Xiao *et al*., to obtain chiral imidoyl chloride **96**. After reaction of **96** with triethyl phosphite the authors performed reduction with sodium borohydride, which led to the mixture of diastereoisomers **99** with significant diastereoisomeric excess, in favor for (*S,S*) configuration. After separation of diastereoisomers and treatment with concentrated hydrochloric acid the authors managed to obtain both enantiomers of 2,2,2‐trifluoro‐1‐aminoethanephosphonic acid **88**. In another approach the same authors used trifluoroacetonitrile as starting material and (+)‐menthol was used as a chiral auxiliary. (+)‐Dimenthyl ester of phosphonic acid reacted with trifluoroacetonitrile to form imine **100**. Reduction of this amine with benzodioxoborole in the presence of (*S*)‐oxazaborolidine led to diastereoisomeric mixture of 2,2,2‐trifluoro‐1‐aminoethanephosphonic acid (+)‐dimethyl ester **101** with high diastereoisomeric excess. Appropriate enantiomer of **101** can be easily separated and hydrolyzed to obtain either enantiomer of **88**.

#### 2.5.3 Chloroalanine analogs

Vo‐Quang *et al*. synthesized phosphonic analogues of β‐chloroalanines (**102**–**104**, Figure 4). Although mono and dichloro showed strong affinity for the alanine racemase, all three derivatives were proven not to be the suicide inhibitors of this enzyme.^[106]^

**Scheme 15:**
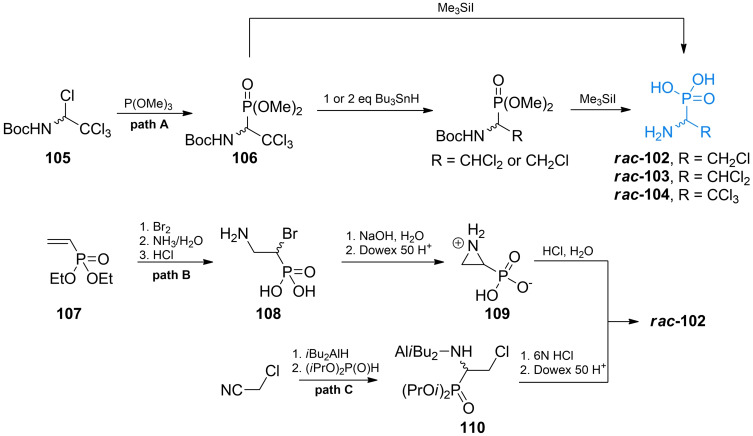
Synthesis of chlorinated α‐aminophosphonic acid‐containg antibacterials.

In their synthetic approach Vo‐Quang *et al*. used *N*‐*t*‐butoxycarbonyl‐1,2,2,2‐tetrachloroamine **105** that reacted with trimethyl phosphite to form compound **106**. The phosphonic ester **106** was used as a precursor for the synthesis of all three chloro‐Ala(P) derivatives (Scheme 15, path A). The authors used iodotrimethylsilane to oxidize and hydrolyze the phosphonic ester and obtained chloro‐Ala(P) derivative. The trichloro derivative was obtained by directly treating aforementioned intermediate with TMSI. Dichloro and monochloro derivative were obtained by using appropriate amounts tributylstannane and treating of the formed compound with TMSI.

Monochloro derivative **102** was also prepared by Zygmunt *et al*. using diethyl vinylphosphonate **107** as starting material.[^107^, ^108^] The precursor was then transformed into 1‐bromo‐2‐amino derivative **108** by treating it consecutively with bromine, aqueous ammonia and aqueous HCl. Upon treating with 1 M NaOH, **108** undergoes intramolecular substitution which leads to the formation of aziridine‐2‐phosphonic acid **109**. This cyclic compound was treated with aqueous hydrochloric acid to yield racemic **102** (Scheme 15, path B).

Recently, Qian *et al*. proposed a novel method to obtain **102** from chloroacetonitrile *via* reduction with DIBAL. The formed imine was subsequently treated with diisopropyl phosphonate to form target compounds scaffold **110** and then with 6 M hydrochloric acid to yield racemic **102** (Scheme 15, path C).^[109]^

#### 2.5.4 Pseudodipeptides incorporating phosphonic and phosphinic acid residues

**Figure 5:**
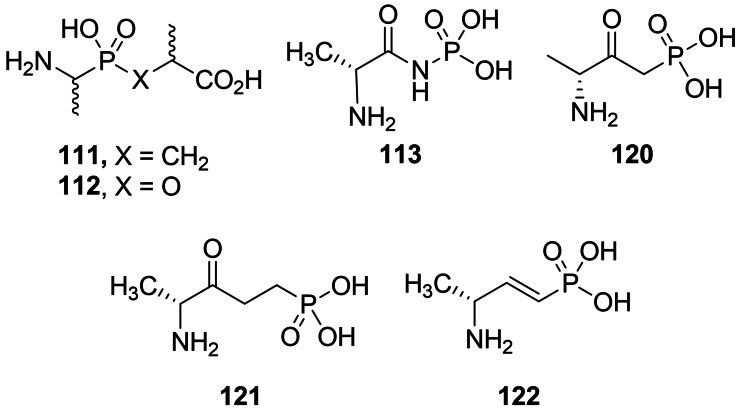
Pseudodipeptides incorporating phosphonic and phosphinic acid residues: alkylphosphinic acid **111**, alkylphosphonate **112**, phosphoramidic acid **113** and alkylphosphonic acids **120–122**.

Parsons *et al*. synthesized series of phosphonic acid dipeptides with a structure mimicking a transition state of reaction catalyzed by d‐Ala : d‐Ala ligase. The most effective of the series was compound (*S,R*)‐**111**, was found to be a potent and irreversible inhibitor of d‐Ala : d‐Ala ligase and demonstrated antibacterial activity.^[110]^ In 1987, Chakravarty *et al*. synthesized another series of phosphonic acid‐based pseudopeptides based on the structure of d‐alanyl‐d‐alanine.[^111^, ^112^] Among those pseudodipeptides, **113** was found to be a more potent inhibitor of the enzyme than d‐cycloserine although it did not show any significant activity on bacteria. Representative structures are shown in Figure 5. Synthetic route used by Parsons *et al*.^[110]^ was based on the work s of Baylis *et al*.^[113]^ Those authors used *rac*‐*N*‐benzyloxycarbonyl‐(1‐aminoethyl)phosphonic acid **114** as starting materials and performed Michael addition of this compound to methyl methacrylate. Subsequent deprotection reaction yielded final racemic product **111**.

**Scheme 16:**
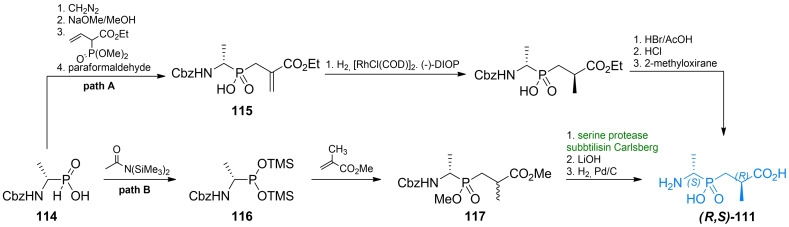
Synthesis of antibacterial pseudodipeptide **(*R,S*)‐111**.

Asymmetric synthesis of compound **111** was achieved using enantiomerically pure *N*‐benzyloxycarbonyl‐(1‐aminoethyl)phosphinic acid **114** (Scheme 16, path A). Stereochemical control of the second asymmetric center was achieved by introducing a double bond in the structure of the pseudopeptide *via* Michael addition to trimethylphosphonoacrylate and subsequent reaction with formaldehyde. The formed acrylate derivative **115** was reduced in stereoselective way using hydrogen in the presence of [RhCl(COD)]_2_ and (−)‐DIOP under high pressure what yielded a corresponding diastereoisomer of **111**. Several other pseudodipeptides were also synthesized using this route.^[110]^

Ellsworth *et al*. synthesized non‐racemic **111** using (*S*)‐*N*‐benzyloxycarbonyl‐(1‐aminoethyl)phosphinic acid **114** (Scheme 16, path B).^[114]^ The authors converted this compound to alkylphosphonous acid derivative **116** by treating it with bis(trimethylsilyl)acetamide. Compound **116** was then used as P‐nucleophile in Michael addition to methyl methacrylate. The formed protected pseudodipeptide in racemic mixture **117** was resolved by digestion with the serine protease subtilisin (Carlsberg), and treated with LiOH and then with hydrogen on Pd/C, to obtain desired optically pure compounds.

**Scheme 17:**
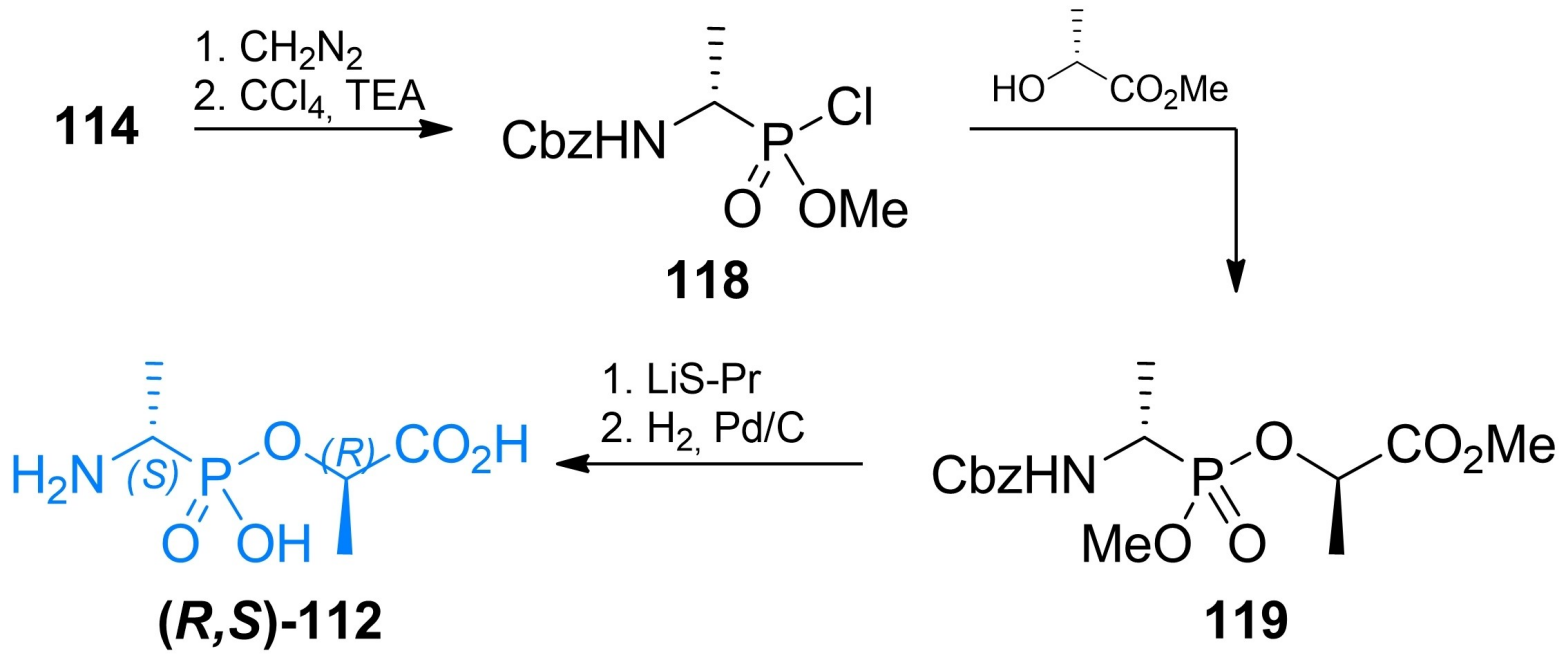
Synthesis of antibacterial pseudodipeptide **(*R,S*)‐112**.

Pseudodipeptides based on alkylphosphonic acids **112** were synthesized by Ellsworth *et al*. (Scheme 17).^[114]^ Similarly to the previous authors’ approach to synthesis of compound **111**, **114** was used as a starting material and transformed into methyl alkylphosphonochloridite **118**. The formed compound could be conveniently conjugated with optically pure l‐lactic acid, thus forming the pseudopeptide backbone. Cleavage of protecting groups resulted in enantiomerically pure compound **112**. Jia *et al*.^[115]^ proposed a total synthesis of racemic **112** from triphenylphosphite, benzyl carbamate and acetaldehyde.

**Scheme 18:**
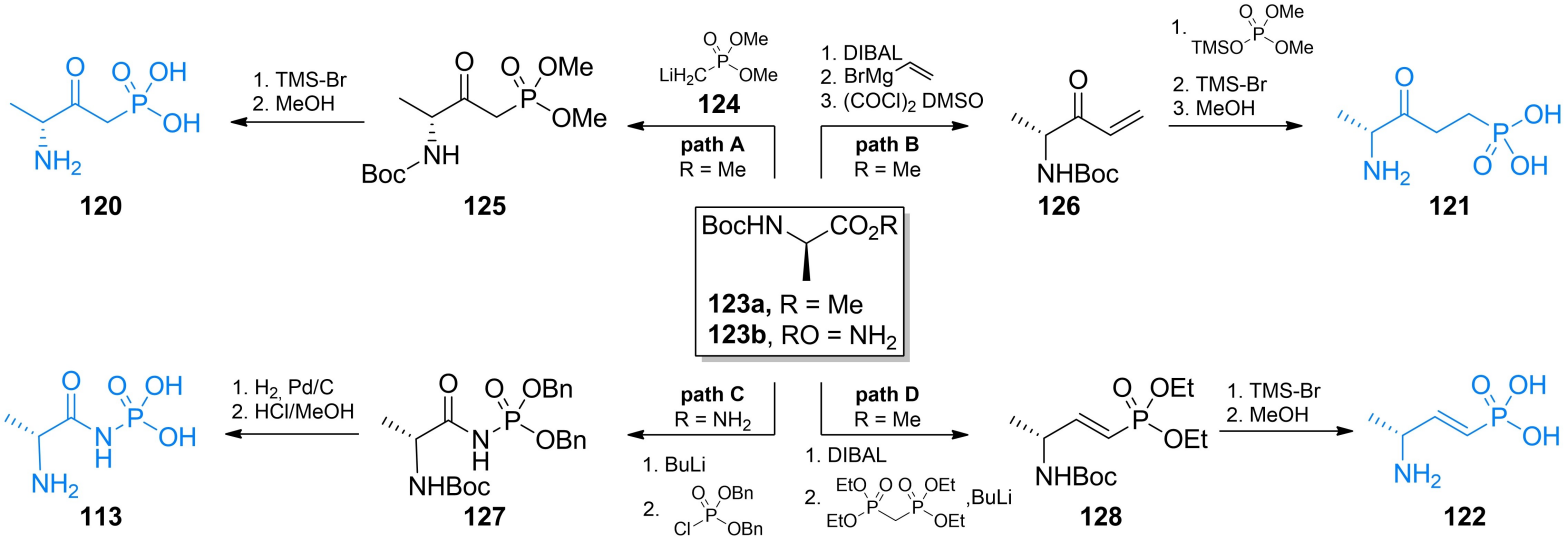
Synthesis of antibacterial pseudodipeptides **113** and **120–122**.

Two ω‐amino‐β‐oxoalkylphosphonic acids were synthesized by the same group. Compound **120** (Figure 5) was synthesized *via* the Claisen‐like condensation of *N*‐*tert*‐butoxycarbonylamino acid methyl ester **123 a** and methyl ester of alkylphosphonic acid **124**. The formed compound **125** was treated with trimethylsilyl bromide and subsequently with methanol to yield compound **120** (Scheme 18, path A). Compound **121** was synthesized *via* the addition of vinylmagnesium bromide to **123 a** yielding α,β‐unsaturated ketone **126**. Phosphonic function was introduced upon Michael addition of dimethyl (trimethylsilyl)phosphate and finally, deprotection reactions were performed to yield **121** (Scheme 18, path B). Phosphonamide derivative **113** was synthesized *via* deprotonation of *N*‐*tert*‐butoxycarbonyl‐d‐alanine amide **123 b** and *N*‐phosphorylation with dibenzyl phosphorochloridate to yield intermediate **127**. After series of deprotection reactions **113** was obtained. (Scheme 18, path C). Unsaturated phosphonic acid was synthesized *via* DIBAL reduction of **123 a** followed by olefination under Horner‐Emmons conditions. Unsaturated phosphonate **128** was treated with trimethylsilyl bromide and methanol to yield compound **122** (Scheme 18, path D).

### 2.6 Inhibitors of Alr incorporating α‐amino boronic acids

**Figure 6:**
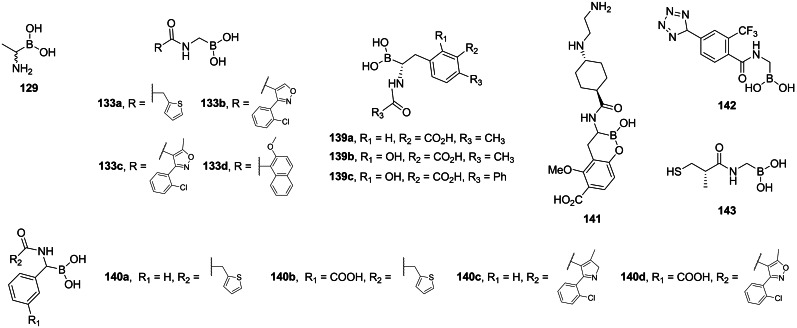
Aminoboronic acid‐based antibacterials: boronic analog of alanine analog **129**, boronic analogs of *N*‐acylglycines **133 a**–**d**, **142** and **143**, boronic analogs of *N*‐acylphenylalanines **139 a**–**c** and **141**, boronic analogs of *N*‐acetylphenylglycines **140 a**–**d**.

In 1989 Duncan *et al*. reported inhibitory activity of boronic acid alanine analog (**129**, Figure 6) on *B. stearothermophilus* alanine racemase and *S. typhimurium* d‐Ala : d‐Ala ligase.^[116]^ Caselli *et al*. designed many antibacterial inhibitors based on α‐aminoboronic acid structure.[^117^, ^118^, ^119^] Several inhibitors of β‐lactamase were designed and synthesized by Martin and *et al*.[^120^, ^121^, ^122^]

**Scheme 19:**
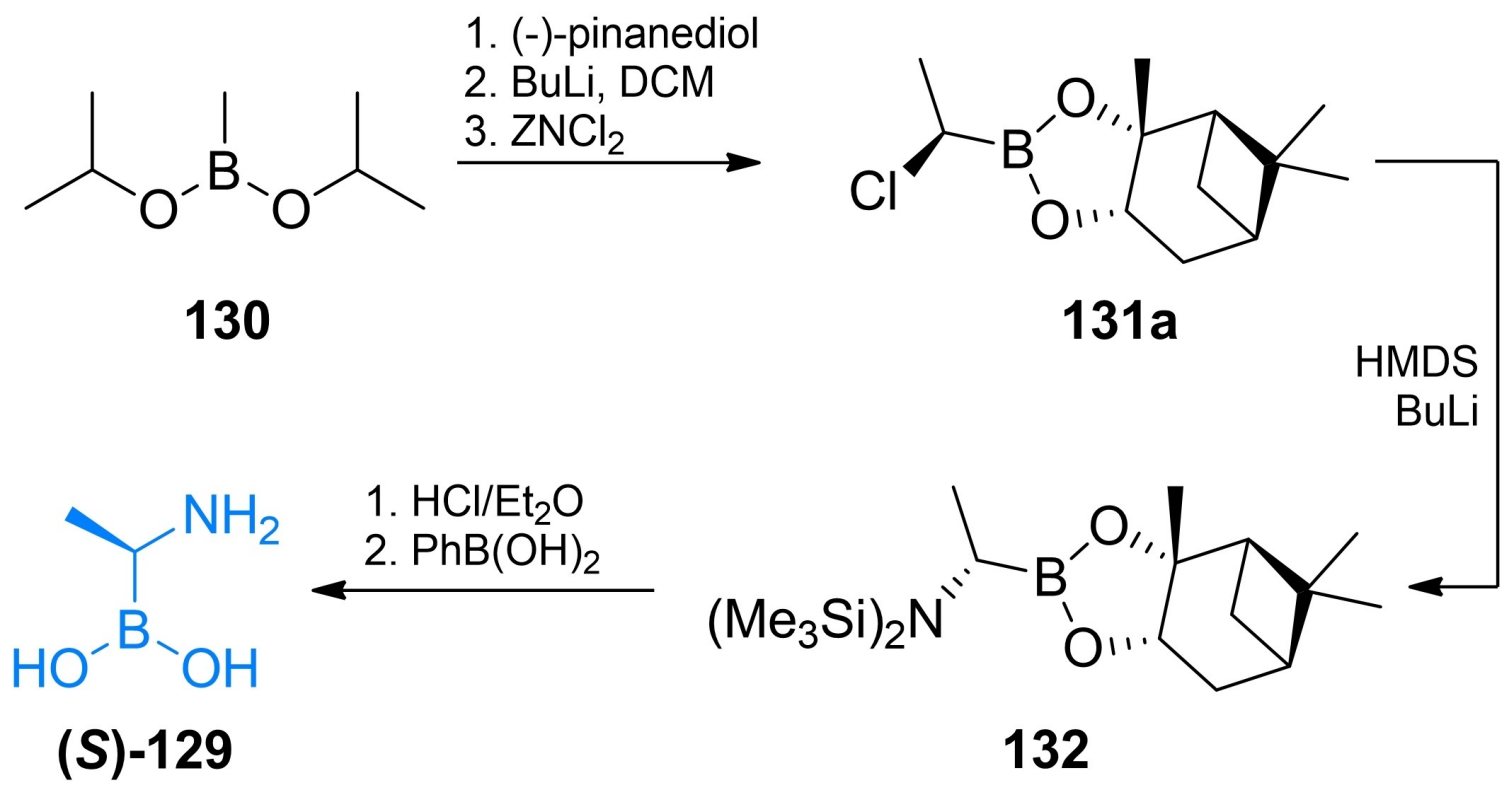
Synthesis of aminoboronic acid‐based antibacterials **(*S*)‐129**.

Both racemic and asymmetric synthesis of d‐Ala(B) ((*S*)‐**129**) was reported by Putty *et al*.^[123]^ In the synthesis of racemic Ala(B) the authors used diisopropylmethylboronate **130** as a starting material. The authors converted into boronate of pinacol and introduced amino function using HMDS. To obtain optically pure d‐Ala authors used of (−)‐pinanediol instead of pinacol. (−)‐Pinanediol derivative was used in *B*‐alkylation reaction which proceeded in stereoselective manner forming (*R*)‐**131 a** derivative which was subsequently reacted with HMDS in the S_N_2 reaction. Hydrolysis of protecting groups with HCl and then with phenylboronic led to the (*S*)‐**129** (Scheme 19).

**Scheme 20:**
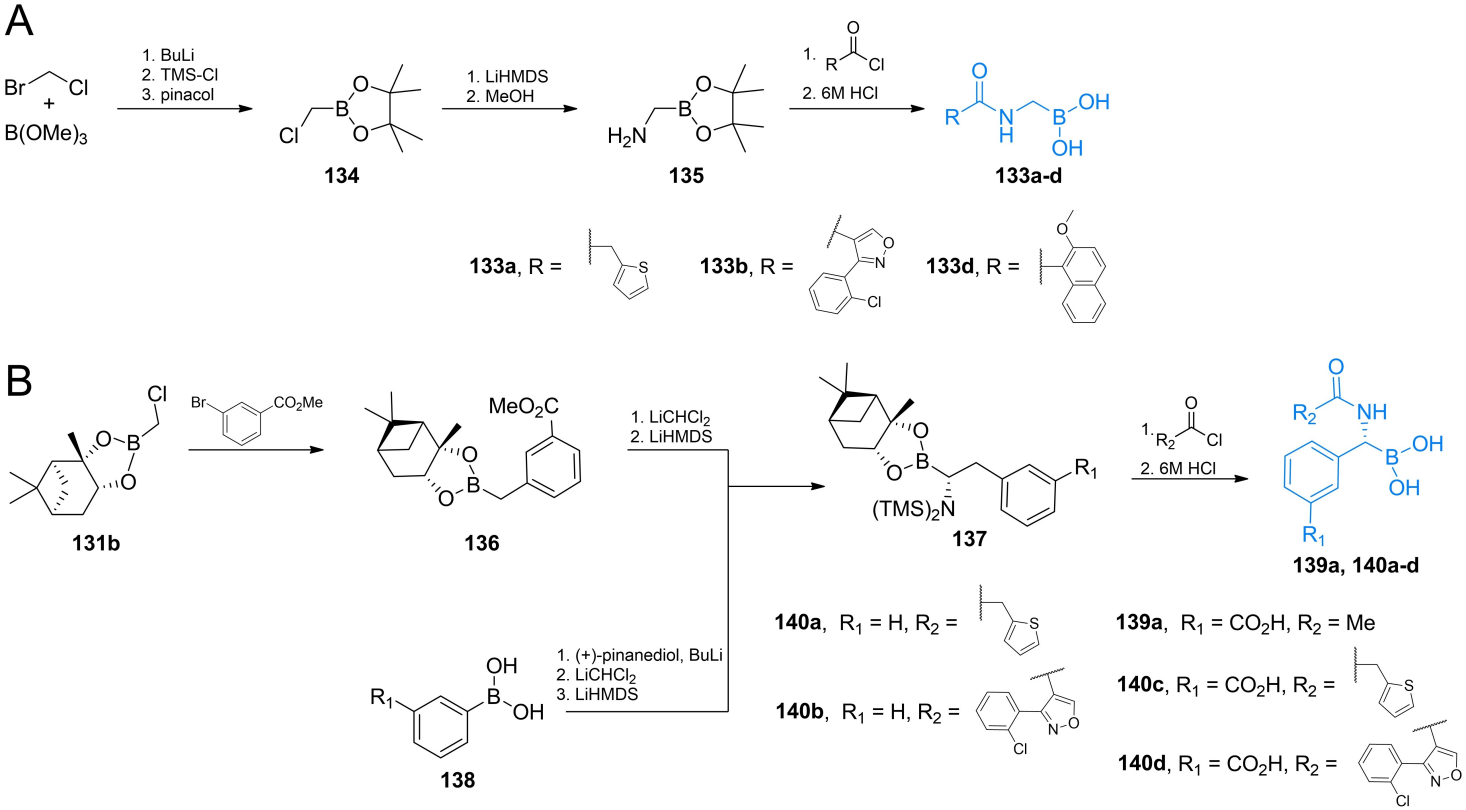
Synthesis of aminoboronic acid‐based antibacterials **133 a**–**d**, **139 a** and **140 a**–**d**.

Acylglycylboronic acids **133 a**–**d** were synthesized using bromochloromethane and trimethyl borite as starting materials (Scheme 20A).[^117^, ^118^, ^124^] The formed chloroalkyl pinacolate of boric acid **134** was then treated with lithium hexamethyldisilazate and methanol to introduce amino‐ function. Amine **135** was then coupled with appropriate acyl chloride. Treatment with aqueous HCl lead to the formation of compounds **133 a**–**d**.

**Scheme 21:**
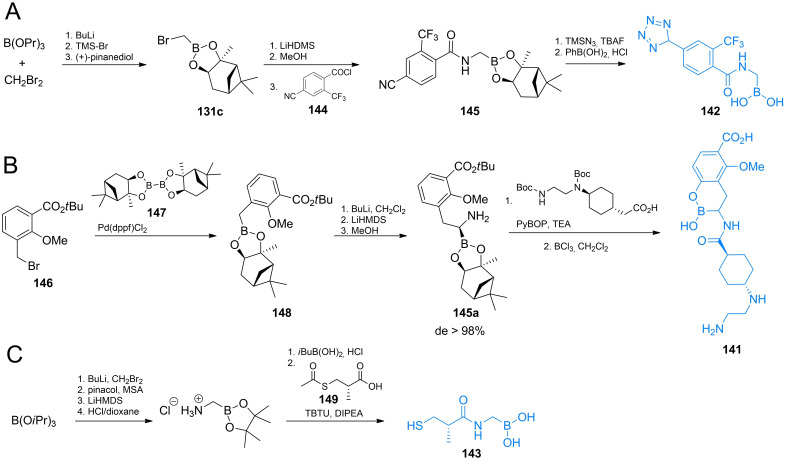
Synthesis of aminoboronic acid‐based antibacterials **141–143**.

Synthesis of optically pure *N*‐acyl‐aryloalanylboronic acids **139 a‐**‐**c** was designed by Martin and co‐workers[^120^, ^121^, ^122^] and applied by Kurz *et al*.^[125]^ The authors used (+)‐pinanedichloromethane boronate which can be obtained in a manner similar to that of the pinacol analog **134** (Scheme 21B). Reagent **131 b** was used in nucleophilic aromatic substitution with appropriate bromobenzene derivative to yield boronate **136** which was subsequently reacted with dichloromethyl lithium and lithium hexamethyldisilazate to form target backbone. Silyl intermediate **137** was treated with respective acyl chloride, reacted with BCl_3_ to cleave methyl‐aryl ethers and treated with 6 M to yield compounds **139 a**–**c**. To obtain *N*‐acyl‐aryloglycylboronic acids Caselli *et al*. used substituted arylboronic acids **138** which were subjected to the same series of reactions like those mentioned above (Scheme 21B). The formed silylated compound **137** can be treated with appropriate acyl chloride to yield compounds **140 a**–**d**.[^126^, ^127^]

Synthesis of compound **142** was designed by Caselli *et al*.[^119^, ^128^] In that strategy, pinanediol boronate **131 c** was treated with HMDS and methanol to introduce amino function and then reacted with aromatic nitrile **144**. The formed compound **145** was reacted with TMS‐azide, what led to the formation of additional tetrazole ring *via* microwave assisted cycloaddition (Scheme 21A).^[54]^ Compound **148** was obtained by treating substituted benzyl bromide **146** with bis[(+)‐pinanediolato]diboron **147** (Scheme 21B). Phenyloalanylboronic acid backbone was obtained following the same strategy as before, using (+)‐pinanediol as a chiral auxiliary in this synthesis led to the formation of product **141** of high optical purity.^[129]^ Compound **143** obtained by Wang *et al*. utilized chloroalkylation of boronates followed by amino group introduction. The formed alkylamino boronate was coupled with carboxylic acid **149** using the TBTU strategy. Subsequent deprotection led to the target compound (Scheme 21).^[130]^

## 3 Antifungal amino acid‐based agents

### 3.1 Molecular targets for amino acid‐based antifungals

Several amino acid compounds of natural or synthetic origin presented in this section have been as antifungals in the past and although none of them have been actually accepted for clinical use, it seems that there is still some space for improvement of their properties, as well as for a search for novel antifungals of this type, especially these aimed at unexploited molecular targets.

As long as amino acid‐based antibacterials described in section 2 target enzymes catalyzing particular steps in peptidoglycan biosynthetic pathway, especially these involved in incorporation of d‐amino acids, there are not any homologs of these enzymes in fungal cells. Nevertheless, several amino acid compounds exhibiting antifungal activity are known but their molecular targets are quite diverse. Undoubtedly, the highest potential in this respect exhibit enzymes participating in fungi‐specific pathways of biosynthesis of protein amino acids, including these of the aspartate family.^[131]^ Surprisingly enough, targets for amino acid‐based antifungals can be also found in biosynthesis of amino sugars, aminoacyl‐tRNA conjugates and ergosterol.

### 3.2 Antifungal glycine derivatives

**Figure 7:**
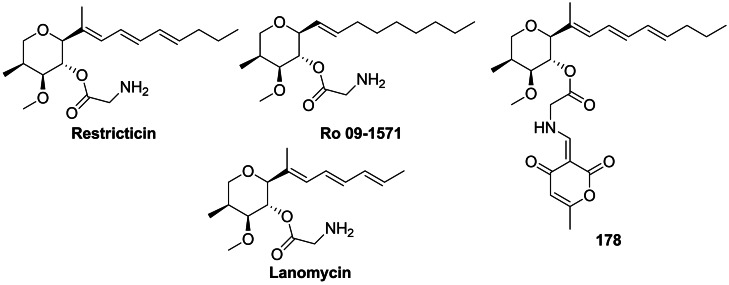
Antifungal agents incorporating glycine or glycine‐based residues: restricticin, lanomycin, Ro 09‐1571 and **178** – an inhibitor of NO production.

Restricticin and Lanomycin are antibiotics produced by *Penicillium restrictum*[^132^, ^133^, ^134^] while Ro 09‐1571 is a synthetic analogue of restricticin, lacking two double bonds. These compounds are glycine esters containing tetrahydropyrene ring with four stereogenic centres (Figure 7). They demonstrate an antifungal activity, with Ro 09‐1571 being the most active in the series. Its activity against *C. albicans* and *A. fumigatus* is similar to that of ketoconazole, while activity against *C. neoformans* is comparable to that of fluconazole.^[135]^ The three compounds are inhibitors of cytochrome P_450_‐dependent lanosterol 14α‐demethylase,^[136]^ an enzyme catalyzing the key reaction in the ergosterol biosynthetic pathway, known also as a molecular target of azole and triazole synthetic antifungals.

**Scheme 22:**
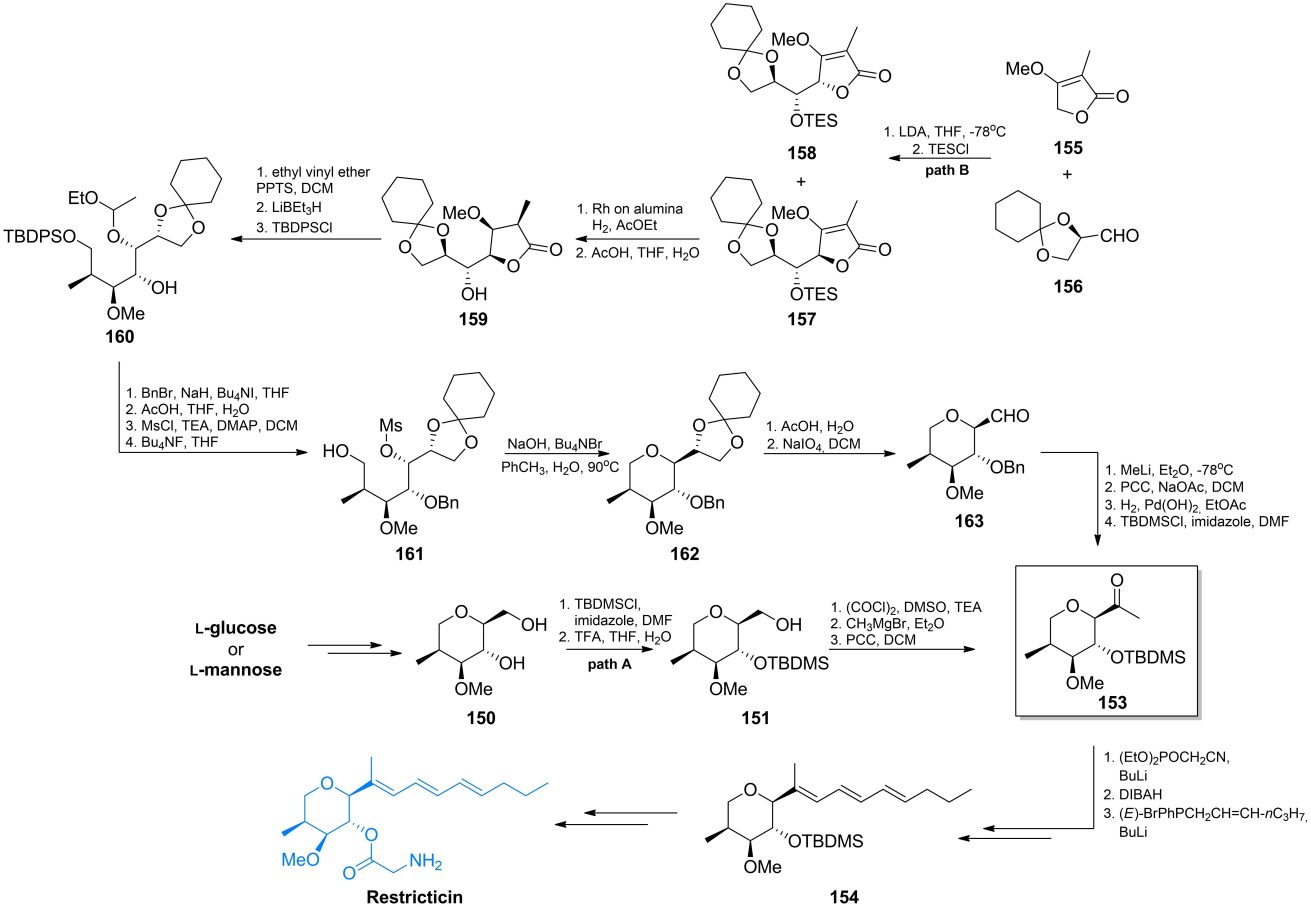
Synthesis of restricticin.

A few methods of a total synthesis of these compounds have been elaborated. In the first syntheses, as a key precursor one of the l‐aldohexoses, namely l‐mannose^[137]^ or l‐glucose^[138]^ was employed, to ensure a proper configuration of stereogenic centres at C2, C3 and C4 atoms. In the initial steps of the first synthesis of restricticin starting from l‐mannose, conversion of the hydroxymethin C1 atom into methylene and the C2 hydroxyl into methyl afforded the tetrahydropyrane ring of stereochemistry corresponding to that of restricticin (**150**, Scheme 22, path A). In the next step, the secondary hydroxyl of **150** was selectively silylated to give **151**. This intermediate was converted into methylketone **153**, which is a substrate for construction of a triene side chain. This was done using the modified Wittig reaction under Horner‐Emmons conditions. A mixture of three stereoisomers was thus obtained and they were separated by liquid chromatography. The isolated *all*‐E isomer **154**, after removal of the silyl protection from C4 hydroxyl was treated with TEOC‐glycine. Alkylation of C3 hydroxyl afforded restricticin as the final product.^[137]^

Honda *et al*. proposed an alternative way of stereoselective synthesis of the key precursor **153** (Scheme 22, path B) employing reaction of the lithium salt of α‐methyltetronate **155** with (*R*)‐2,3‐*O*‐cyclohexylideneglyceraldehyde **156**.^[139]^ This reaction afforded mixture of the expected adduct **157** and a side product **158**, with 66 % and 16 % yields, respectively. The catalytic reduction of **157** with H_2_ over rhodium on alumina gave stereoselectively the γ‐lactone **159**, as the sole product in quantitative yield. Subsequently, the silyl group of **159** was exchanged for ethoxyethyl with simultaneous formation of a new stereogenic centre (**160**). Reduction of the γ‐lactone with LiBHEt_3_ and subsequent selective silylation of the primary hydroxyl led to the ether **160**. This compound possesses four neighboring stereogenic centers, out of which three have the same configuration as those at C3, C4 and C5 of restricticinol. In the following steps, the protective groups were either introduced or removed to give alcohol **161**. This intermediate was subjected to intramolecular S_N_2 reaction with aqueous NaOH in the presence of a phase transfer catalyst, leading to the tetrahydropyrane derivative **162** with the proper configuration at C2. Hydrolysis of the cyclohexylidenyl group and then cleavage of the resulting diol with sodium periodate, afforded aldehyde **163**. Introduction of the methyl group to **163** upon the action of methyllithium gave diasteroisomeric alcohols, which when oxidized with pyridinium chlorochromate (PCC) afforded ketone. Exchange of the benzyl protection for silyl resulted in the ultimate formation of **153** (Scheme 22).

**Scheme 23:**
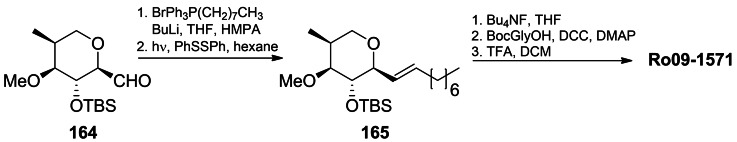
Synthesis of Ro 09‐1571.

Starting from the aldehyde **164** obtained from l‐glucose, the Ro 09‐1571 compound was obtained.^[138]^ **164** was converted in a Wittig‐type reaction into olefin of *Z* configuration, that was isomerized to *E*‐**165** by the photochemical reaction. After removal of the silyl protection, the alcohol was esterified with Boc‐glycine. Removal of the Boc protection under acidolytic conditions led to Ro 09‐1571 in the TFA salt form (Scheme 23).

**Scheme 24:**
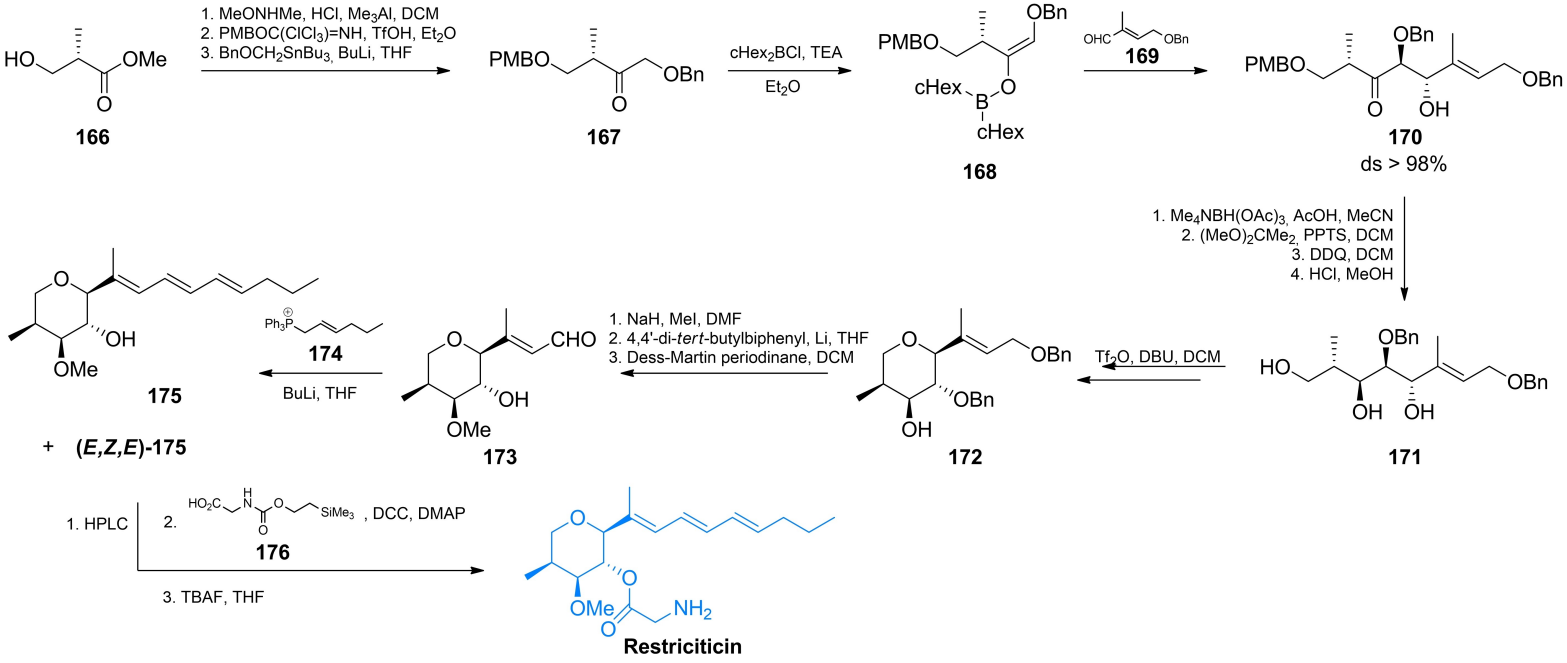
Total synthesis of restricticin *via* the boron‐mediated anti aldol reaction.

Another approach was presented by Paterson *et al*., who carried out an elegant total synthesis of (+)‐restricticin in 12 steps, starting from the ketone **167** of *S* configuration.^[140]^ In that scheme, there were two key steps :  *(a)* the boron‐mediated *anti* aldol reaction of (*S*)‐**167** with **169** to give **170** and *(b)* the cyclisation of **171** to **172** (Scheme 24). The protected ketone **167** was prepared from the commercially available methyl (*S*)‐2‐methyl‐3‐hydroxypropionate (**166**). In the Weinreb amide formed, the hydroxyl group was protected with PMB and this product was treated with benzyloxymethyl lithium what afforded **167**. The *anti* aldol reaction between *E*‐enol borinate **168** and (*E*)‐2‐methyl‐3‐benzyloxybutenal **169** was highly diastereoselective (>98 % ds), resulting in formation of pure **170** isomer. Reduction of **170** with Me_4_NBH(OAc)_3_ afforded 1,3‐anti‐diol with 86 % ds and after removal of protection groups, gave triol **223**. The key cyclisation of **171** to **171** was performed by the selective formation of a primary triflate. The thus obtained tetrahydropyrane **172** was converted into methyl ether and selectively oxidized using the Dess‐Martin periodinane. The resulting aldehyde **173** was subjected to the Wittig reaction with an ylide generated from **174**, to give a mixture of (*E,E,E*)‐restricticinol (**175**) and its (*E,Z,E*)‐isomer in 90 % yield, which were separated by HPLC. Finally, restricticinol **175** was treated with the glycine derivative **176**, what after removal of the protective groups afforded restrictricin (Scheme 24).

Another precursor of the tetrahydropyrane ring was d‐tartaric acid. (+)‐Lanomycin was synthesized from the aldehyde derived from d‐tartaric acid.^[141]^

Interestingly, the restricticin derivative recently isolated from the marine fungus *Penicillium janthinellum* 168CLC‐17.1, in which the amino group of the glycine residue is substituted with an α‐pyrone ring, was devoid of antifungal activity.^[142]^ On the other hand, a derivative of restricticin **178** (Figure 7) exhibited inhibitory activity on NO production in LPS‐stimulated BV‐2 microglial cells. Moreover, the restricticins suppressed iNOS and COX‐2 expression (both at the protein and mRNA levels), and also inhibited the LPS‐induced production of pro‐inflammatory cytokines.

### 3.3 Compounds targeting enzymes involved in biosynthesis of amino acids of the aspartate family

**Figure 8:**
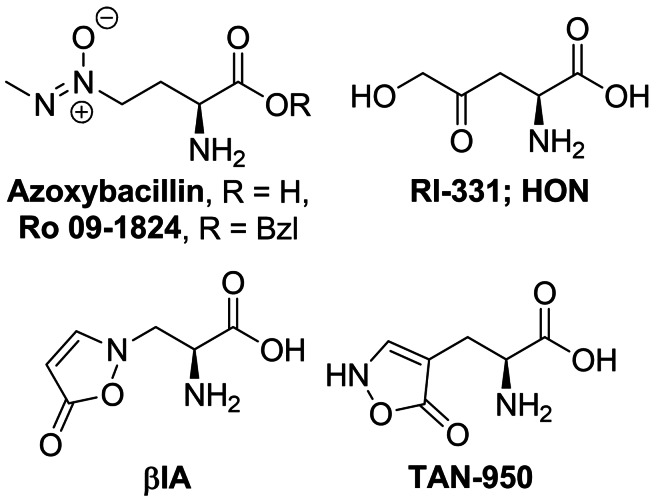
Amino acid biosynthesis inhibitors: azoxybacillin, (*S*)‐2‐amino‐4‐oxo‐5‐hydroxypentanoic acid (HON), and alanine derivatives – β‐(isoxazolin‐5‐one‐2‐yl)alanine (βIA) and β‐(isoxazolin‐5‐one‐4‐yl)alanine (TAN‐950).

Azoxybacillin (Figure 8) is an antifungal antibiotic produced by *Bacillus cereus*.^[143]^ This is one of the very few compounds of natural origin containing the azoxy group.^[144]^ Azoxybacilin exhibits a broad spectrum of antifungal activity in methionine‐free environments. The mechanism of antifungal action of this antibiotic is connected with a fungi‐specific pathway of l‐methionine biosynthesis,^[145]^ particularly at the sulfur acquisition step, by influence on expression of two genes involved in regulation of sulfite reductase activity, namely *MET4* and *MET10.17*.^[146]^ The antifungal action of azoxybacillin is antagonized by l‐methionine^[147]^ but its benzyl ester, Ro 09‐1824, is active also in methionine‐containing media.^[148]^

**Scheme 25:**
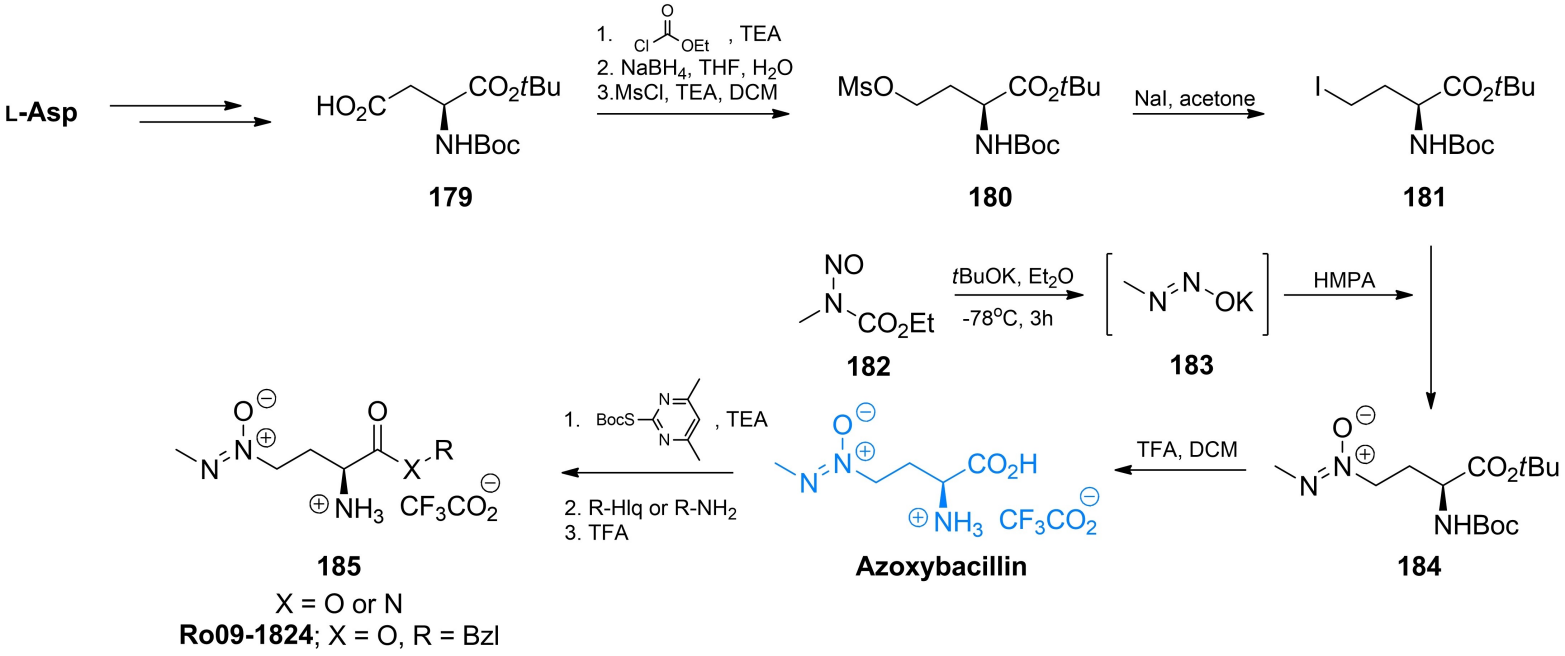
Synthesis of azoxybacillin and their derivatives.

**Scheme 26:**
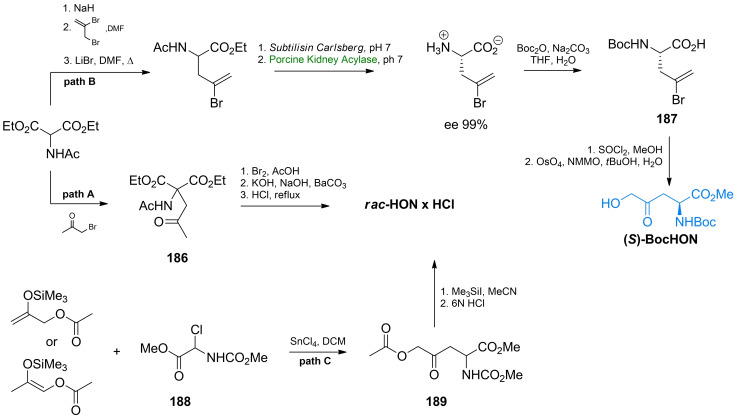
Synthesis of δ‐hydroxy‐γ‐oxonorvaline starting from diethyl *N*‐acetamidomalonate or chloroglycine derivative.

The key step in azoxybacillin and Ro 09‐1824 synthesis^[147]^ is introduction of the azoxy moiety upon the region‐ and stereoselective alkylation of diazoate anion **183** proposed by Moss *et al*.^[149]^ The diazoate anion is generated from *N*‐alkyl‐*N*‐nitrosourethane **182** upon the action of potassium *tert*‐butoxide. The iododerivative **181** was obtained from Boc‐l‐AspO*t*Bu **179** by the four‐step procedure of Olsen *et al*.^[149]^ Reaction between **181** and **183** afforded protected azoxybacillin **184** (Scheme 25). Deprotection and subsequent introduction of Boc at azoxybacillin amino group gave rise to preparation of azoxybacillin esters and amides, including Ro 09‐1824.^[147]^

(*S*)‐2‐Amino‐4‐oxo‐5‐hydroxypentanoic acid, known as RI‐331 or HON (Figure 8) is a product of secondary metabolism of *Streptomyces akiyoshiensis* nov. sp. It is an antifungal agents with a broad spectrum of activity against human pathogenic fungi,^[150]^ although was originally discovered in an antimycobacterial screen about 60 years ago.^[151]^ Its mechanism of action includes inhibition of homoserine dehydrogenase, responsible for the conversion of aspartate semialdehyde into homoserine in the biosynthetic pathway of biosynthesis of amino acids of aspartate family.^[152]^ RI‐331 is a mechanism‐based inhibitor of this enzyme and its interaction with the target involves enzyme‐dependent formation of a covalent adduct between C4 of the nicotinamide ring of NAD^+^ and C5 of HON.^[153]^ The structure of this non‐proteinogenic amino acid was determined by Miyake, who also elaborated a method of its synthesis. The first step in this procedure was condensation of diethyl *N*‐acetamidomalonate and bromoacetone. The condensation product **186** was brominated and the bromine atom was subsequently exchanged for hydroxyl. Hydrolysis of this product gave racemic δ‐hydroxy‐γ‐oxo‐norvaline (Scheme 26, path A). Conversion into brucine salt of *N*‐acetyl‐δ‐hydroxy‐γ‐oxo‐dl‐norvaline allowed separation of diastereoisomers and subsequent isolation of optically pure HON enantiomers.^[154]^

Leanna *et al*. proposed *tert*‐butoxycarbonyl‐2‐bromoallyl‐l‐glycine **187**, as an universal synthon in synthesis of non‐protein amino acids.^[155]^ This compound is obtained in condensation of acetamidomalonate and 2,3‐dibromopropene (Scheme 26, path B). Another starting substrate in such syntheses is a protected amino acid **189** obtained from chloroglycine **188** in reaction with silyl enol ethers, in the presence of tin tetrachloride (Scheme 26, path C). This synthon can be easily converted into racemic δ‐hydroxy‐γ‐oxonorvaline.^[156]^

**Scheme 27:**
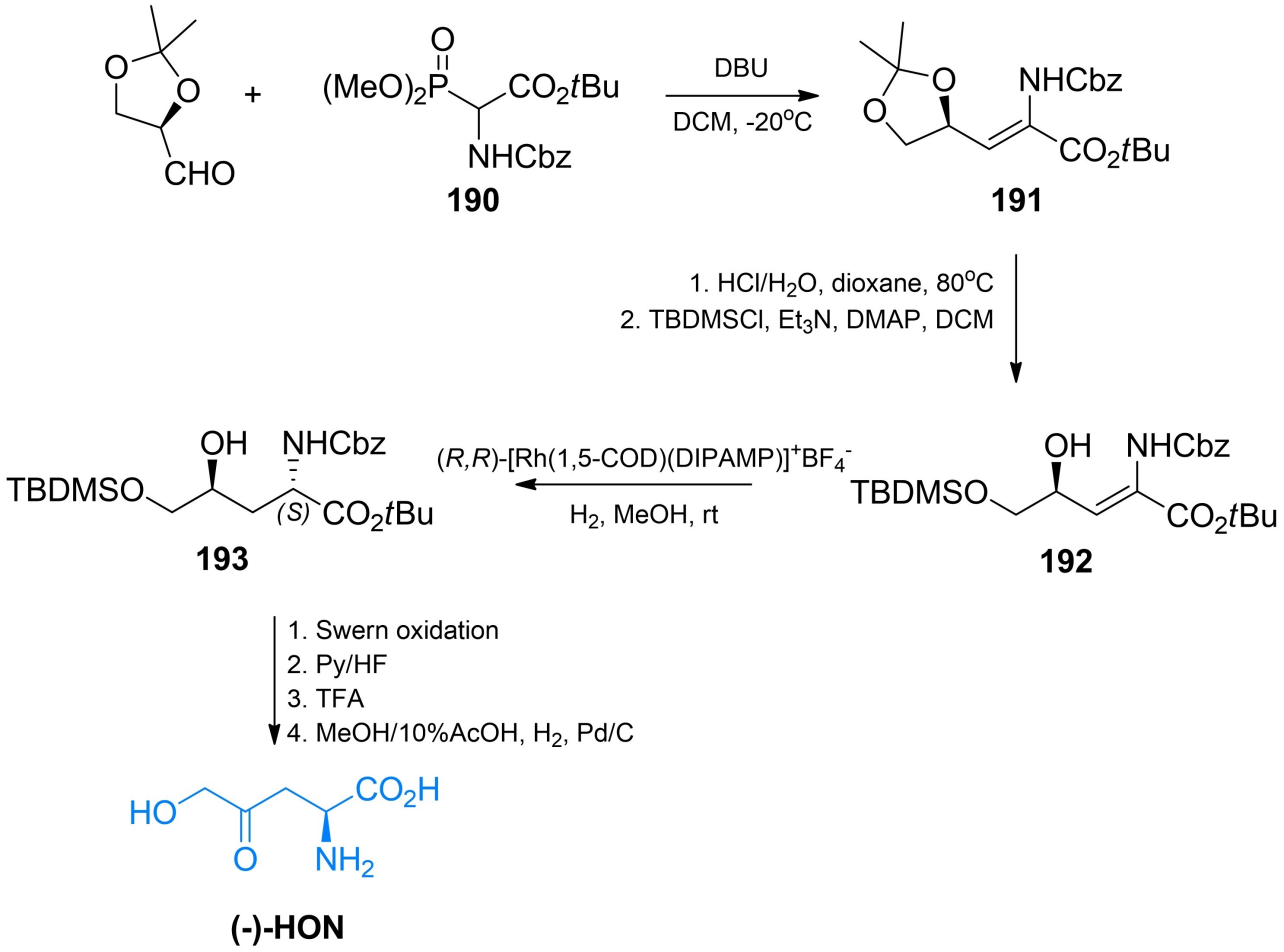
The stereoselective synthesis of (*S*)‐δ‐hydroxy‐γ‐norvaline.

**Scheme 28:**
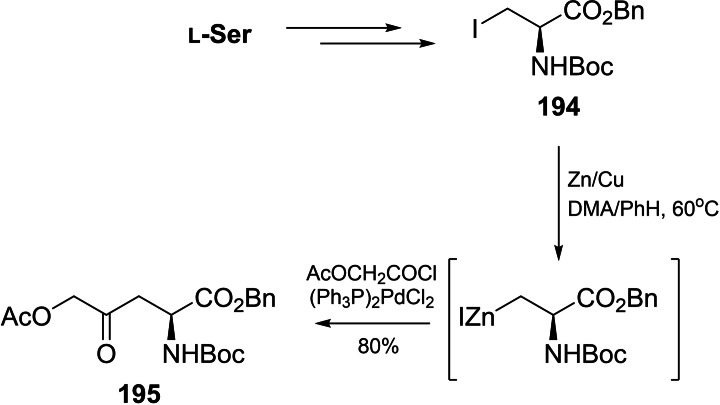
The stereoselective synthesis of protected δ‐hydroxy‐γ‐(*S*)‐norvaline from l‐serine.

**Scheme 29:**
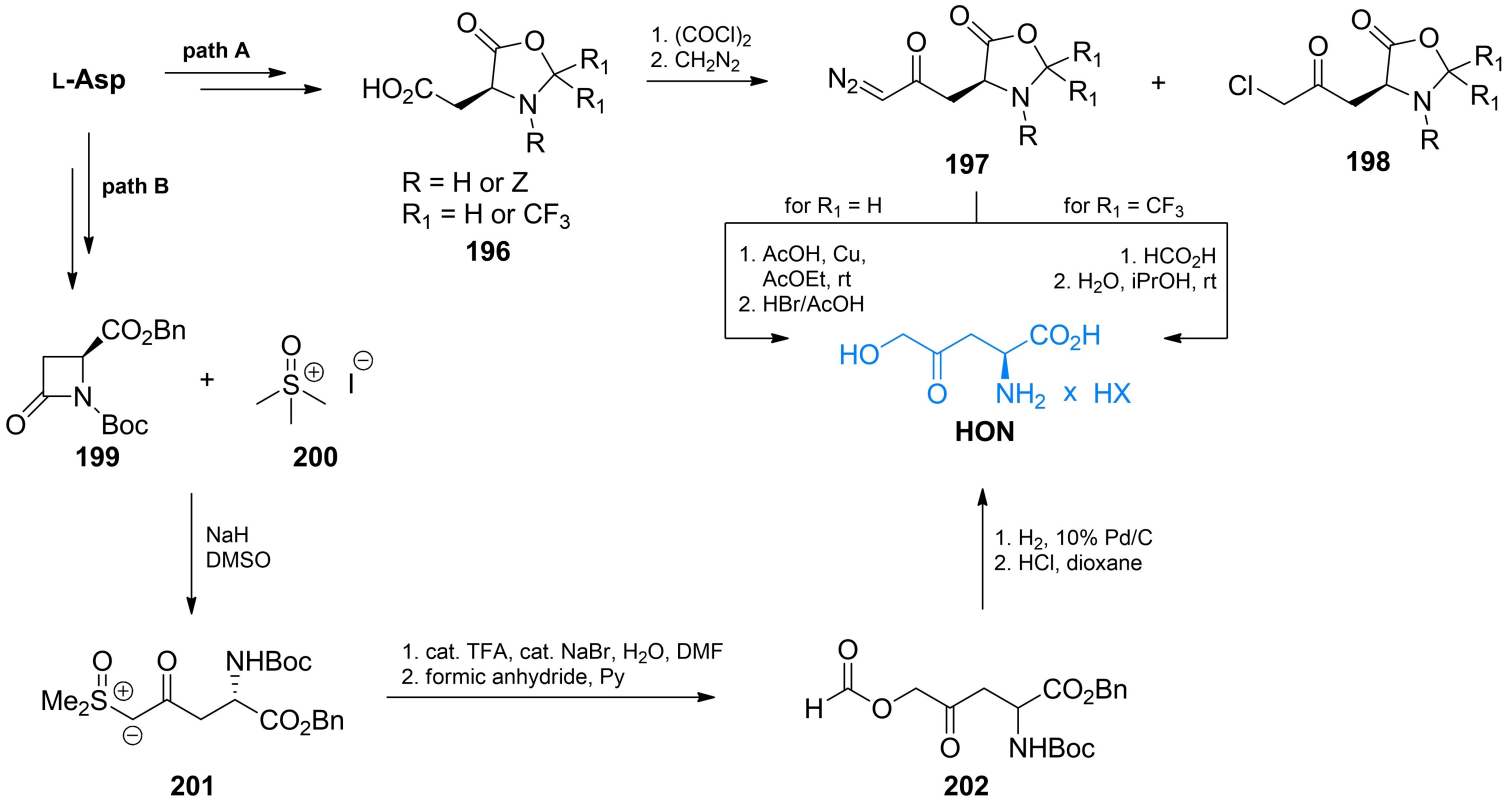
The stereoselective synthesis of δ‐hydroxy‐γ‐(*S*)‐norvaline from l‐aspartic acid.

In other methods of HON synthesis, an optically pure glyceraldehyde^[157]^ or α‐amino acids were used as starting substrates, to ensure a proper configuration at the C2 atom. In the first proposed stereoselective synthesis of (‐)‐HON, (*R*)‐2,3‐*O*‐isopropylideneglyceraldehyde was condensed with *tert*‐butyl 2‐benzyloxycarbonylamino‐2‐(dimethoxyphosphoryl)acetate **190** (Scheme 27). Then, a dioxolane ring of the condensation product **191** was cleaved, with the formation of the silyl ether **192**. The key step in that scheme was a subsequent diastereoselective hydrogenation using (*R,R*)‐[Rh(1,5‐COD)(DIPAMP)]^+^BF_4_^−^ what led to the formation of the (2*S*,3*S*)‐**193** intermediate. Oxidation of **193** with oxalyl chloride/dimethyl sulfoxide afforded a fully protected HON. After deprotection, (*S*)‐δ‐hydroxy‐γ‐oxo‐norvaline identical with RI‐331 of natural origin was obtained.^[157]^

l‐Serine or aspartic acid were also used as the amino acid substrates in HON synthesis. l‐Serine was converted into β‐iodoalanine **194**, which was subsequently acylated with acetoxyacetyl chloride in the presence of bis(triphenylphosphine)palladium dichloride as a catalyst, to give enantiomerically pure protected HON derivative **195** (Scheme 28).[^158^, ^159^]

l‐Aspartate is a convenient substrate for preparation of δ‐hydroxy‐γ‐oxo‐norvaline. This amino acid, upon reaction with formaldehyde^[160]^ or hexafluoroactetone,[^161^, ^162^] forms an oxazolidin‐5‐one **196** affording a simultaneous protection of amino and α‐carboxyl functionalities. The free ω‐carboxyl can be then converted into acyl chloride and treated with diazomethane. Decomposition of diazoketone **197** with carboxylic acids gives HON as the final product. (Scheme 29, path A). To minimize a side reaction (HCl release and chloroketone **198** formation), the acyl chloride is gradually added to the three‐fold molar excess of diazomethane. The **197** and **198** products can be easily separated by column chromatography or distillation. In an alternative method, l‐aspartic acid was converted into β‐lactam **199**, which was subjected to the reaction with trimethylsulfoxonium iodide **200**. In consequence of ring opening, the α‐keto sulfoxonium species **201** was formed. Protonation of ylide **201** and subsequent reaction with formic anhydride led to the removal of the sulfoxonium group and formation of the HON derivative **202**, which after deprotection was converted into HON (Scheme 29, path B).^[163]^

**Scheme 30:**
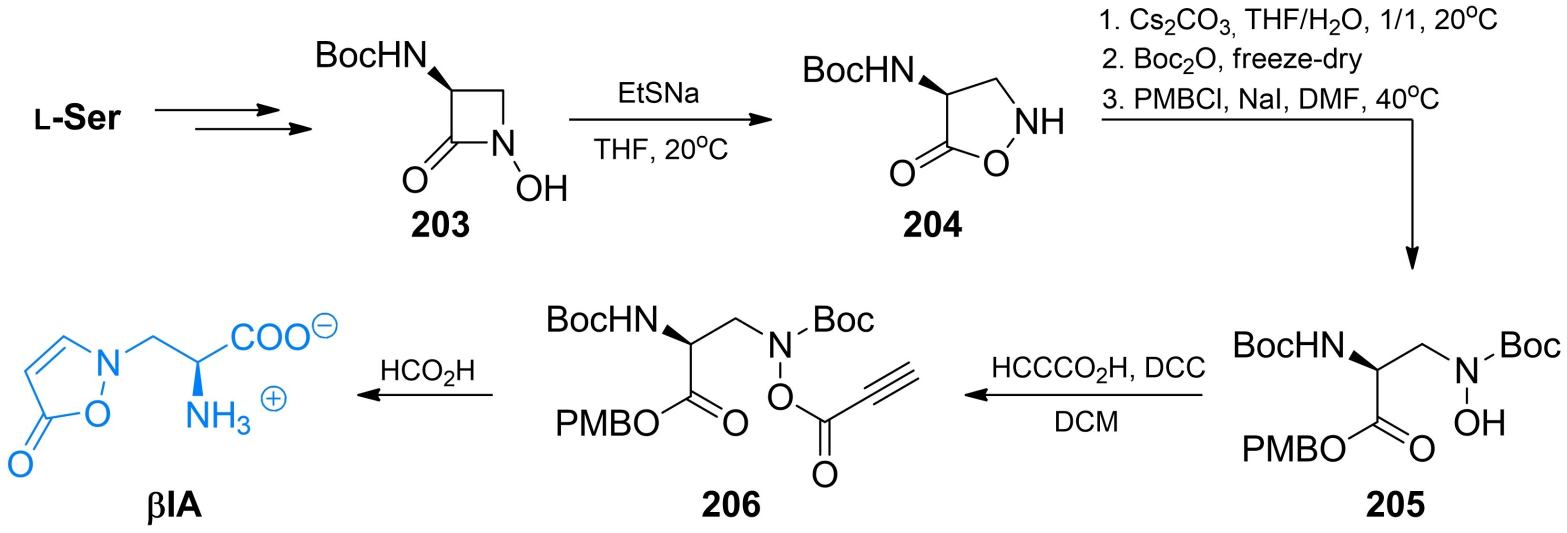
Synthesis of l‐β‐(isoxazolin‐5‐one‐2‐yl)alanine.

A non‐protein alanine derivative, namely l‐β‐(isoxazolin‐5‐one‐2‐yl)‐alanine, known as βIA (Figure 8), was isolated from pea (*P. sativum*) seedlings^[164]^ and much later found to demonstrate a broad spectrum of antifungal activity.^[165]^ The antifungal action of this compound is reversed by l‐methionine, l‐cysteine and l‐homocysteine,^[165]^ what may suggest one of the enzymes of the l‐methionine biosynthetic pathway as a possible molecular target. Preparation of βIA is possible from *O*‐acetyl‐l‐serine and isoxazolin‐5‐one in a single reaction catalyzed by the *P. sativum* seedling extract.^[166]^ A purely chemical synthesis was developed by Baldwin *et al*.^[167]^ In that approach, a protected *N*‐hydroxy‐β‐lactam **203** prepared from l‐serine was a starting substrate. It was isomerized to isoxazolidin‐5‐on **204**, in which after ring opening, the carboxyl functionality and nitrogen atom of l‐hydroxylamine were protected. Selective *O*‐acylation of thus formed **205** with propynoic acid in the presence of DCC led to *O*‐propynoylhydroxylamine **206**, which after removal of the protecting groups underwent a spontaneous cyclization in the presence of formic acid to give βIA (Scheme 30).

Some antifungal activity is also demonstrated by the βIA constitutive isomer TAN‐950, produced by *Streptomyces sp*. A136 (Figure 8).^[168]^

### 3.4 Proline analogs inhibiting Ile‐tRNA^Ile^ synthetase

**Figure 9:**
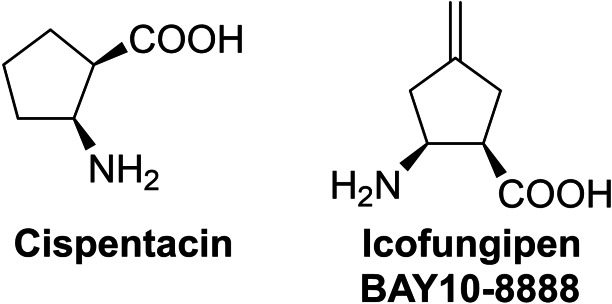
Antifungal proline analogs: cispentacin and icofungipen.

Probably the most popular antifungal amino acid is *cis*‐2‐aminocyclopentane‐1‐carboxylic acid, known as cispentacin or Fr109615 (Figure 9). The (1*R*,2*S*) enantiomer of this compound was isolated in 1989 from fermentation broth of *Bacillus cereus* ^[169]^ and later from S*treptomyces setonii* ^[170]^ and shown to exhibit strong antifungal activity, especially against *Candida albicans*. Interestingly, the (1*S*,2*R*) enantiomer of *cis*‐2‐aminocyclopentane‐1‐carboxylic acid is completely devoid of antifungal activity. Through active transport by the proline‐specific permease, this compound accumulates in fungal cells up to 200‐fold of the extracellular concentration. Its intracellular molecular target is isoleucyl‐tRNA^Ile^ synthetase (IleRS).^[171]^ Icofungipen, previously known as BAY 10‐8888 (Figure 9), discovered through a program directed toward a more potent cispentacin derivative, follows the same mechanism of action.^[172]^

Most of the methods of cispentacin synthesis were based on the rationale of preparation of a racemic form of this cyclic β‐amino acid, followed by the separation of enantiomeric antipodes through the crystallization of diastereomeric salts of the protected racemic cispentacin with (+)‐dehydroabietylamine,^[169]^ (+)‐(*R*)‐α‐methylbenzylamine^[173]^ or (−)‐ephedrine.^[174]^ In another approach, racemic cispentacin was conjugated with Boc‐l‐phenylalanine to form diasteroisomeric dipeptides, subjected to fractional crystallization, followed by Edman degradation.[^175^, ^176^] Enantiomerically pure cispentacin could be also obtained by selective enzymatic hydrolysis of *N*‐acyl‐,^[177]^ β‐lactam[^178^, ^179^, ^180^, ^181^, ^182^] or ester^[183]^ derivatives of racemic cispentacin by lipase, lipolase or EN2 A‐1. The racemic cispentacin could be easily obtained by 1,2‐dipolar cycloaddition of chlorosulfonyl isocyanate (CSI) to cyclopentene[^169^, ^175^, ^176^, ^180^] or 1,3‐cyclopentadiene[^178^, ^184^] or by Michael addition of ammonia to ester of 1‐cyclopentenecarboxylic acid.^[174]^

**Scheme 31:**
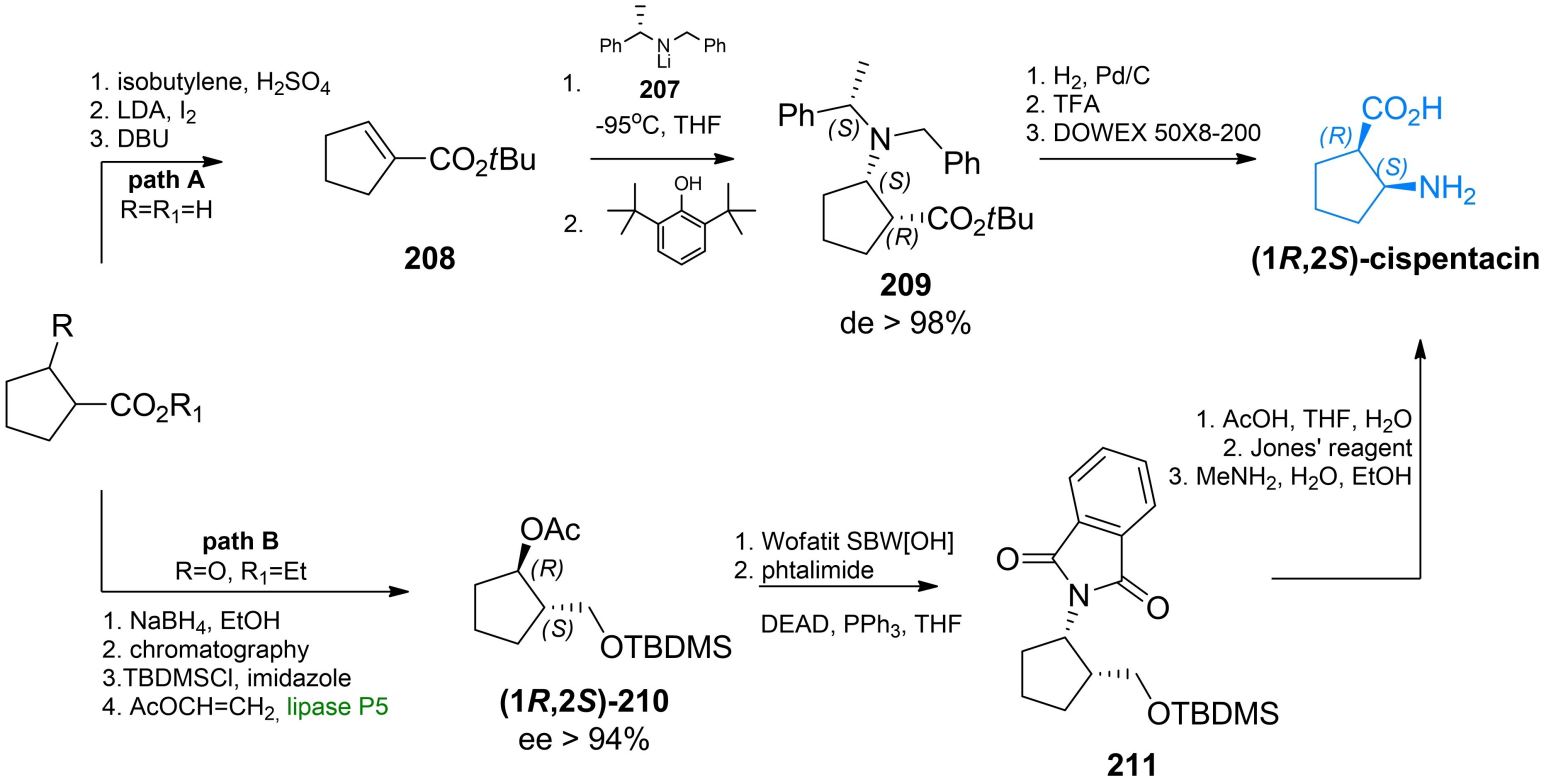
The stereoselective synthesis of (1*R*,2*S*)‐cispentacin with the use of A) chiral auxiliaries; B) lipase.

The first asymmetric synthesis of cispentacin was performed by Davies *et al*., through the diastereoselective Michael addition of lithium (*S*)‐*N*‐benzyl‐*N*‐α‐methylbenzylamide **207** to *tert*‐butyl 1‐cyclopentene‐1‐carboxylate **208**.^[185]^ This reaction was characterized by the high diastereofacial control, leading to the (1*R*,2*S*)‐**209** derivative with >98 % diasteroisomeric excess. Compound **209** was debenzylated by hydrogenolysis, and acidic hydrolysis finally afforded (1*R*,2*S*)‐cispentacin (Scheme 31, path A). Theil *et al*. proposed a chemoenzymatic synthesis of cispentacin, based on the lipase‐catalyzed kinetic resolution of the silyloxyalcohol by transesterification with vinyl acetate in the presence of lipase.^[186]^ The key step in this synthesis was substitutions of the secondary hydroxyl group with phthalimide under Mitsunobu conditions, with inversion of configuration at C1. Subsequent cleavage of the silyl ether **211**, alcohol oxidation with the Jones’ reagent and removal of the protective group led to (1*R*,2*S*)‐cispentacin in enantiomerically pure form (Scheme 31, path B).

**Scheme 32:**
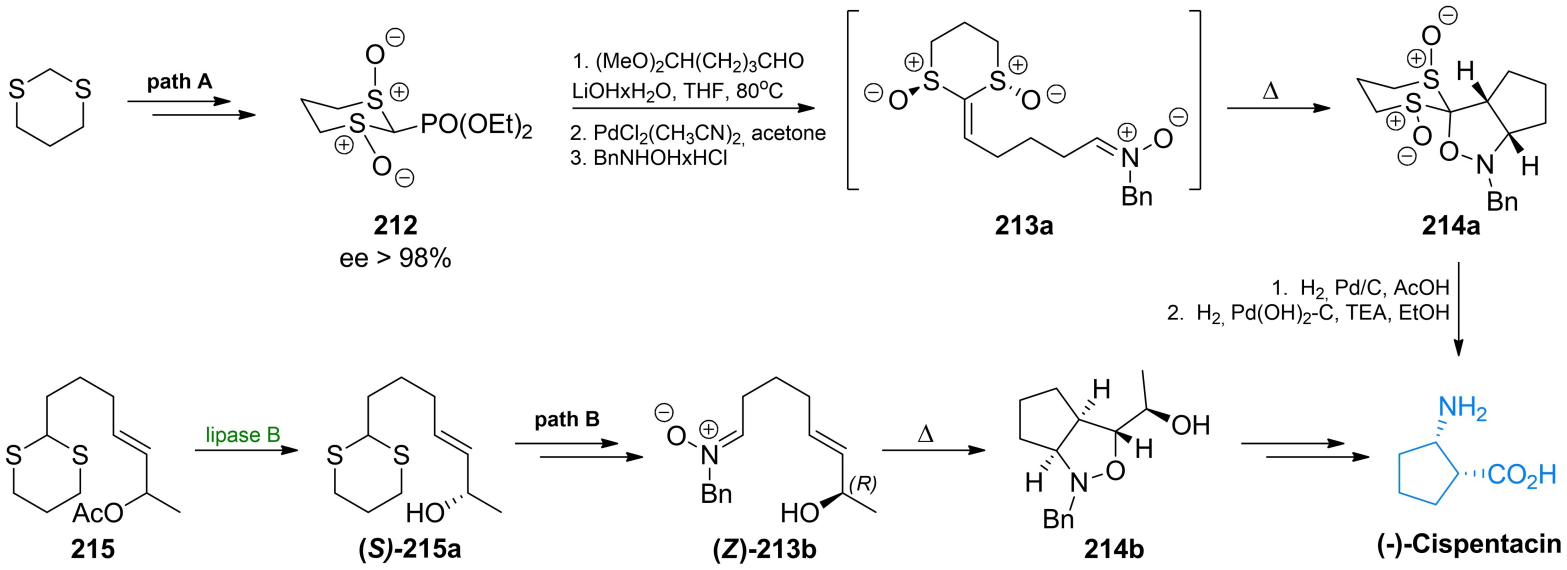
The diastereoselective synthesis of (1*R*,2*S*)‐cispentacin with the use of intramolecular nitrone cycloaddition.

Aggarwal *et al*. presented synthesis of cispentacin based on a highly diastereoselective intramolecular 1,3‐dipolar cycloaddition of a nitrone to a chiral keten equivalent **213 a**,[^187^, ^188^] which was obtained due to the Horner‐Wadsworth‐Emmons olefination of one of the enantiomers of bis‐sulfinyl phosphonate **212**. The cycloaddition leads to a single diasteroisomer of 5,5‐disubstituted *cis*‐isoxazolidine **214 a** (Scheme 32, path A). Previously, an analogous reaction was applied by Konosu *et al*.,^[189]^ who prepared an appropriate nitrone **213 b** from the chiral aldehyde (Scheme 32, path B). A catalytic hydrogenolysis of **214** led to cispentacin as the final product.

**Scheme 33:**
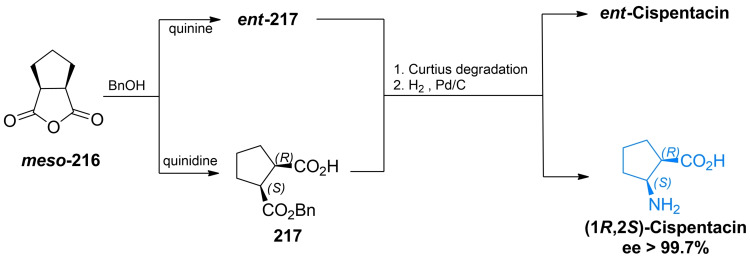
The simple and highly enantioselective synthesis of cispentacin and its enantiomer by the cinchona alkaloid‐mediated opening of prochiral cyclic anhydrides.

Bolm *et al*.[^190^, ^191^] developed a three‐step sequence relying on the quinidine‐promoted asymmetric desymmetrization of the *meso*‐anhydride of *cis*‐1,2‐cyclopentanedicarboxylic acid **216**, followed by Curtius degradation of the acyl azide, derived from the optically active hemiester **217** (Scheme 33). This method was also used for preparation of BAY 10‐8888 (Figure 9), a synthetic cispentacin analogue.[^192^, ^193^]

Another highly enantioselective way of cispentacin preparation is a tandem Michael addition/cycloaddition of cyclopentene‐2‐carbaldehyde to *N*‐Cbz‐hydroxylamine, run in the presence of a chiral catalyst (*R*)‐diphenylprolinol trimethylsilyl ether and benzoic acid as a co‐catalyst.^[194]^

**Scheme 34:**
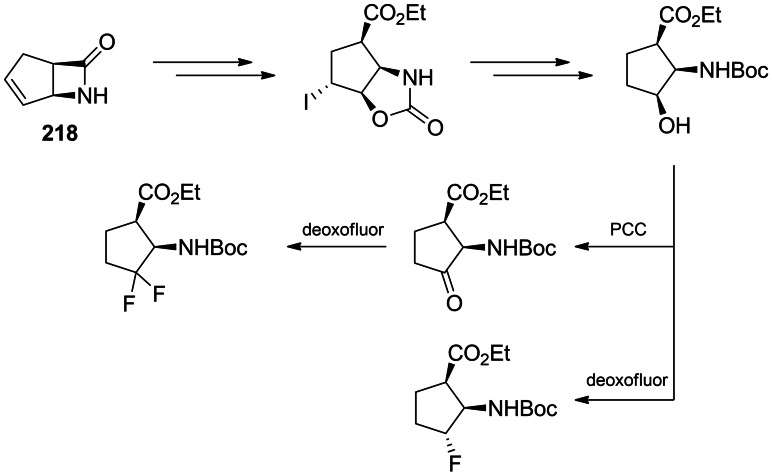
Synthesis of fluoro derivatives of cispentacin.

Mono‐ and difluoro analogues of cispentacin were obtained from respective hydroxy‐ or keto‐ derivatives, as shown in Scheme 34. The starting substrate was enatiomerically pure bicyclic β‐lactam **218** prepared due to the lipase‐driven hydrolysis of the racemic compound. **218** was regio‐ and stereoselectively hydroxylated through the iodooxazoline formation and then the hydroxyl group was exchanged for fluorine by Deoxofluor.[^195^, ^196^]

### 3.5 Inhibitors of the first committed step in UDP‐GlcNAc biosynthesis

**Figure 10:**
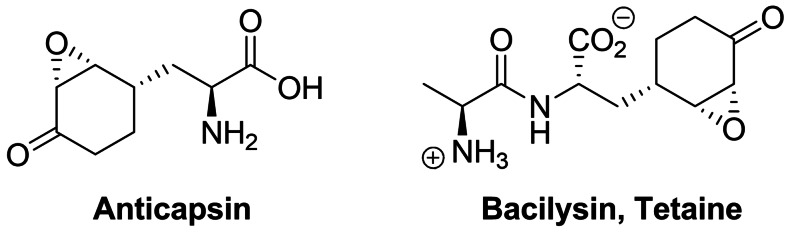
Inhibitors of chitin biosynthesis: anticapsin and l‐alanyl‐anticapsine (tetaine).

Anticapsin ((2*S*)‐2‐amino‐3‐[(1*R*,2*R*,6*R*)‐5‐oxo‐7‐oksabicyclo[4.1.0]heptan‐2‐yl]propanoic acid, Figure 10) is an amino acid antibiotic of antibacterial and antifungal action, produced by *Streptomyces griseoplanus* ^[197]^ and *Bacillus subtilis*.^[198]^ This compound is an irreversible inhibitor of glucosamine‐6‐phosphate synthase, the key enzyme in the biosynthetic pathway of UDP‐GlcNAc formation, which is a sugar‐nucleotide precursor of bacterial peptidoglycan and fungal chitin. Anticapsin binds at the enzyme active site as a structural analogue of one of its substrates, l‐glutamine and forms a covalent bond due to alkylation of the catalytic Cys1 residue by epoxide functionality of the antibiotic molecule.^[199]^ The antimicrobial activity of anticapsin is however poor, due to its inefficient uptake by bacterial and fungal cells. On the other hand, this activity is much better for a dipeptide antibiotic tetaine (bacilysin, Figure 10),^[200]^ in which anticapsin is the C‐terminal amino acid.^[198]^

**Scheme 35:**
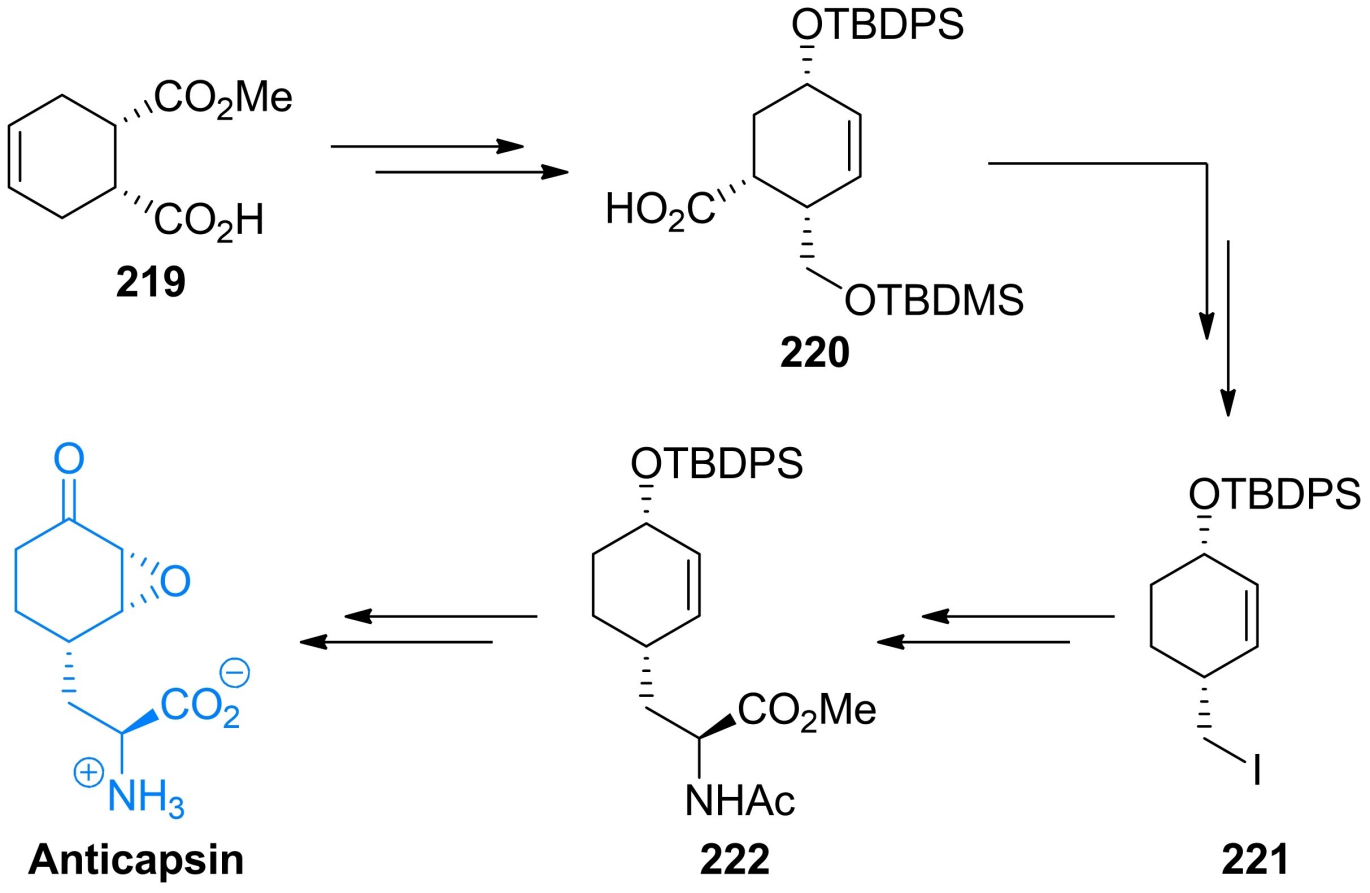
The enatioselective synthesis of anticapsin.

In anticapsin structure, originally determined based on the CD, IR and ^1^H NMR spectroscopy data, a *E* configuration was mistakenly assigned to the epoxide ring,^[201]^ corrected for a proper *Z* stereochemistry in later studies.[^202^, ^203^] In consequence of this former error, in the three syntheses reported in the 1977–1988 period, the anticapsin enantiomer was actually obtained.[^204^, ^205^, ^206^] The first enantioselective synthesis of anticapsin (Scheme 35) was proposed by Baldwin *et al*.^[207]^ The initial substrate was commercially available optically active monoester **219**, converted through a chiral lactone into the protected alcohol **220**. The **220** intermediate was decarboxylated and then the silyl diether was transformed to the iododerivative **221**. Alkylation of **221** with lithium cyanocuprate afforded the coupled product, bislactim ether. Ring opening led to the **222** intermediate, which in the consecutive steps including epoxidation, oxidation to form ketone and deprotection was converted into final product, identical with anticapsin of natural origin.

Wild, independently, presented enantio‐ and diastereoselective syntheses of anticapsin, bacilysin and some of their diastereoisomers. The key steps in that approach were diastereoselective 1,6‐addition of the bislactim ether and stereoselective deprotonation of ketone with lithium (*S*,*S*)‐bis(1‐phenylethyl)amide as a chiral base.^[208]^ All the known methods of anticapsin synthesis have been described in the review paper on naturally occurring epoxycyclohexanes.^[209]^

### 3.6 Amino acid antifungals with unidentified targets

**Figure 11:**
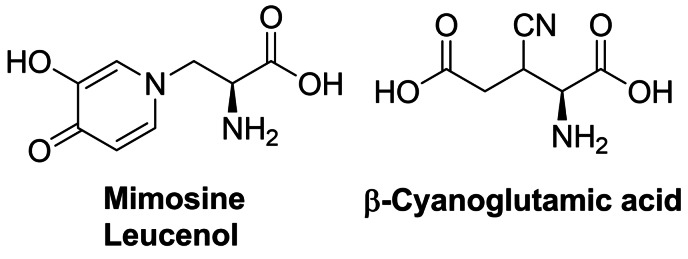
Agents exhibiting antifungal activity with unknown mechanism of action: mimosine and β‐cyanoglutamic acid.

Mimosine (Figure 11), known also as leucenol, is a toxic non‐protein amino acid produced by seeds, leaves and roots of several Fabeceae, including *Mimosa pudica*.^[210]^ This compound is well known for its mammalian toxicity, which is due to the inhibition of DNA replication.^[211]^ An antifungal spectrum of mimosine comprises human pathogenic dermatophytic fungi but does not cover human pathogenic yeasts.^[212]^ Mimosine can be effectively isolated from plants with high yields. In the initial attempts of mimosine synthesis, reactions of 3‐methoxy‐4‐pyridone with α‐acetamidoacrylic acid,^[213]^ bromoacetaldehyde diethylacetal^[214]^ or aminoacetaldehyde diethylacetal^[215]^ were applied. A racemic mimosine was obtained by condensation of 3‐benzyloxy‐4‐pyrone with β‐amino‐α‐tosylaminopropionic acid.^[216]^ Recently, several mimosine‐containing di‐^[217]^ and tetra‐peptides^[218]^ exhibiting interesting biological properties have been obtained.

The β‐cyanoglutamic acid (Figure 11) produced by *Streptomyces* spp.,^[219]^ demonstrates some antifungal activity but neither mechanism of its antifungal action nor any method of its synthesis have not been reported so far.

## 4 Antiprotozoal amino acid‐based agents

### 4.1 Molecular targets for amino acid antiprotozoals

Among molecular targets for antiprotozoal compounds, these present in protozoans responsible for malaria, i. e. *Plasmodium* spp. and for African sleeping sickness – *Trypanosoma* spp., are especially noteworthy.

Ornithine decarboxylase (ODC) catalyzes the decarboxylation of l‐ornithine, derived from the urea cycle, to form putrescine. This reaction is the committed step in polyamine biosynthesis. The enzyme is indispensable to parasites like *Trypanosoma*, *Giardia* and *Plasmodium*. ODC is a molecular target for α‐difluoromethyl‐l‐ornithine (Eflornithine). Farnesyltransferase is an enzyme modifying posttranslationally other proteins by adding farnesyl, a 15‐carbon isoprenoid moiety to the thiol functionality of the cysteine residue present in CaaX (“a” is an aliphatic amino acid and “X” is variable) C‐terminal sequence. The proteins enriched with highly lipophilic farnesyl hydrocarbon chain become membrane‐associated and are mostly involved in cellular signalling pathways related to cell growth and proliferation. Note‐ worthy is the fact that inhibition of farnesyltransferase activity in plasmodial cells results in strong growth inhibitory effect, whereas is well tolerated in mammals.^[220]^

The main source of vital amino acids for *Plasmodium* spp. during infection is haemoglobin from the host blood. Specific plasmodial proteases participating in haemoglobin degradation during erythrocyte invasion are promising drug targets. Among them, cysteine proteases – falcipains and aspartic proteases – plasmepsins are of particular interest.^[221]^

Well known target for antiplasmodial compounds is formation of hemozoin, porphyrin‐based non‐toxic polymeric structure, produced by plasmodial hemopolymerase from the digested host hemoglobin. Formation of hemozoin is essential to the survival of *Plasmodium* spp. and as such, is a target for antimalarial drugs, like chloroquine and mefloquine.^[222]^

### 4.2 The antitrypanosomal amino acid used in clinical practice

**Scheme 36:**
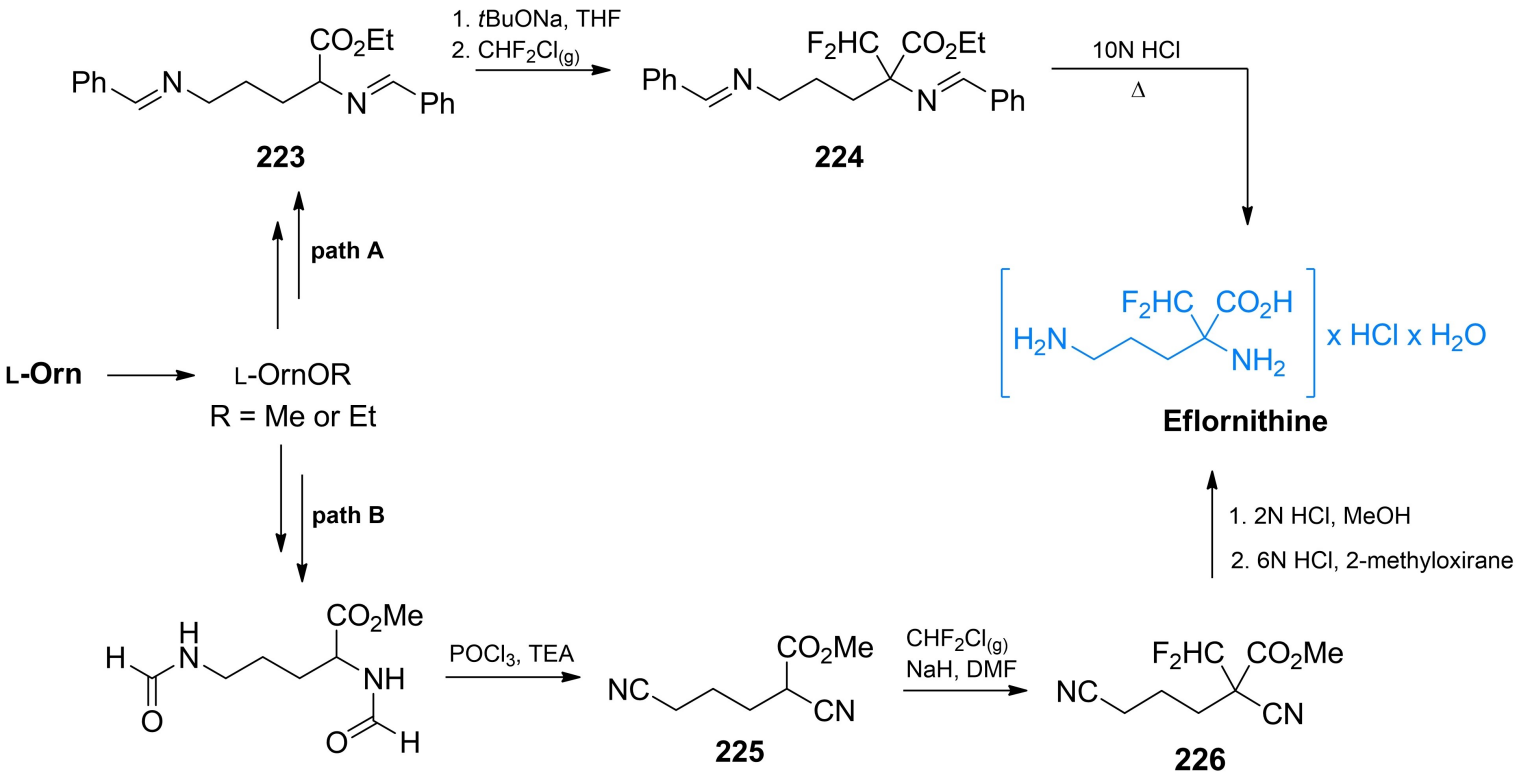
Synthesis of Eflornithine from l‐ornithine.

One of the very few medicines that have been successfully developed by rational drug design is α‐difluoromethyl‐l‐ornithine, known as Eflornithine (Scheme 36). This ornithine analogue is an active component of the cream drug sold under the brand name Vaniqa and of the injectable medicine, Ornidyl. The former is a medication used to treat excessive hair growth on the face in women (hirsutism) and the latter is effective in the 2^nd^ stage of sleeping sickness (African trypanosomiasis). However, Eflornithine is also active against several human pathogenic yeasts, including *C. albicans*, *C. krusei* and *C. neoformans*. Mechanism of action of Eflornithine is well known. This compound is a suicide inhibitor of ornithine decarboxylase, *i. e*. an enzyme catalyzing the crucial step in the pathway of polyamine biosynthesis. Eflornithine irreversibly binds to the cysteine residue located at the enzyme active site. After binding to the active site, it undergoes decarboxylation in a way analogous to that of the natural enzyme substrate and the electrophilic carbon atom created at α‐difluoromethyl group after release of CO_2_ and one of the fluoride atoms, is attacked by the neighboring thiol group of Cys‐360 residue.[^223^, ^224^] More information on biological effects and medical applications of Eflornithine can be found in other reviews.[^225^, ^226^]

There are two main approaches to Eflornithine synthesis; the one starting from ornithine and another one based on condensation reactions. The former approach was applied in the first syntheses.[^227^, ^228^, ^229^] The key step in this approach is a regioselective alkylation of the Schiff's base **223** prepared from l‐ornithine with chlorodifluoromethane, with the formation of a α‐halogenomethylated adduct **224**. Subsequent removal of the protective groups under acidic conditions affords α‐difluoromethylornithine hydrochloride monohydrate (Scheme 36, path A). It has been suggested that difluorocarbene is formed as an intermediate during alkylation.^[227]^ Seki *et al*. proposed another version,^[228]^ in which the key step is diformylation and subsequent dehydration of L‐ornithine methyl ester (Scheme 36, path B). The product **225** is alkylated with difluorochloromethane to give **226** which is subsequently hydrolyzed, thus affording α‐difluoromethylornithine hydrochloride monohydrate.

**Scheme 37:**
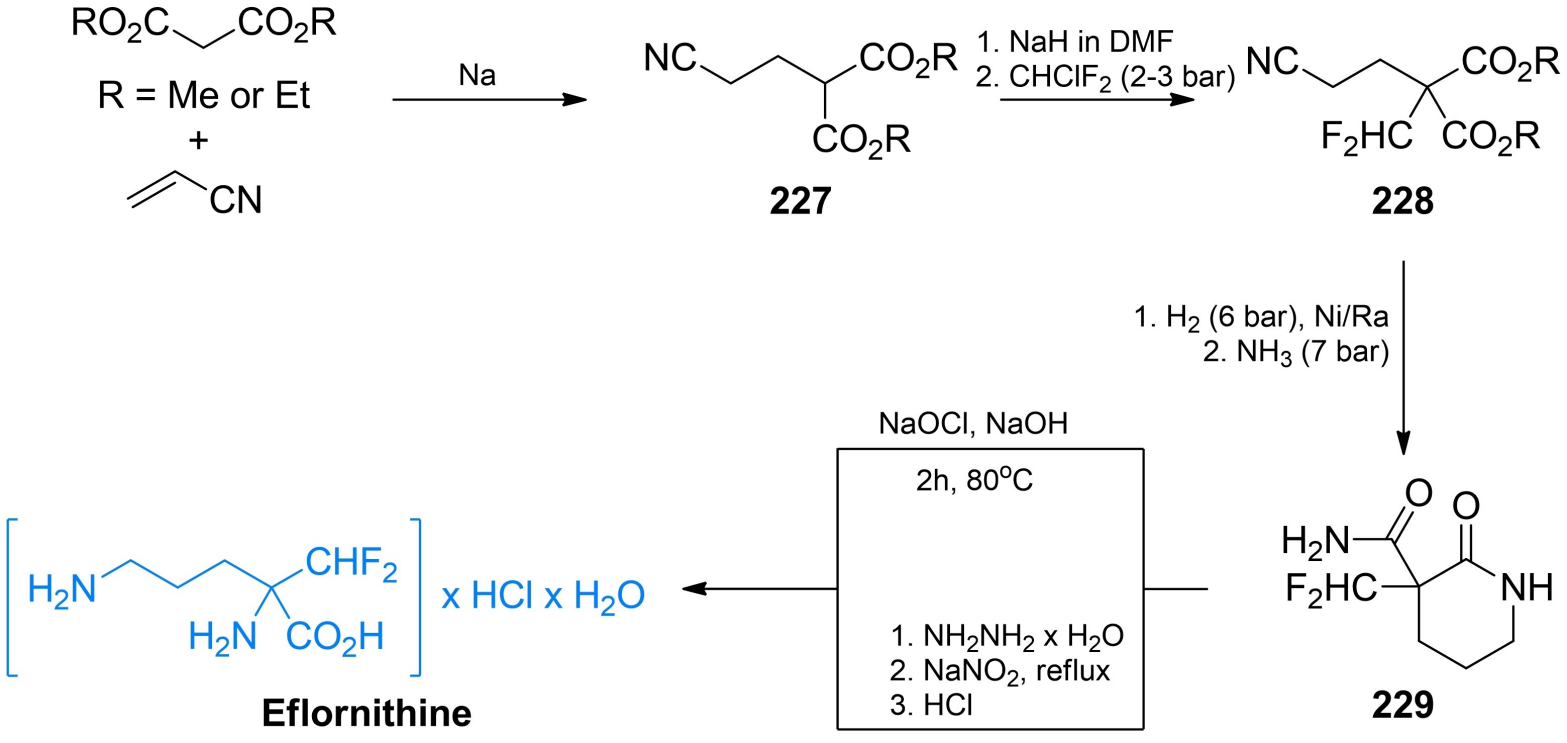
Industrial synthesis of Eflornithine starting from dialkyl malonate.

The condensation‐based approach to eflornithine synthesis is favored in industrial practice. In one of these methods, the first step is a Michael condensation of diethyl malonate with acrylonitrile.[^230^, ^231^] The thus formed diethyl (2‐cyanoethyl)malonate **227** is then fluoromethylated with chlorodifluoromethane under alkaline conditions.[^230^, ^232^] In the next step, hydrogenation of **228** over Raney nickel leads to the δ‐lactam formation, which upon treatment with ammonia is converted into amide **226**. Finally, the Hoffmann rearrangement of **229** and subsequent lactone hydrolysis afford α‐difluoromethylornithine hydrochloride monohydrate (Scheme 37).

**Scheme 38:**
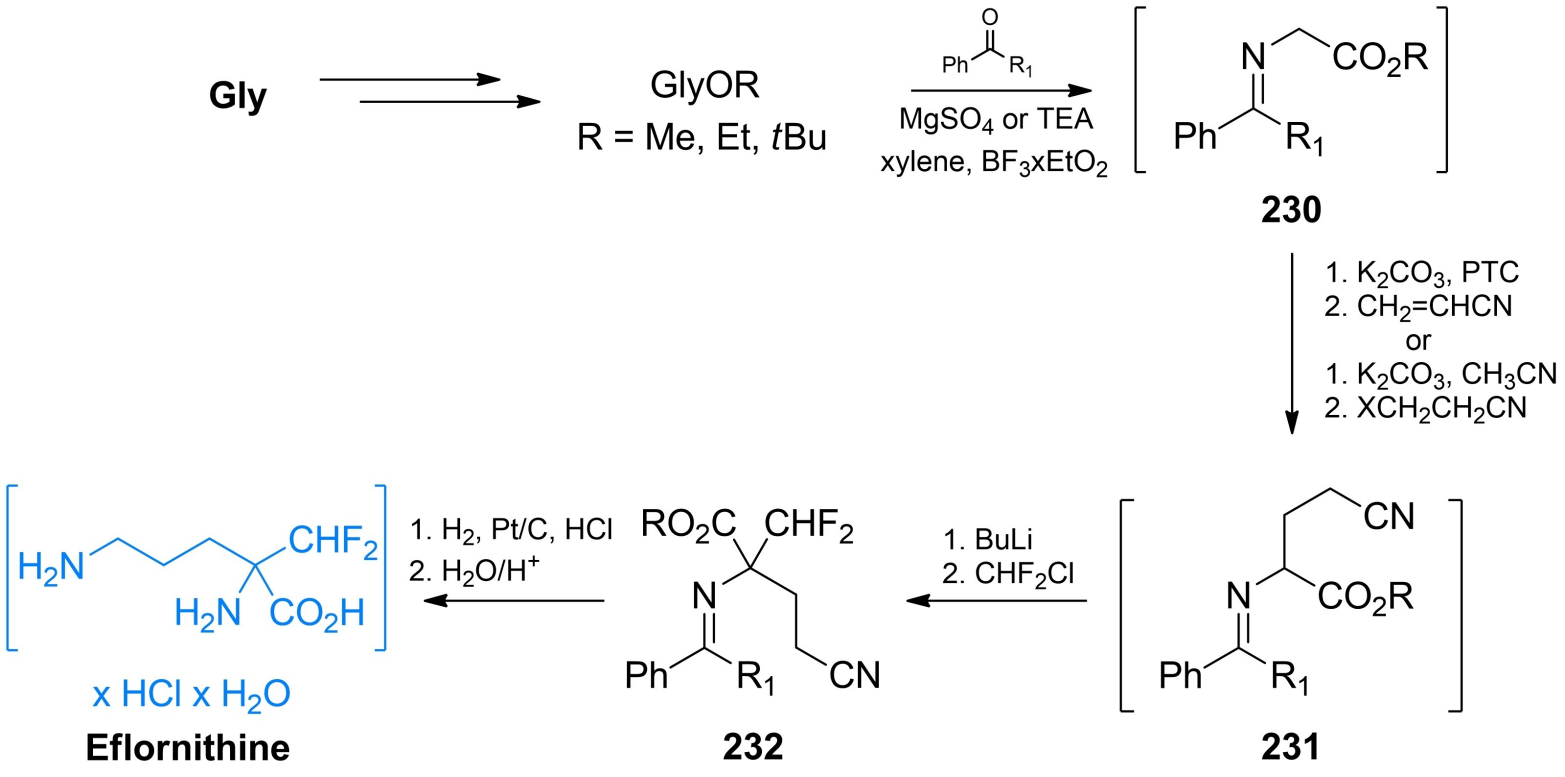
Industrial synthesis of Eflornithine starting from glycine.

An alternative industrial synthesis of eflornithine was described by Zhu *et al*.^[233]^ As shown in Scheme 38, the starting substrate is glycine, the carboxyl group of which is converted into an alkyl ester and the amino group into the Schiff's base. The glycine derivative **230** is treated witch acrylonitrile in the presence of a base and a phase transfer catalyst to give the **231** adduct. This adduct is alkylated with halodifluoromethane in the presence of BuLi. The nitryl group of **232** is reduced and protective groups are removed thus affording difluoromethylornithine hydrochloride monohydrate.

Because chlorodifluoromethane is no longer allowed for industrial applications according to the Montreal protocol, the recommended alkylating agent in eflornithine synthesis is fluoroform, formed as a side product in polytetrafluoroethylene synthesis. Due to the low reactivity of fluoroform, difluoromethylation with this reagent must be performed in continuous flow.[^231^, ^233^, ^234^]

### 4.3 Antiplasmodial agents incorporating protein amino acids residues

Several antiplasmodial agents incorporating proteinogenic amino acid residues, especially l‐phenylalanine, l‐leucine, and glycine have been reported. Most of them target plasmodial protease falicipain‐2. Depending on the chemical structure, inhibitors of falcipain‐2 could be divided into three main groups :  (i) peptide‐based inhibitors, (ii) peptidomimetic inhibitors, and (iii) nonpeptidic inhibitors.

**Figure 12:**
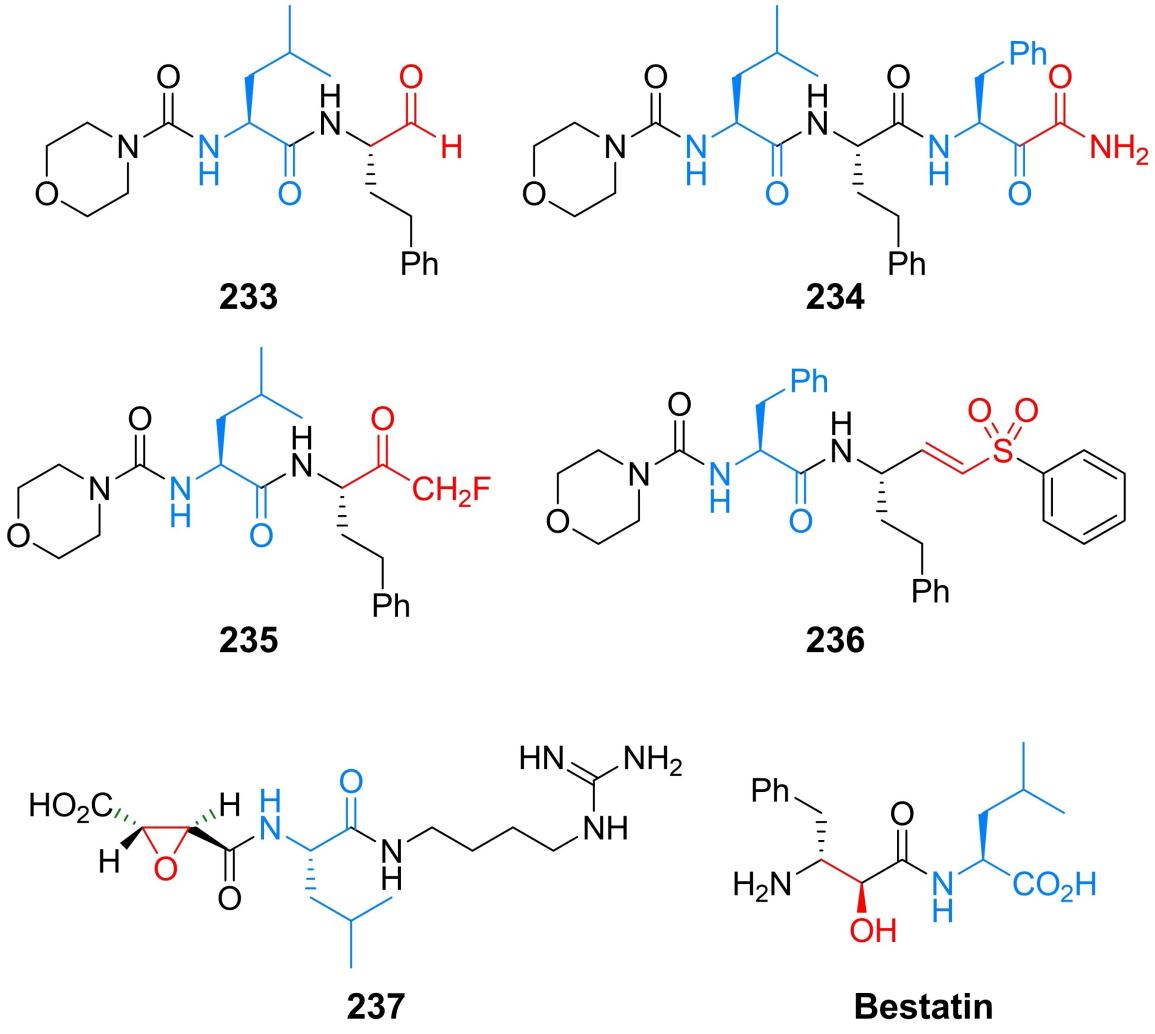
Examples of different classes of plasmodial peptidic proteases inhibitors: aldehyde‐based **233**, α‐ketoamide‐based **234**, fluoroketone‐based **235**, vinyl sulfone‐based **236**, succinyl epoxide‐based **237**, and bestatin. Electrophilic warheads of compounds **233–237** and transition state bioisostere of bestatin are marked in red.

Peptide‐based inhibitors of falcipains incorporate proteinogenic amino acid residues, stabilizing inhibitor at the protease active site. Among them, fluoromethyl ketones,^[235]^ vinyl sulfones,^[236]^ aldehydes,^[237]^ α‐ketoamides,^[238]^ epoxysuccinyl derivatives and aziridines^[239]^ could be distinguished. Aldehyde‐ and α‐ketoamides‐based (but not fluoromethyl ketones) inhibitors are covalent reversible inhibitors, forming a thiohemiacetal bond with the serine residue. Fluoromethyl peptidyl derivatives incorporate highly electrophilic α‐fluoroketone functionality or vinyl sulfones carrying activated electrophilic α,β‐unsaturated double bond, responsible for the formation of the covalent bond with protease's serine residue. Succinic epoxide derivatives and aziridine derivatives are spring‐loaded molecules incorporating highly electrophilic three‐membered rings. In addition to electrophilic moiety responsible for covalent bond formation with active centre serine, all of mentioned groups inhibitors, except aziridines, possess l‐phenyl alanine or l‐leucine residues, responsible for inhibitor stabilization at the target enzyme. Bestatin is an effective inhibitor of plasmodial proteases based on the l‐leucine residue carrying transition state bioisostere (Figure 12). The syntheses of all inhibitors mentioned above (except aziridines which will be discussed later) utilize readily available proteinogenic amino acids.

**Scheme 39:**
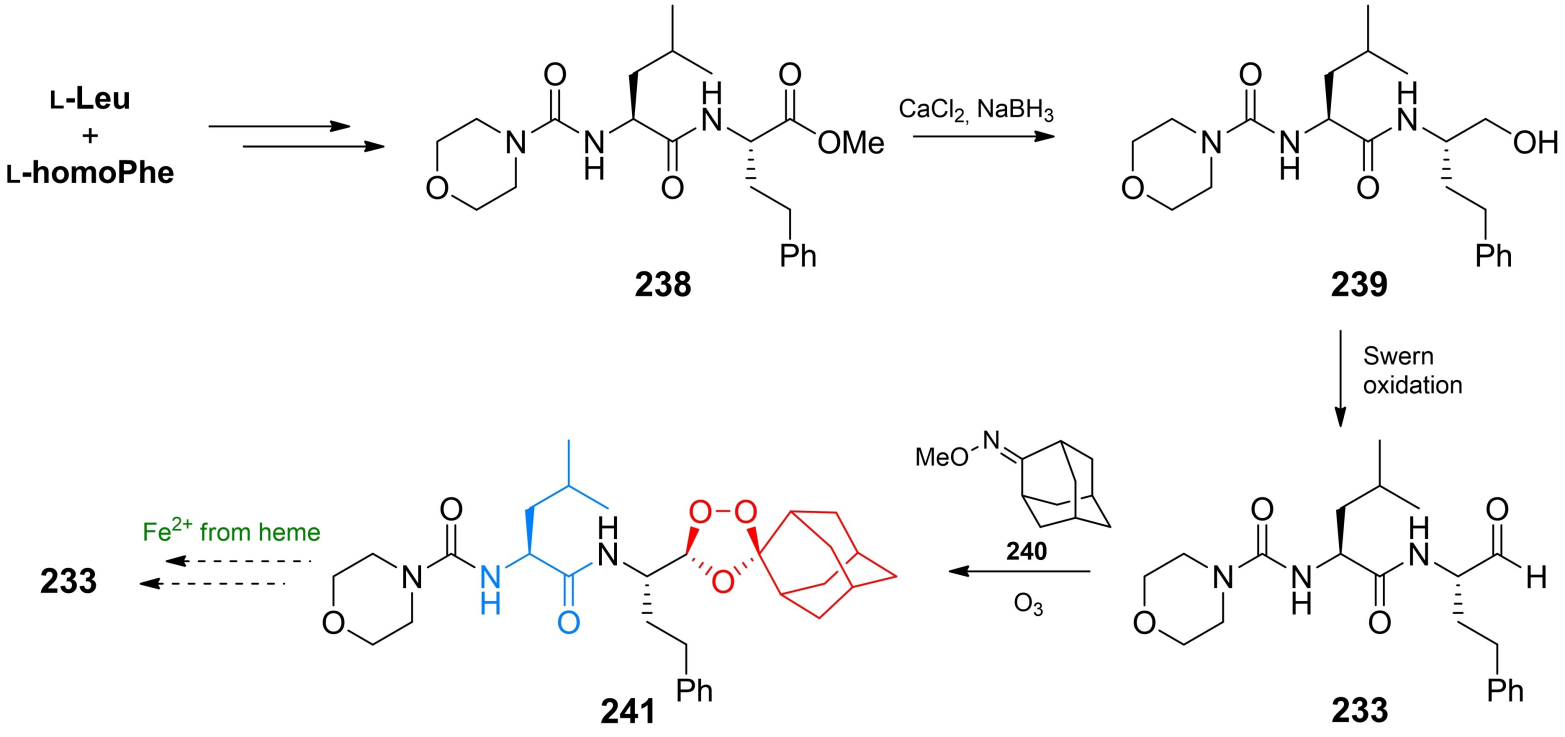
Synthesis of an antiplasmodial 1,2,4‐trioxolane prodrug of inhibitor **233**.

Recent and very interesting advances in aldehyde‐based inhibitors were reported by Gibbons and co‐workers.^[237]^ In their work, the synthesis and biological evaluation of 1,2,4‐trioxolane prodrug **241** (Scheme 39) of aldehyde‐based inhibitor **233** were described. The prodrug structure was designed in the way allowing the selective release of active aldehyde form only in parasite food vacuoles where the concentration of Fe(II) iron ions is on high level. The trioxide structure is reduced to radical species which undergoes subsequent decomposition to an aldehyde inhibitor **233**. The synthesis of prodrug begins with readily available l‐leucine and l‐homophenylalanine, which are converted to the appropriate dipeptide **238**. Reduction of ester moiety and subsequent Swern oxidation of resulting primary alcohol **239** give aldehyde‐based inhibitor **233**. Formation of trioxolane prodrug was accomplished by reaction of **233** with adamantane *O*‐methyloxime **240** in the presence of ozone resulting in prodrug **241** (Scheme 39).^[237]^

**Scheme 40:**
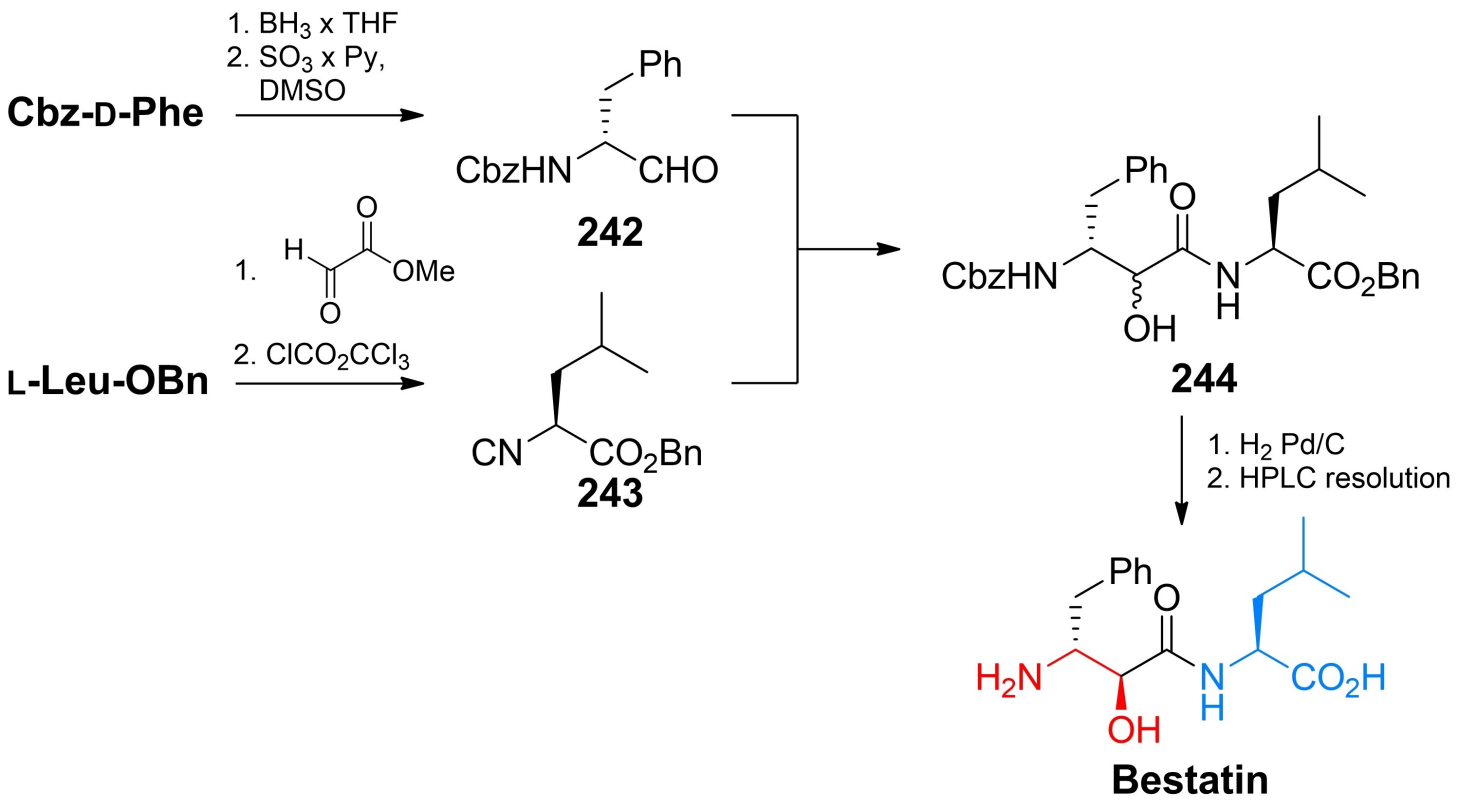
Synthesis of bestatin according to Semple *et al*.^[238]^ Transition state bioisostere of bestatin is marked in red.

The mild‐condition synthesis leading to β‐hydroxyamide, bestatin and β‐ketoamides in moderate to high yields was proposed by Semple and co‐workers.^[238]^ In their approach, *N*‐protected α‐amino aldehydes obtained from readily available amino acids and isonitriles are used. The synthesis of bestatin proposed by Semple *et al*. starts with Cbz protected d‐phenylalanine which is reduced with BH_3_ and then oxidised to aldehyde **242** with SO_3_/pyridine complex. The aldehyde is subjected to reaction with isonitrile freshly prepared from commercially available benzyl ester of l‐leucine hydrochloride. The resulting diastereoisomeric mixture of α‐hydroxyamide product **244** after HPLC resolution afforded bestatin. Moreover, further oxidation of α‐hydroxyamides could lead to α‐ketoamide derivatives (Scheme 40).^[238]^

**Scheme 41:**
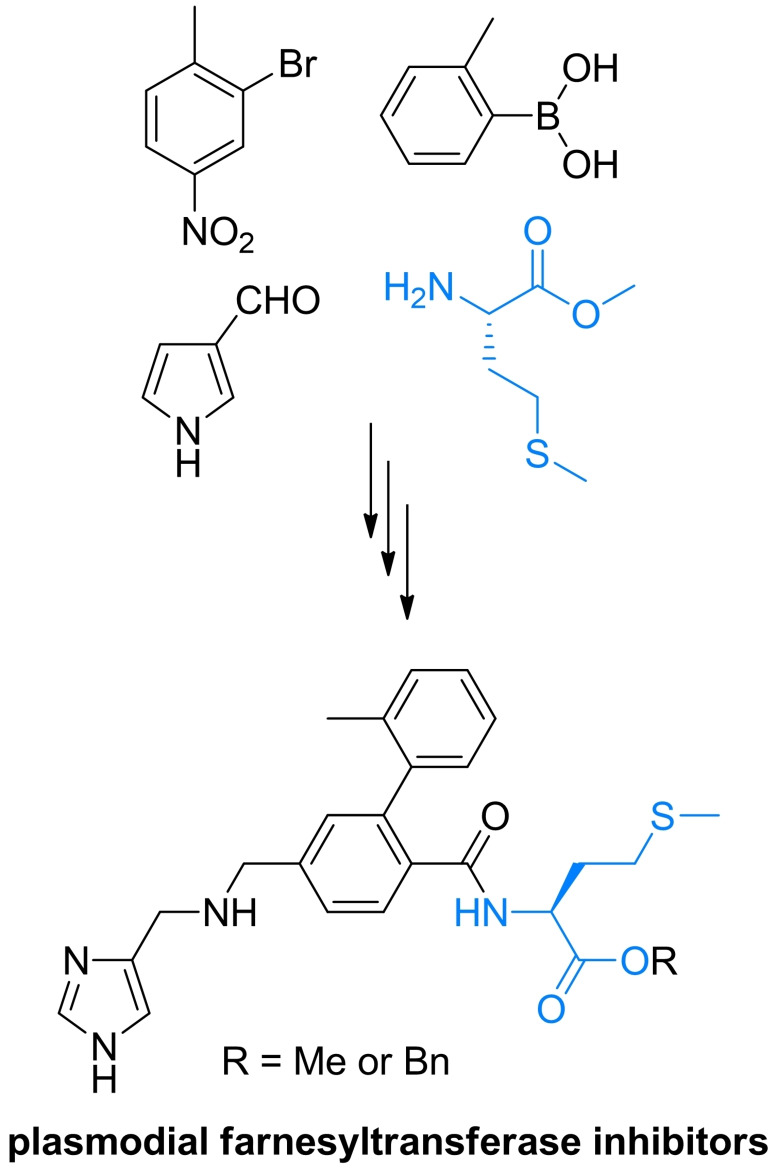
Chemical structure and chemical components of plasmodial farnesyltransferase peptidomimetic inhibitors incorporating l‐methionine residue.

Nowadays several groups of plasmodial farnesyltransferase inhibitors, based on different scaffolds have been proposed.^[220]^ Among them, peptidomimetics structures incorporating l‐methionine residue have been reported. The research group of Hamilton has designed, synthesized and evaluated a series of potential peptidomimetic inhibitors, out of which two of them exhibit significant antiplasmodial activity (Scheme 41). The synthetic approach leading to both farnesyltransferase inhibitors compounds is straight forward and could be accomplished starting from readily available reagents.[^240^, ^241^]

**Figure 13:**
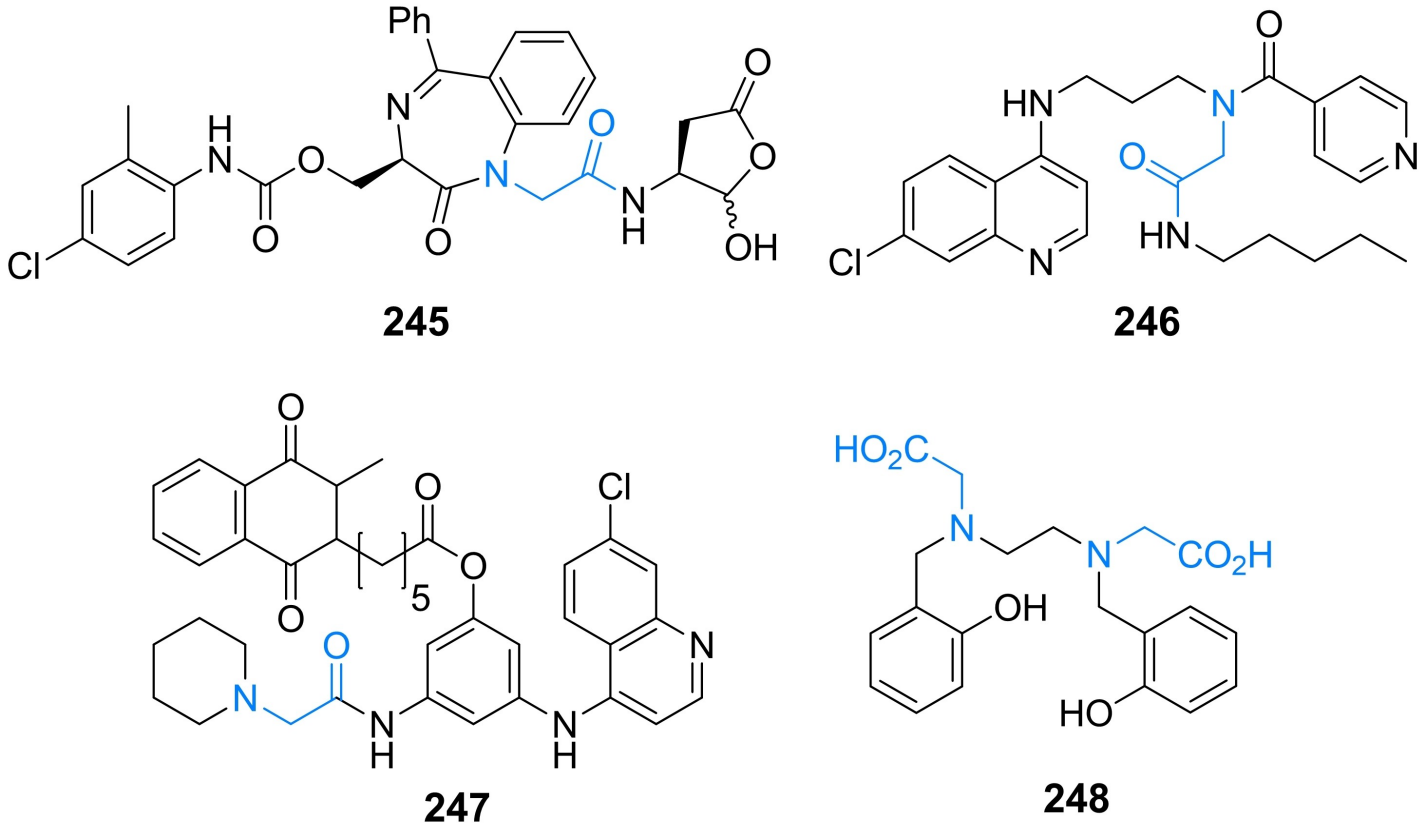
Antiplasmodial agents incorporating glycine or glycine‐based residues: peptidomimetic protease inhibitor **245**, heme interacting agents **246** and **247**, iron chelator **248**.

The simplest amino acid structure of glycine and glycine‐derived residues occur in several antiplasmodial agents. Among them, peptidomimetic inhibitors of proteases, heme interacting agents, and iron chelators could be mentioned (Figure 13).^[222]^

### 4.4 Antiprotozoal agents incorporating non‐proteinogenic amino acids residues

Spring‐loaded peptidyl aziridines are potent inhibitors of protozoal proteases inhibitors mainly based on aziridine‐2‐carboxylic acid or aziridine‐2,3‐dicarboxylic acid structures with *R,R* or *S,S* configuration of the aziridine ring.^[242]^

**Scheme 42:**
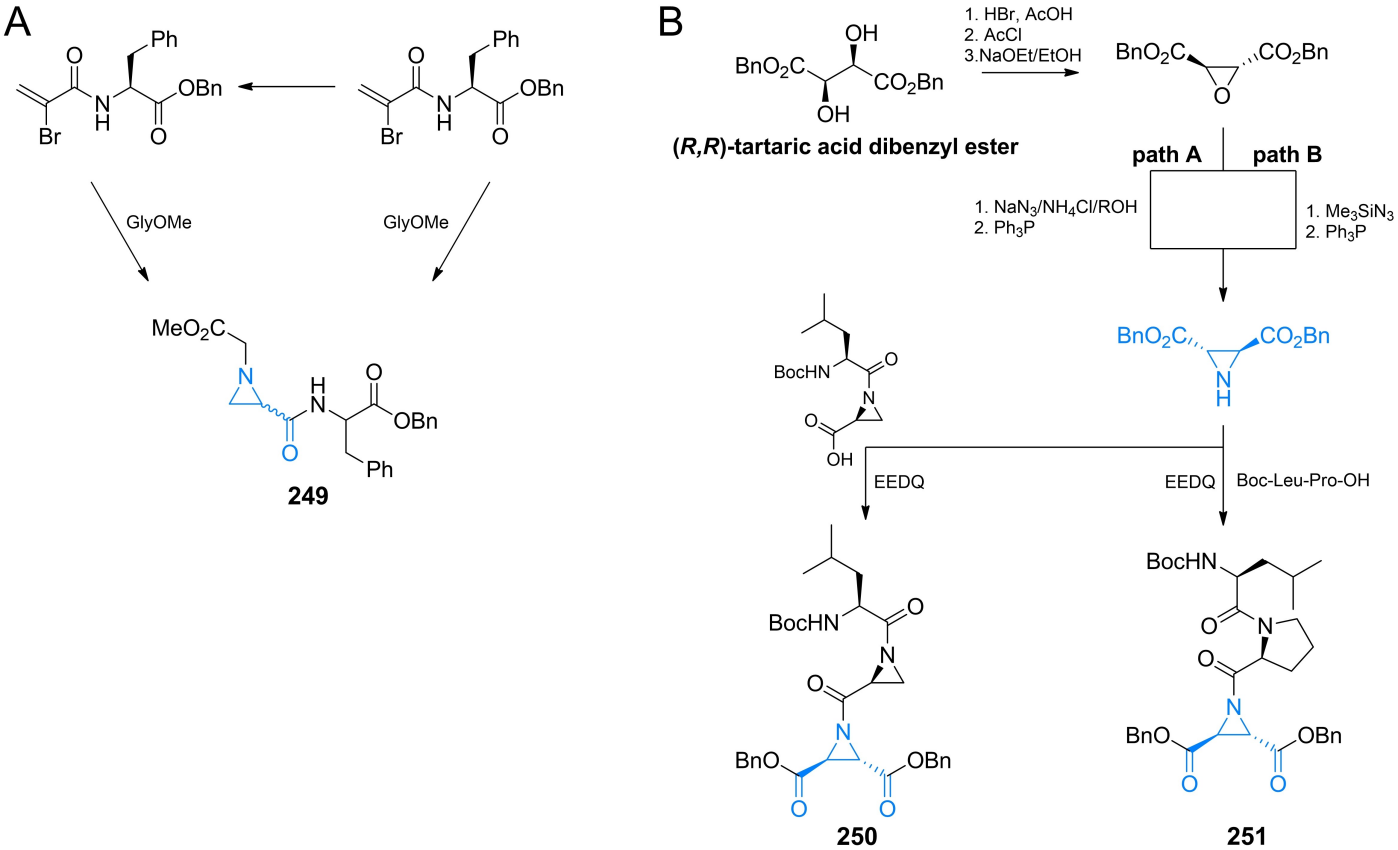
Synthesis of peptidyl aziridines plasmodial proteases inhibitors :  (A) synthesis of diastereomeric *N*‐alkylated aziridine‐2‐carboxylic acid‐based inhibitor **249**; (B) synthesis of *N*‐acylated aziridine‐2,3‐dicarboxylic acid‐based inhibitors **250** and **251**.

Schultz and co‐workers^[239]^ have studied an inhibitory effect of 77 peptidyl aziridines against plasmodial proteases falcipain‐2 and falcipain‐3. Among them three derivatives have shown significant inhibitory activity against mentioned enzymes. These compounds are *N*‐alkylated aziridine‐2‐carboxylic acid l‐Phe derivative **249** and *N*‐acylated aziridine‐2,3‐dicarboxylic acid benzyl esters **250** and **251**. According to Schultz *et al*. inhibitor **249** could be obtained upon condensation of *N*‐substituted l‐phenylalanine benzyl ester with glycine methyl ester followed by cyclization reaction leading to diastereomeric mixture of product **249** (Scheme 42A).^[239]^

Syntheses of inhibitors **250** and **251** calls for diastereomerically defined *trans*‐aziridine‐2,3‐dicarboxylic acid benzyl esters, which could be obtained from (*R,R*) or (*S,S*)‐tartaric acid benzyl esters. The asymmetric synthesis of inhibitors **250** and **251**, proposed by Breuning *at al*. is presented in Scheme 42B.^[243]^

Other antiprotozoal cysteine protease inhibitors based on aziridine‐2,3‐dicarboxylic acid were reported. Among them, inhibitors demonstrating antitrypanosomal^[244]^ and antileishmanial^[245]^ activity were described.

## 5 Conclusions

The amino acid motif is present in a vast number of antimicrobial agents. In some of these compounds, structure of which mimics the transition state tetrahedral intermediate of enzymatic reaction, the carboxylate functionality is substituted by phosphonate or boronate groups. Amino acid antibacterials target particular enzymes involved in formation and incorporation of d‐amino acids as components of cell wall peptidoglycan and most of antifungal amino acids are inhibitors of fungi‐specific pathways of biosynthesis of particular protein amino acids. Three different proteins have been identified as targets for antiprotozoal amino acids. Two amino acid antimicrobials, namely antibacterial d‐cycloserine and antiprotozoal Eflornithine, are used as drugs in antimicrobial chemotherapy. The latter is the exceptional example of a rationally designed enzymatic suicide inhibitor successfully introduced to clinics. Synthesis of amino acid antimicrobials is a particular challenge, especially in terms of the need for stereoselective, including enantioselective methods.

In our opinion, low antimicrobial activity of at least some of the amino acid agents could be due to their poor uptake by microbial cells. This might be improved by application of the pro‐drug or “portage transport” approach, i. e. conversion of amino acid into a more lipophilic derivative or conjugation with molecular nanocarriers, like cell penetrating peptides or dendrimers.[^246^, ^247^] Moreover, due to the emerging challenges of microbial drug resistance, there is still room for identification of novel molecular targets for amino acid antimicrobials and for the rational design of more effective inhibitors of already known targets.

## Abbreviations

BOP: benzotriazol‐1‐yloxytris(dimethylamino)phosphonium hexafluorophosphate
Cbz: (benzyloxy)carbonyl group
cHex: cyclohexyl group
DBU: 1,8‐diazabicyclo(5.4.0)undec‐7‐ene
DCM: dichloromethane
DDQ: 2,3‐dichloro‐5,6‐dicyano‐1,4‐benzoquinone
DIBAL: diisobutylaluminium hydride
DIPEA: *N,N*‐diisopropylethylamine
DMAD: dimethyl acetylenedicarboxylate
DMAP: 4‐dimethylaminopyridine
Dppf: 1,1‐bis(diphenylphosphino)ferrocene
EEDQ: *N‐*ethoxycarbonyl‐2‐ethoxy‐1,2‐dihydroquinoline
*Hlq*
: chlorine or bromine
HMDS: 1,1,1,3,3,3‐hexamethyldisilazane
HMPA: hexamethylphosphoramide
HSAc: thioacetic acid
Ms: methanesulfonyl group
MSA: methanesulfonic acid
NMM: *N*‐methylmorpholine
NMMO: *N*‐methylmorpholine *N*‐oxide
Phth: phtaloyl group
Pip: piperidine
PPTS: pyridinium toluene‐*p*‐sulfonate
PTSA: *p*‐toluenesulfonic acid
TBAF: tetrabutylammonium fluoride
TBDMS: *tert*‐butyldimethylsilyl group
TBTU: 2‐(1*H*‐Benzotriazole‐1‐yl)‐1,1,3,3‐tetramethylaminium tetrafluoroborate
TBAF: tetrabutylammonium fluoride
TEA: triethylamine
TEBA: triethylbenzylammmonium chloride
Teoc: 2‐(trimethylsilyl)ethoxycarbonyl group
TES: triethylsilyl group
TFA: trifluoroacetic acid
TFAA: trifluoroacetic acid anhydride
TMS: trimethylsilyl group,
Ts: *p*‐toluenesulfonyl group

## Conflict of interest

The authors declare no conflict of interest.

## Biographical Information

*Michał G. Nowak was born in Gdańsk, Poland, in 1992. He studied at Gdańsk University of Technology, Poland, and received an MSc degree in chemistry in 2018. In the same year, he started a PhD study at the same university. Currently, he is working in an interdisciplinary research group, focusing on antimicrobial agents. His research interests include the synthesis of biocleavable conjugates incorporating microbial enzymatic inhibitors and “trimethyl lock” systems*.

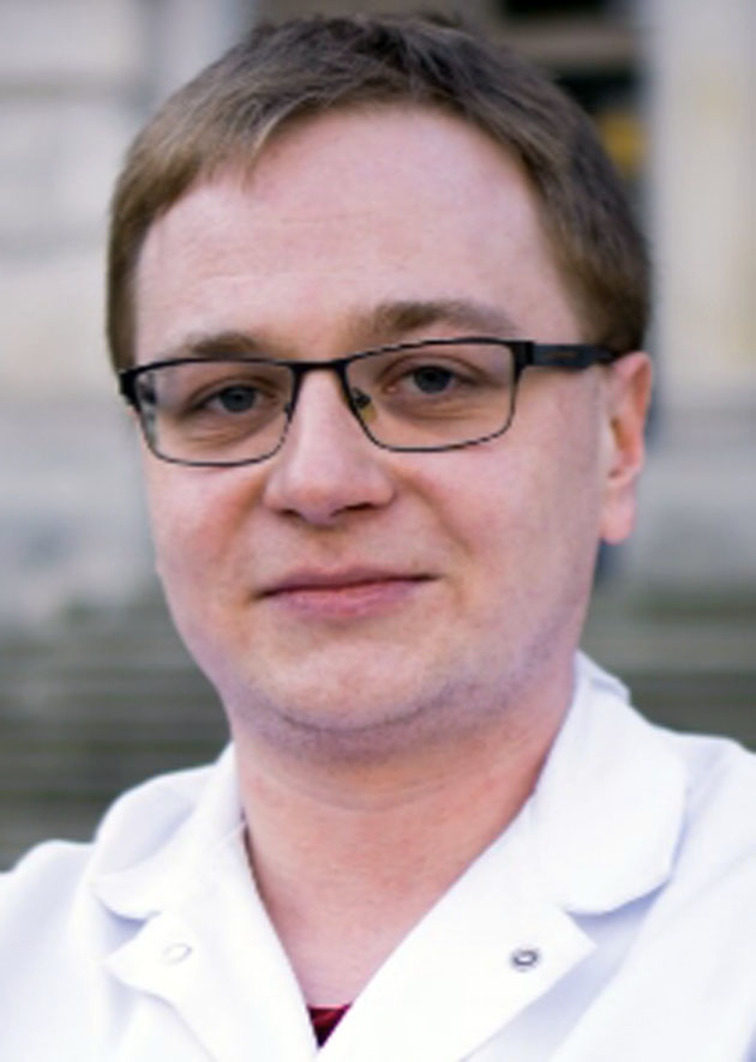

## Biographical Information

*Andrzej S. Skwarecki was born in Lipno, Poland, in 1989. He studied at the Gdańsk University of Technology, Poland, and received an MSc degree in pharmaceutical biotechnology in 2013 and a PhD in 2017 in organic chemistry. His main research interests include organic synthesis of compounds with potential antimicrobial activity and application of “molecular umbrellas” carriers in antifungal agents delivery*.

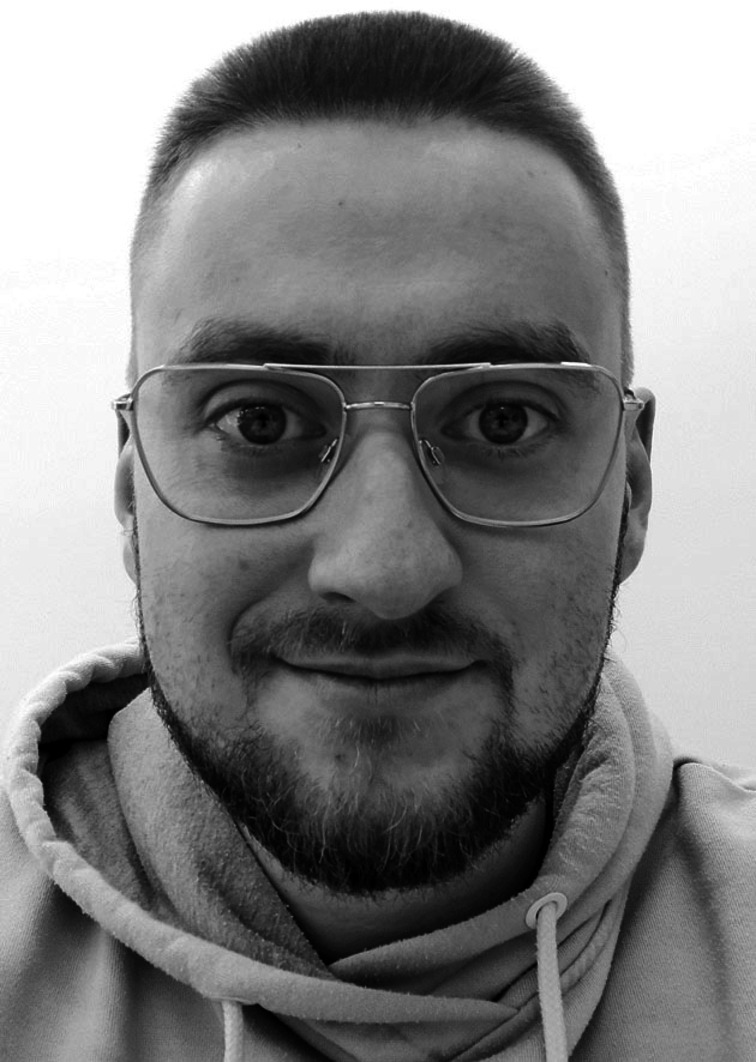

## Biographical Information

*Maria J. Milewska received her PhD from Gdańsk University of Technology in 1985 with prof. Andrzej Chimiak. For several years she was interested in synthesis of iron complexing compounds and then in supramolecular chemistry. In 2013 she became a professor in chemical sciences. She is currently a head of Department of Organic Chemistry at the Faculty of Chemistry, Gdańsk University of Technology. Her group works on rational design and synthesis of drug candidates for antimicrobial chemotherapy*.